# Supplementary material for: Approaches to enabling rapid evaluation of innovations in health and social care: a scoping review of evidence from high-income countries
Source: BMJ Open. 2022 Dec 20;12(12):e064345. doi: 10.1136/bmjopen-2022-064345 (PMC10580278; doi:10.1136/bmjopen-2022-064345)
Supplement: Supplementary data [file bmjopen-2022-064345supp001.pdf]

Appendix: Medline Search for rapid evaluations

Rapid evaluations

Database: Ovid MEDLINE(R) and Epub Ahead of Print, In-Process & Other Non-Indexed Citations and Daily <1946 to September 13, 2022>
Search Strategy:
----
1 rapid evaluation.tw. (1469)
2 (Rapid adj2 (assessment* or report* or qualitative research or research or studies or study or evaluation* or
appraisal* or approach* or response brief* or response program*)).ti. (2911)
3 ((Fast or faster) adj (assessments or reports or qualitative research or research or studies or evaluations or
appraisals or approaches or response briefs or response program*)).tw. (87)
4 ((limit* or short* or tight or efficient or pragmatic) adj2 (deadline* or time or timescale or timeline or time
period or time frame) adj2 (assessments or reports or qualitative research or research or studies or evaluations or
appraisals or approaches or response briefs or response program*)).tw. (275)
5 ((Speed or speedy) adj (assessments or reports or qualitative research or research or studies or evaluations or
appraisals or approaches or response briefs or response program*)).tw. (88)
6 (Abbreviated adj (assessments or reports or qualitative research or research or studies or evaluations or
appraisals or approaches or response briefs or response program*)).tw. (11)
7 (Accelerated adj (assessments or reports or qualitative research or research or studies or evaluations or
appraisals or approaches or response briefs or response program*)).tw. (152)
8 (Brief adj (assessments or reports or qualitative research or research or studies or evaluations or appraisals or
approaches or response program*)).tw. (494)
9 (constrained by time adj10 (assessments or reports or qualitative research or research or studies or evaluations
or appraisals or approaches or response briefs or response program*)).tw. (3)
10 (Expedited adj (assessments or reports or qualitative research or research or studies or evaluations or
appraisals or approaches or response briefs or response program*)).tw. (14)
11 (Express adj (assessments or reports or qualitative research or research or studies or evaluations or appraisals
or approaches or response briefs or response program*)).tw. (7)
12 (Immediate adj (qualitative research or research or studies or evaluations or appraisals or approaches or

response briefs or response program*)).tw. (61)
13 ((Quick adj2 dirty) and (assessments or reports or qualitative research or research or studies or evaluations or
appraisals or approaches or response briefs or response program*)).tw. (16)
14 (Quick* adj (assessments or reports or qualitative research or research or studies or evaluations or appraisals
or approaches or response briefs or response program*)).tw. (71)
15 (Rapid adj (assessments or reports or qualitative research or research or studies or evaluations or appraisals or
approaches or response brief* or response program*)).tw. (292)
16 (Stream-line* adj (assessments or reports or qualitative research or research or studies or evaluations or
appraisals or approaches or response briefs or response program*)).tw. (1)
17 (Streamline* adj (assessments or reports or qualitative research or research or studies or evaluations or
appraisals or approaches or response briefs or response program*)).tw. (59)
18 (timeliness adj6 (assessments or reports or qualitative research or research or studies or evaluations or
appraisals or approaches or response briefs or response program*)).tw. (211)
19 (Timely adj (assessments or reports or qualitative research or research or studies or evaluations or appraisals
or approaches or response briefs or response program*)).tw. (146)
20 or/1-19 (5967)
21 ((Fast or faster) adj (appraisal or approach or study or assessment or report)).tw. (577)
22 ((limit* or short* or tight or efficient or pragmatic) adj2 (deadline* or time or timescale or timeline or time
period or time frame) adj2 (appraisal or approach or study or assessment or report)).tw. (650)
23 ((Speed or speedy) adj (appraisal or approach or study or assessment or report)).tw. (115)
24 (Abbreviated adj (appraisal or approach or study or assessment or report)).tw. (45)
25 (Accelerated adj (appraisal or approach or study or assessment or report)).tw. (79)
26 (Brief adj (appraisal or approach or study or assessment or report)).tw. (6166)
27 (constrained by time adj10 (appraisal or approach or study or assessment or report)).tw. (3)
28 (Expedited adj (appraisal or approach or study or assessment or report)).tw. (21)
29 (Express adj (appraisal or approach or study or assessment or report)).tw. (56)
30 (Immediate adj (appraisal or approach or study or assessment or report)).tw. (376)
31 ((Quick adj2 dirty) and (appraisal or approach or study or assessment or report)).tw. (23)

32 (Quick* adj (appraisal or approach or study or assessment or report)).tw. (433)
33 (Rapid adj (appraisal or approach or study or assessment or report)).tw. (4268)
34 (Stream-line* adj (appraisal or approach or study or assessment or report)).tw. (3)
35 (Streamline* adj (appraisal or approach or study or assessment or report)).tw. (234)
36 (timeliness adj6 (appraisal or approach or study or assessment or report)).tw. (277)
37 (Timely adj (appraisal or approach or study or assessment or report)).tw. (329)
38 or/21-37 (13624)
39 evidence-based practice/ (9666)
40 evidence-based medicine/ (72011)
41 Decision Making/ (92841)
42 exp Research/mt, st, sn, td [Methods, Standards, Statistics & Numerical Data, Trends] (84281)
43 exp Animals/ not (exp Animals/ and Humans/) (4671550)
44 39 or 40 or 41 or 42 (250808)
45 38 and 44 (214)
46 20 or 45 (6162)
47 46 not 43 (5569)
48 47 and 20*.ed. (3932)

Rapid reviews (Not included in update September 2022 search)

Database: Ovid MEDLINE(R) and Epub Ahead of Print, In-Process & Other Non-Indexed Citations and Daily <1946 to February 24, 2020>
Search Strategy:
----
1 ((abbreviated adj review?) or (abbreviated adj synthes?s) or (accelerated adj2 review?) or (accelerated adj2 synthes?s) or (brief adj synthes?s) or (expedited adj2 review?) or (expedited adj2 synthes?s) or (meta adj method$) or (meta adj evaluat$) or (rapid adj2 review?) or (rapid adj2 assess$) or "rapid health technology assess$" or (rapid adj HTA?) or (rapid adj approach$) or (rapid adj search$) or (realis$ adj approach$) or (realis$ adj evaluat$) or (realis$ adj synthes?s) or (speed$ adj2 review?) or (streamline$ adj2 review?) or (streamline$ adj2 synthes?s) or (stream-line$ adj2 review?) or (stream-line$ adj2 synthes?s) or (fast$ adj2 review?) or (fast$ adj2 synthes?s)).tw. (9897)
2 (rapid adj2 synthes?s).tw. not (ch or cs).fs. (1152)
3 1 or 2 (11015)
4 exp Animals/ not (exp Animals/ and Humans/) (4671550)

5 3 not 4 (9598)

6 5 and ("2014$" or "2015$" or "2016$" or "2017$" or "2018$" or "2019$" or "2020$").ed. (3345)

Appendix 2: Further information on identified studies

Supplementary Table 1: Descriptions for methodologies described as specifically designed to enable rapid evaluation

Name of methodology	Description of methods	Example(s)
Rapid assessment/appraisal procedure/process (RAP) [multiple terms used for variations and adaptations of core methods.]	Exemplifies use of triangulation of multiple data sources (e.g. interviews and surveys with or without additional data sources). Multiple adaptations identified. Use focused on provision and delivery, barriers, facilitators and perceptions of benefit.	Fergus 2010 (1)(adaptation) Kieber-Emmons 2018 (adapted) (2); Wright 2015; (3) Manderson 1992;(4) McMullen 2011(5) (for clinical informatics); Needle 2000/3(6, 7) Adapted for use in evaluation of changes. Holdsworth(8) Used for policy formulation. Peiro(9, 10) Fitch 2000; 2004 Stimson 2006(11-13)Walton 2021(14); Humphries 2021(15); Vindrola-Padros 2022(16) Gawaya 2022(17); Sidhu 2021 (18); Parkinson 2021(19); Watson 2022(20) Limitations(21, 22)
Rapid appraisal [term used for variations and adaptations of core methods]	Interviews with range of individuals, documentary evidence, focused ethnographic visits, survey; thematic analysis. Not all methods used in all iterations. Related to methods of rapid assessment and response and rapid assessment, response and evaluation.(23-27) Use focused on views or assessments of policies or services.	Green 2015(28) (refs Beebe 2012) (29) Hamilton 1993(30) Rowa-dewar 2008 (31) Harvey 2001(26, 27) Murphy 2018(32)
Rapid evaluation approach of an emergent program	Semi-structured interviews with multiple types of stakeholders; triangulation of data from different stakeholders using same methods; thematic analysis. Focus on service user experiences.	Foreman-Mackey 2019(33, 34) (refs Beebe 2012 (29))

Rapid participatory appraisal	Adaptation of rapid appraisal to involve community in design of appraisal study; includes use of consecutive studies in same community. Use focused on access (and barriers to access) to services; perspectives, experiences and satisfaction relating to services.	Brown 2006 (35) Lazenblatt 2001 (36) Murray 1994, 1999(24, 25), Annett 1995 (guidance) (37)
Rapid participatory appraisal with meta-planning	Uses peer facilitation of data collection; triangulates two separate groups of participant responses.	Lawlor 1999 (38)
Rapid ethnographic assessment/appraisal /Rapid ethnography	Assessment of intervention in naturalistic setting. Interviews, focus groups, observations of implementation, analysis of routine data sources (not all methods in all studies). Typically used interview and survey data, triangulated. Includes but is not limited to evaluation of services; focus on views and experiences. Question mark over whether truly ethnographic. (39, 40)	Hanckel 2019(41-44) Boyd 2018 (42) Goepp 2008,(43) Green 2013(44) Ackerman 2017(45) Quintero-Romero;(46) Kroeger 2013(47) Ayele 2021; Manges 2021(48, 49) Betsos 2021(50); Collins 2019;(51) Collins 2022(52); Oh 2021(53)
Rapid assessment and response (RAR)	Interviews with representative individuals/ groups (also described as focus groups), integrates findings with data from existing records and desk research; researcher training required for effectiveness. Focus on knowledge and service capacity and development	Dupont 2015;(54) Stimson 2006(13) Ong 1996, 1991(55, 56) WHO 2002 (guidance for psychoactive drugs and sexual risk behaviours) (57)
Rapid assessment, response and evaluation (RARE)	A model for using RAR within context of public health policy development; (58) RAR in implementation evaluation. Substantive training component for proper implementation. Rapid qualitative ethnographic methods: use of existing data; direct involvement of community leaders/health providers; explicit evaluation component. Focus on care coverage, access and barriers.	Trotter 2001(58) Needle 2000,2003(6, 7) Brown 2008(59)Burks 2011(60) Valderrama 2006 (61)(combined with policy level surveying)

Rapid cycle evaluation; Rapid feedback evaluation	Use of consolidated framework for implementation research (CFIR) using observational field notes and semi-structured interviews across multiple sites to enable rapid cycle data analysis and generate actionable findings during the research project life cycle. Using Expert recommendations for implementing change (ERIC) (34, 62) Identifies barriers and facilitators to components of initiatives.	Zakocs 2015; Keith 2017; Skillman 2018; Shrank 2013; Cohn 2021; Ohrling 20202; Last 2021: Williams; 2021 (63-69)
Plan-do-study-act cycles	Method drawn from quality improvement.<br>Small-scale implementation and evaluation of changes, with weekly or monthly (e.g.) tracking of routinely collected data at ward/hospital/district level. Shares some features with studies using real-time feedback in other designs.(70)	Farrer 2014; Kercsmar 2017 (71, 72)
Rapid cycle evaluation building in plan do study act (RAPID)	Method to support decision making based on rapid cycle evaluation of electronic data	Schneeweiss 2015(73)
Rapid assessment procedure informed clinical ethnography (RAPICE)	Mixed methods approach using a combination of rapid appraisal and ethnography to understand implementation processes. Linked to a pragmatic trial; focused on barriers and facilitators.	Palinkas 2019a/b; Palinkas 2022; Moloney 2020 (74-77)
Focused rapid assessment process	Place-focused adaptation of rapid assessment process; combining geospatial mapping and qualitative assessment.	Kieber-Emmons 2022(78)
Qualitative rapid assessment	Focus groups and semi-structured interviews; thematic analysis; focus on acceptability and feasibility.	Guise 2018(79)
Qualitative rapid appraisal, Rigorous analysis (Q-RARA)	Qualitative mixed methods: site visit, semi-structured interviews, observations, photographs, floor plans, and social scanning data; integrated qualitative analysis approach. Focus on organisational function and processes in small primary health care organisations.	Phillips 2014(80)

Rapid gap assessment approach	Mixed methods involving targeted site identification; checklist-based data collection; notes from participating centres and open ended questions; use of online search methods to identify sites. Approach described as modified rapid gap assessment.	Williams 2019(81)
Swift Worksite Assessment and Translation (SWAT)	Multiple evaluation approaches – site visits, interviews etc. Includes disseminations stage; aims to assess effectiveness of workplace health policies may be suitable for innovations aimed at HCP	Dunet 2008(82)
Pilot implementation	Although we did not include studies described purely as pilots this represented a rapid assessment of implementation of change – assessment undertaken after one week. Similar to plan-do-study-act cycle approaches. Focus on measures of target meeting for triage time.	Gray 2017(83)

Supplementary table 2: Primary studies in the UK

Study ID	Type of study	Rapid descriptor	Study question	Methods used	Data collected
Aspray 2006(84)	Mixed methods	Rapid evaluation	Equity of care diabetes standards in care homes	Interviews, checklists, record review	Quality of care metrics; experience of patients and carers
Banga 2018(85)	Quantitative	Rapid safety evaluation	Coverage and AE in influenza vaccination	Enhanced passive surveillance, spontaneous reporting, usage data	Uptake and AE (especially reporting within 7 days)
Begg 1989(86)	Quantitative	Rapid evaluation	Vaccination coverage of sentinel vaccines at key timepoints	Analysis of quarterly data cohorts	Proportion of eligible children immunised

Brook 2011(87)	Quantitative	Rapid cycle assessment	Sexual health clinic service evaluation	Electronic patient record analysis	Proportion of people treated and partners tested
Brown 2006(36)	Qualitative	Rapid participatory appraisal	Healthy environment - perceptions of need and concern	Documentation review, observations, interviews	Perceived needs and concerns around health-related needs (environment-based)
Cake 2022(88)	Quantitative	Rapid data-enabled access	Early recruitment to national clinical platform trial of COVID-19 community treatment	Use of Summary Care Record to enable near-real time recruitment of patients testing positive	Numbers of participants recruited to national trial
Cowan 2021(89)	Qualitative	Rapid prioritisation for rapid evaluation	Rapid topic prioritisation for rapid evaluation in adult social care	Adaptation of James Lind Alliance approach to rapid use	Rapid prioritisation of innovations for rapid evaluation; implications and necessary compromises
Crellin 2022(90)	Mixed methods	Rapid evaluation	Disparities of service research and patient engagement with home monitoring for COVID-19 services	Interviews and surveys with/of multiple stakeholders	Identification of strategies for inclusivity and reported disparities in experience and engagement with service
Dale 1996(23)	Qualitative	Rapid appraisal	Out of hours primary care strengths and weaknesses	Quantitative and qualitative survey methods	Availability of out of hours care and integration with other services
Farrer 2014(72)	Mixed methods	Rapid test changes	Changes in dietician assessments	Evaluation of ward data records	Use of new tool within designated time frame and accuracy of use
Fergus 2010(1)	Mixed methods	Rapid participatory appraisal	Palliative care out of hours provision	Routine data; semi-structured interviews; direct observations	Service provision and demand for services, barriers to accessing services
Gray 2017(91)	Mixed methods	Initial review	Rapid assessment of patients in acute medical unit	Staff feedback and information distribution	Triage of patients within timeframes, changes in times to assessment etc.

Green 2015(28)	Qualitative	Rapid appraisal	Reduced street lighting at night	Ethnographic data, household survey, document review	Public views on intervention
Guise 2018(79)	Qualitative	Rapid assessment	Remote hepatitis C testing	Focus groups and semi-structured interviews	Acceptability of approach, ease of use, other considerations including result management
Hamilton 1993(30)	Qualitative	Rapid appraisal	Planning for mental health provision	Semi-structured interviews	Service priorities for provision at borough level
Hanckel 2019(41)	Qualitative	Rapid ethnographic assessment	Daily mile in school	Interviews, observations, routine data	Implementation methods and perceived benefits
Joseph 2003(92)	Quantitative	Rapid reporting	Influenza vaccination	Data collection from records	Immunization uptake among population aged 65+
Lazenbatt 2001 (35)	Mixed methods	Rapid participatory appraisal	Women's access to health service provision	Overview of provision	Access to health services and related barriers
Lawlor 1999(38)	Qualitative	Rapid participatory appraisal	Sexual health needs in young people	Meta-planning	Needs in sexual health services planning
Lester 2002(93)	Mixed methods	Health inequality impact rapid appraisal	Impact of health inequalities on community and services	Complex multidisciplinary approaches	Cycle of deprivation – methods to break this
Newlands 2018(94)	Mixed methods	Rapid approach to knowledge translation	Quality improvement of pharmacy services	Nominal group technique, Delphi process	Priorities for knowledge translation into practice
Rowa-Dewar 2008(31)	Qualitative	Rapid appraisal approach	Public perceptions of cancer services	Multisite rapid appraisals in communities including those hard to reach	Views of cancer care in communities considered hard to reach

Supplementary Table 3: Primary studies in non-UK high income countries

Study ID	Country	Type of study	Rapid descriptor	Study question	Methods used	Data collected
Alguacil-Ramos 2019(95)	Spain	Quantitative	Rapid detection of safety signals	Adverse events of influenza vaccination	Population based register, real-time consultation	Specific adverse events, demographics impacted
Betsos 2021(50)	Canada	Qualitative	Rapid ethnographic study	Use of drug checking technologies	Fieldwork including interviews	Perceptions of efficacy
Bjornson-Benson 1993(96)	USA	Quantitative	Rapid feedback	Most effective trial recruitment methods	Real-time monitoring and rapid feedback on efficacy of different strategies	Proportion of participants recruited by each strategy
Boyd 2018(42)	Canada	Qualitative	Rapid ethnographic study	Supervised consumption sites for drug overdose preventions	Observations and interviews	Experiences of participants
Brown 2008(59)	USA	Mixed methods	Rapid assessment response and evaluation	Guiding interventions for urban health disparities	Mapping, observations, interviews, focus groups	Care received by key groups
Brown-Johnson 2019(97)	USA	Qualitative	Lightning report rapid qualitative synthesis	Barriers and facilitators of life-sustaining treatment decisions initiative	Interviews	Provision
Burks 2011(60)	USA	Qualitative	Rapid assessment	Barriers to prevention and treatment of HIV	Multiple triangulated methods with high user involvement	Barriers to accessing care

Cakir 2008(98)	Turkey	Quantitative	Rapid evaluation	Routine vaccination coverage	Survey, interviews	Vaccines received, reasons for non-vaccination
Campbell 2021(99)	Australia	Mixed methods	Emergency response priority research	Multiple projects	Infrastructure and processes including embedded researchers	Rapid evidence generation and translation
Carrillo 2022(100)	USA	Quantitative	Rapid acceleration	Standardisation of data collection for rapid COVID-19 research	Methods for standardisation, harmonisation and protection of data sharing	Success in improving data accessibility
CDC 2005(101)	USA	Quantitative	Rapid assessment	Vaccination coverage	Automated rapid analysis system	Adverse events, vaccination coverage, adherence to guidelines
Collins 2019(51)	Canada	Qualitative	Rapid ethnographic study	Impact of law enforcement on overdose preventions sites	Interviews and ethnographic fieldwork	Barriers to service access created by other services
Collins 2022(52)	USA	Qualitative	Rapid ethnographic study	COVID-19 public health measures impact on unstably housed drug users	Interviews and ethnographic fieldwork	Impact of public health measures on existing vulnerabilities
Corley 2021(102)	USA	Qualitative	Rapid research implementation	Impact of COVID-19 measures on clinical/community cohorts	Surveys	Mental health impact of COVID-19 associated measures on clinical and community cohorts
Currow 2012(103)	Australia & New Zealand	Quantitative	Rapid report	Metoclopramide	Data on use and symptoms in cohort	Benefit and adverse events, treatment adherence

D'Amore 2003(104)	USA	Quantitative	Brief report	Automatic therapeutic interchange programme	Pharmacy records review	Impact on outpatient therapy
Daviskiba 2021(105)	USA	Quantitative	Rapid assessment	Impact of COVID-19 on healthcare workers	Survey	Psychiatric symptoms, adequacy of PPE and training
Delaney 2019(106)	USA	Mixed methods	Rapid approach assessment	Low volume paediatric cardiac care programmes	Retrospective chart review	Quality of care metrics
Demeulemeester 2017(107)	Belgium	Quantitative	Rapid safety assessment	Seasonal influenza vaccination	Vaccination surveillance	Specified and unspecified adverse events
Douglas 2022(108)	USA	Quantitative	Rapid evaluation	Emerging therapies for pancreatic cancer	Platform trial	Efficacy and safety
Dudovitz 2021(109)	USA	Mixed methods	Rapid research	Research prioritisation for children and COVID-19	Rapid methods for research prioritisation – virtual meetings	Development of a research agenda to devise and test interventions
Dunbar 2018(110)	USA	Mixed methods	Brief research study	Connections to care programme	Interviews	effect on clients, government and community organisation spending
Dupont 2015(54)	Netherlands	Qualitative	Rapid assessment and response	Evidence based interventions for young people	Stepwise fieldwork	Use and patterns of use of cannabis
Eaves 2022(111)	USA	Qualitative	Rapid online ethnography (RARE)	Impact of COVID-19 policy on people who use drugs	Interviews and focus groups, observations	Perspectives and methodological challenges

Elsawy 2022(112)	Eastern Mediterranean countries	Quantitative	Rapid assessment	Impact of COVID-19 on mental health services	Survey of national mental health focal points	Impact of pandemic and responses to service restrictions
Elwy 2021(113)	USA	Qualitative	Rapid qualitative study	Vaccine hesitancy in COVID-19	Interviews and focus group	Views of vaccination, incidence of hesitancy
Foreman-Mackey 2019 (33)	Canada	Quantitative	Rapid evaluation	Overdose prevention sites	Semi-structured interviews	Experiences of people accessing smoking and injection services
Gale 2019(114)	USA	Qualitative	Rapid qualitative analysis	Evidence-based strategy in opioid prescribing	Interviews	Methods paper which focuses on comparison of rapid versus conventional qualitative analyses of interviews
Gagne 2013(115)	USA	Quantitative	Rapid assessment	Zoledronic acid adverse event	Analysis of data from commercial claims database	Myocardial infarctions in people taking zoledronic acid vs other bisphosphonates
Gawaya 2022(17)	Australia	Mixed methods	Rapid evaluation	Multiple programmes/practices	Team-based approach to rapid evaluation	Effectiveness and scalability
Goepp 2008(43)	USA	Qualitative	Rapid assessment	Barriers and facilitators to inpatient palliative care	Interviews	factors influencing service uptake
Gomez-Ramirez 2021(116)	Canada	Qualitative	Rapid qualitative assessment	Canada COVID-19 alert app	Walk-through of app; analysis of government documents and media coverage	Functionality, aims and purpose
Green 2013 (44)	USA	Qualitative	Rapid assessment	law enforcement overdose prevention	Interviews	Experiences of overdose prevention and naloxone

Hamarman 2014 (117)	USA	Qualitative	Rapid assessment	Sexually transmitted disease programmes	Interviews and observations	Service provision; integration and collaboration
Hasford 2020(118)	Germany	Quantitative	Rapid assessment	COVID-19 vaccines	Trial design	Large simple RCT benefits
Hensel 2016(119)	Canada	Mixed methods	Pragmatic randomised trial	Online platform for mental health self-management	Data analysis of trial outcomes; participant interviews	Recovery at 3 months
Im 2022(120)	USA	Qualitative	Rapid evaluation and appraisal	Impact of COVID-19 in refugee service provision and support	Interviews and focus groups, survey	Gaps in service provision and support
Keniston 2022(121)	USA	Qualitative	Rapid qualitative assessment	Inpatient surge planning and workforce deployment in COVID-19	Focus groups	Adaptations and associated issues in workforce planning and deployment
Kercsmar 2017 (71)	USA	Quantitative	Rapid evaluation	Paediatric asthma care redesign	Control charts (annotated)	Hospitalisations and ED visits
Khalid-Khan 2020(122)	Canada	Qualitative	Rapid-cycle program evaluation	Child and adolescent psychiatry programme	Focus groups and interviews	Identified improvements or refinements
Khubchandi 2021(123)	USA	Quantitative	Rapid national assessment	COVID-19 vaccine hesitancy	Survey	Likelihood of vaccination f
Kieber-Emmons 2018(2)	USA	Mixed methods	Rapid assessment	Care for cancer patients/survivors	Geographic information systems, observations, Interviews	Mortality rates, insurance coverage, provision of care, delivery methods
Kieber-Emmons 2022(78)	USA	Mixed methods	Focused rapid assessment process	Care for cancer patients/survivors	Geographic information systems, observations, Interviews	Modifiable factors in survivorship care

Kobau 2022(124)	USA	Qualitative	Rapid community assessment	Vaccine confidence in COVID-19	Interviews, listening sessions, observations, street intercept surveys	Barriers and facilitators to uptake
Kroeger 2013 (47)	USA	Mixed methods	Rapid ethnographic assessment	Sexual health services	Interviews	Views on available services/potential changes to services
Kuhlman 2021(125)	European Union	Mixed methods	Rapid assessment	Health workforce protection and preparedness in COVID-19	Assessment tool, secondary data sources, case studies	Actions taken and required, differences across areas of preparedness
Livorsi 2016 (126)	USA	Mixed methods	Rapid assessment	Barriers and facilitators to safety culture in an intensive care unit	Interviews	Reporting errors, approachability of authority figures and handovers.
Loosier 2020 (127)	USA	Qualitative	Rapid ethnographic assessment	Sexual health services	Interviews	Financial and care capability parameters
Maaskant 2021(128)	Netherlands	Qualitative	Rapid qualitative study	Isolation and families of hositalised COVID-19 patients	Patient record review and focus groups	Practices and nurse experiences
MacCarthy 2019 (129)	USA	Qualitative	Rapid assessment process	HIV prevention information	Interviews	HIV prevention information on mobile technology
McDonald 2022(130)	11 European countries	Quantitative	Rapid assessment	Take-home naloxone provision during COVID-19	Programme reporting instrument, survey	Capacity and adaptations
McNall 2004(131)	USA	Mixed methods	Rapid feedback evaluation	Retention in an HIV/AIDs intervention	Data collection and analysis of approaches	Retention rates and the factors impacting them

Minoyan 2022(132)	Canada	Mixed methods	Rapid assessment	Impact of COVID-19 health emergency declaration	Questionnaire and interviews	Access to health services, behaviour and experience
Moloney 2020(76)	USA	Qualitative	Rapid assessment procedure informed clinical ethnography case series	Clinical trial of interventions for traumatic injury conducted during COVID-19	Observations of multiple clinical situations and team processes	Integration of COVID-19 prevention strategies into clinical interventions in research context
Murray 2022(133)	Multinational	Quantitative	Rapid evaluation	Therapeutics for COVID-19 inpatients	Platform trial master protocol	Safety and efficacy
Murphy 2018 (32)	Georgia	Mixed methods	Rapid appraisal	Barriers to mental health services	Document review; semi-structured interviews, triangulation	Barriers to access for internally displaced populations
Nevedal 2021(134)	USA	Qualitative	Rapid qualitative analysis	Veterans Health Administration programme to identify and diffuse evidence-based interventions.	Interviews	Methods paper which focuses on comparison of rapid versus conventional qualitative analyses of interviews
Oh 2021(53)	USA	Qualitative	Rapid ethnographic assessment	Professional trearment and recovery resources for alcohol use	Observations and interviews	Perceptions of service use
Palinkas 2020(135)	USA	Qualitative	Rapid ethnographic assessment	Service delivery in acute care and trauma: impact of COVID-19	Observations by clinical participants, conversation summaries	Impacts on service delivery
Palinkas 2021a(136)	USA	Qualitative	Rapid assessment procedure informed clinical ethnography	Child and adolescent mental health practice: impact of COVID-19	Interviews, stakeholder contact	Impacts on service delivery and demand

Palinkas 2021b(137)	USA	Qualitative	Rapid assessment procedure informed clinical ethnography case series	Disaster preparedness: impact of COVID-19	Interviews, immersion techniques	Impacts on preparedness, strengths and capabilities, integrated responses
Palinkas 2022(77)	USA	Qualitative	Rapid assessment procedure informed clinical ethnography case series	Service delivery in acute care and trauma: impact of COVID-19	Observations by clinical participants, conversation summaries	Ongoing impacts and impact of service changes
Quintero Romero 2006 (46)	Italy	Mixed methods	Rapid ethnographic study	Information and support for breastfeeding	Interviews	Access to and need for support, experiences of services
Rains 2020(138)	USA	Mixed methods	Rapid cycle testing	Geriatric acute unplanned care	Routine data analysis and feedback	Unplanned healthcare episodes, costs, satisfaction
Renfro 2022(139)	USA	Qualitative	Rapid assessment procedure	Medication therapy management in primary care	Interviews	Implications for change from programme adoption
Rodriguez 2021(140)	USA	Mixed methods	Rapid evaluation	COVID-19 vaccination in ED for underserved patients	Survey	Vaccine hesitancy, vaccination availability, reasons for hesitancy
Rolf 2021(141)	Australia	Qualitative	Large-scale rapid analysis	Australian border and biosecurity policy in COVID-19	Survey and interviews	Success in using rapid methods
Romeu-Labayen 2022(142)	Spain	Qualitative	Rapid research evaluation and appraisal	PPE provision for nurses in COVID-19	Interviews	Experiences and perceptions
Rosteius 2022(143)	Netherlands	Qualitative	Rapid ethnographic research	Nursing home staff	Using existing relationships to enable rapid ethnography	Impact of long-term relationships on rapid work

Scott 2022(144)	USA	Mixed methods	Rapid assessment procedure informed clinical ethnography	Opioid treatment programmes	Interviews, surveys and site visits	Contextual factors impacting implementation of trial protocol
Seay 2017(145)	USA	Quantitative	Rapid assessment	Transgender experience and use of cervical screening	Survey	Uptake, causes of low uptake
Seay 2015(146)	USA	Quantitative	Rapid assessment	cancer screening in immigrant communities	Survey	Uptake and access disparities
Shaw 2022(147)	USA	Qualitative	Rapid research and evaluation methods	Telehealth in patients following sexual assault	Interviews	Experience including successes and challenges
Shimkhada 2021(148)	USA	Qualitative	Rapid assessment	Support in metastatic breast cancer	Twitter chat	Barriers to care and support
Short 2022(149)	USA	Quantitative	Rapid parallel evaluation	Relapsed/refractory acute myeloid leukaemia	Platform trial (phase II)	Efficacy
Skillman 2018(68)	USA	Mixed methods	Rapid cycle evaluation	Payment and service models for Medicare/Medicaid	Interviews and focus groups, site visits	Cost, quality, use and experiences
Solomon 2006(150)	USA	Qualitative	Rapid assessment procedure	HIV prevention programming in Community Mental Health Centre	Interviews, focus groups, ecological assessment	Availability and perception of availability of services and materials
Sperber 2019(62)	USA	Qualitative	Rapid qualitative assessment	Walking programme for hospitalised older adults	interview, document review	Impacts of early stage implementation

Srinivasan 2020(151)	USA	Qualitative	Rapid qualitative analytic approach	Transition to video call primary care appointments	Interviews	Critical issues to enable sustainability of change
Talati 2021(152)	USA	Quantitative	Rapid assessment	Telehealth for maternity care	Survey	Satisfaction
Talwai 2021(153)	USA	Quantitative	Rapid research	Multiple COVID-19 projects	Data repository	Data usage in research
Tan 2021(154)	Singapore	Qualitative	Rapid qualitative research	Health and social care need in COVID-19 public health measures context	Interviews, expanded field notes	Sexual health service provision and need
Taubenberger 2021(155)	USA	Qualitative	Rapid cycle assessment	Services for opioid use and overdose prevention	Interviews and observations	Knowledge and perception of services
Tort-Nasarre 2021(156)	Spain	Qualitative	Rapid research evaluation and appraisal	Nurse response to organisational changes in COVID-19	Interviews	Responses to change
Toth 2021(157)	Hungary	Quantitative	Rapid assessment of avoidable blindness	Avoidable blindness costs in diabetic retinopathy	Derived costs of treatment and prevalent illness data	Costs
Valderrama 2006 (61)	Spain	Qualitative	Rapid assessment	Drug abuse prevention needs of young people	Interviews (from rapid assessment and response evaluation methods)	Coverage of mandated prevention needs
Vazquez 2022(158)	USA	Qualitative	Rapid qualitative study	Risk-reduction measures for COVID-19 in institutions	Focus groups	Perceptions of stakeholders
Wesolowski 2020(159)	USA	Quantitative	Rapid assessments	Adoption of non-pharmaceutical interventions for COVID-19	Online panel	Use of non-pharmaceutical interventions, positive test results

Wichmann 2010 (160)	Germany	Quantitative	Rapid assessment	Pandemic influenza vaccination effectiveness	Data records of influenza cases vaccination status	Effectiveness estimates – breakthrough infections
Williams 2019 (161)	USA	Mixed methods	Rapid gap assessment	Sexual health services coverage	Site visits, checklists, online data sources, open-ended questioning	Service provision in urgent care centres; challenges experienced
Wiss 2019 (162)	USA	Quantitative	Rapid assessment	nutritional services in drugs misuse treatment services	Survey	Service provision and characteristics
Xiang 2021(163)	USA	Quantitative	Streamlined eligibility assessment	Recruitment for hematology and oncology research studies	Implementation of standardised prescreening protocol	Enrolment to clinical trials
Zakocs 2015(69)	USA	Mixed methods	Rapid feedback cycle	Primary prevention programs for domestic violence	Use of data-to-action framework	Impact of methods; capacity building

Supplementary Table 4: Studies assessing implementation in high income countries

Study ID	Country	Type of study	Rapid descriptor	Study question	Results
Agaku 2022(164)	USA	Quantitative	Rapid evaluation	Federal and state tobacco law	Access and equity of impact
Ash 2008 (165)	USA	Mixed methods	Rapid assessment process	Informatics in clinical decision support	implementation for rapid assessment process and actionable feedback
Belford 2017 (166)	UK	Mixed methods	Evaluability assessment giving quick feedback	Community development programme to reduce health inequality	Stakeholder involvement to produce logic model of programme theory and evaluation opinions
Cohn 2021(65)	USA	Qualitative	Rapid evaluation using consolidated framework for implementation research	Mobile health use in HIV care settings	Identification of barriers and facilitators, both general and intervention specific.
Haigh 2013 (167)	Australia and New Zealand	Quantitative	Rapid health impact assessment	Use of health impact reports including rapid assessments	Quality assessment of reports to develop improvements in practice
Hailey 2001 (168)	Canada	Quantitative	Rapid assessment process	Rapid HTAs	Rapid HTA programme in provincial healthcare system in response to urgent requests for advice
Holdsworth 2020 (8)	USA	Mixed methods	Rapid assessment	ICU redesign for patient safety	Implementation methods and perceived benefits; barriers and facilitators
Keith 2017 (63)	USA	Qualitative	Rapid cycle evaluation	Barriers and facilitators to different comprehensive primary care initiative components	Consolidated framework for implementation research useful for rapid cycle

					evaluation of practice transformation initiatives
Last 2021(66)	USA	Mixed methods	Rapid participatory methods, rapid prototyping	Depression screening in primary care	Engagement of diverse stakeholders, production of acceptable and feasible implementation strategy
Lewinski 2021(169)	USA	Qualitative	Rapid qualitative analysis	Care coordination in rural areas	Informing near-real time implementation of systems is possible using these methods
Li 2021(170)	USA	Quantitative	Rapid assessment	Assessment of COVID-19 vaccination implementation	Event study of phased implementation process informs policy
Ohrling 2020(67)	Sweden	Mixed methods	Rapid implementation action research case study	Emergency response to COVID-19	Changes in health -care services and success of implementaiton
Sperber 2019 (62)	USA	Qualitative	Rapid qualitative assessment	Early strategies to implement evidence-based walking program for hospitalized older adults	Lessons from early stage implementation
Wright 2015 (3)	USA	Qualitative	Rapid assessment process	Best practice for a clinical decision support system	Challenges and lessons for implementation

References for supplementary material

1. Fergus CJ, Chinn DJ, Murray SA. Assessing and improving out-of-hours palliative care in a deprived community: a rapid appraisal study. Palliative Medicine. 2010;24(5):493-500.
2. Kieber-Emmons A, Crabtree BF, Miller W. A new mixed methods approach to uncover multi-level barriers and facilitators of cancer survivorship. Journal of Clinical Oncology Conference. 2018;36(7).
3. Wright A, Sittig DF, Ash JS, Erickson JL, Hickman TT, Paterno M, et al. Lessons learned from implementing service-oriented clinical decision support at four sites: A qualitative study. International Journal of Medical Informatics. 2015;84(11):901-11.
4. Manderson L, Aaby P. An epidemic in the field? Rapid assessment procedures and health research. Social Science and Medicine. 1992;35(7):839-50.
5. McMullen CK, Ash JS, Sittig DF, Bunce A, Guappone K, Dykstra R, et al. Rapid assessment of clinical information systems in the healthcare setting: an efficient method for time-pressed evaluation. Methods of information in medicine. 2011;50(4):299-307.
6. Needle RH, Trotter IRT, Goosby E, Bates C, Von Zinkernagel D. Methodologically sound rapid assessment and response: Providing timely data for policy development on drug use interventions and HIV prevention. International Journal of Drug Policy. 2000;11(1-2):19-23.
7. Needle RH, Trotter RT, 2nd, Singer M, Bates C, Page JB, Metzger D, et al. Rapid assessment of the HIV/AIDS crisis in racial and ethnic minority communities: an approach for timely community interventions. American Journal of Public Health. 2003;93(6):970-9.
8. Holdsworth LM, Safaeinili N, Winget M, Lorenz KA, Lough M, Asch S, et al. Adapting rapid assessment procedures for implementation research using a team-based approach to analysis: a case example of patient quality and safety interventions in the ICU. Implementation Science. 2020;15(1):12.
9. Peiro R. Rapid appraisal methodology for 'health for all' policy formulation analysis. 2003.
10. Peiro R, Alvarez-Dardet C, Plasencia A, Borrell C, Colomer C, Moya C, et al. Rapid appraisal methodology for 'health for all' policy formulation analysis. Health Policy. 2002;62(3):309-28.
11. Fitch C, Rhodes T, Stimson GV. Origins of an epidemic: The methodological and political emergence of rapid assessment. International Journal of Drug Policy. 2000;11(1-2):63-82.
12. Fitch C, Stimson GV, Rhodes T, Poznyak V. Rapid assessment: an international review of diffusion, practice and outcomes in the substance use field. Soc Sci Med. 2004;59(9):1819-30.
13. Stimson GV, Fitch C, DesJarlais D, Poznyak V, Perlis T, Oppenheimer E, et al. Rapid assessment and response studies of injection drug use: knowledge gain, capacity building, and intervention development in a multisite study. American Journal of Public Health. 2006;96(2):288-95.
14. Walton H, Vindrola-Padros C, Crellin NE, Sidhu MS, Herlitz L, Litchfield I, et al. Patients' experiences of, and engagement with, remote home monitoring services for COVID-19 patients: A rapid mixed-methods study. Health Expectations.n/a(n/a).
15. Humphries K, Maxwell S, Bartholomew S, Clutterbuck D. Rapid service evaluation of in-house blood self-sampling for interval STI testing for PrEP cohort at minimal cost. HIV Medicine. 2021;22(SUPPL 2):82-3.
16. Vindrola-Padros C, Ledger J, Hill M, Tomini S, Spencer J, Fulop NJ. The Special Measures for Quality and Challenged Provider Regimes in the English NHS: A Rapid Evaluation of a National Improvement Initiative for Failing Healthcare Organisations. International Journal of Health Policy & Management. 2022;27:27.
17. Gawaya M, Terrill D, Williams E. Using rapid evaluation methods to assess service delivery changes: Lessons learned for evaluation practice during the COVID-19 pandemic. Evaluation Journal of Australasia. 2022;22(1):30-48.
18. Sidhu M, Pollard J, Sussex J. Vertical integration of primary care practices with acute hospitals in England and Wales: why, how and so what? Findings from a qualitative, rapid evaluation. BMJ Open. 2022;12(1):e053222.

19. Parkinson S, Smith J, Sidhu M. Early development of primary care networks in the NHS in England: A qualitative mixed-methods evaluation. BMJ Open. 2021;11(12) (no pagination)(e055199).
20. Watson D, Baralle NL, Alagil J, Anil K, Ciccognani S, Dewar-Haggart R, et al. How do we engage people in testing for COVID-19? A rapid qualitative evaluation of a testing programme in schools, GP surgeries and a university. BMC Public Health. 2022;22(1):305.
21. Rhodes T, Stimson GV, Fitch C, Ball A, Renton A. Rapid assessment, injecting drug use, and public health. The Lancet. 1999;354(9172):65-8.
22. Campbell J. A critical appraisal of participatory methods in development research. International Journal of Social Research Methodology. 2002;5(1):19-29.
23. Dale J, Shipman C, Lacock L, Davies M. Creating a shared vision of out of hours care: using rapid appraisal methods to create an interagency, community oriented, approach to service development. Bmj. 1996;312(7040):1206-10.
24. Murray SA. Listening to local voices : adapting rapid appraisal to assess health and social needs in general practice. 1994.
25. Murray SA. Experiences with "rapid appraisal" in primary care: involving the public in assessing health needs, orientating staff, and educating medical students. Bmj. 1999;318(7181):440-4.
26. Harvey HD, Fleming P. A rapid appraisal method for the selection and pre-testing of environmental health leaflets. Journal of The Royal Society for the Promotion of Health. 2000;120(2):112-6.
27. Harvey HD, Fleming P, Patterson M. A rapid appraisal method for reviewing the effectiveness of workplace smoking policies in large and medium sized organisations. Journal of The Royal Society for the Promotion of Health. 2001;121(1):50-5.
28. Green J, Perkins C, Steinbach R, Edwards P. Reduced street lighting at night and health: A rapid appraisal of public views in England and Wales. Health Place. 2015;34:171-80.
29. Beebe J, Given L. Rapid assessment process. The sage encyclopedia of qualitative research methods

Thousand Oaks, California: SAGE Publications; 2012.
30. Hamilton S. Felt needs assessment of mental health (rapid service appraisal): Barking and Havering. (Public health research report, ISSN 09689818 ; no 11). 1993:31.
31. Rowa-Dewar N, Ager W, Ryan K, Hargan I, Hubbard G, Kearney N. Using a rapid appraisal approach in a nationwide, multisite public involvement study in Scotland. Qualitative Health Research. 2008;18(6):863-9.
32. Murphy A, Chikovani I, Uchaneishvili M, Makhashvili N, Roberts B. Barriers to mental health care utilization among internally displaced persons in the republic of Georgia: a rapid appraisal study. BMC health services research. 2018;18(1):306.
33. Foreman-Mackey A, Bayoumi AM, Miskovic M, Kolla G, Strike C. 'It's our safe sanctuary': Experiences of using an unsanctioned overdose prevention site in Toronto, Ontario. International Journal of Drug Policy. 2019;73:135-40.
34. Beebe J. Rapid assessment and response: Sound methodology for producing timely responses. International Journal of Drug Policy. 2000;11(1-2):29-31.
35. Lazenbatt A, Lynch U, O'Neill E. Revealing the hidden 'troubles' in Northern Ireland: The role of participatory rapid appraisal. Health Education Research. 2001;16(5):567-78.
36. Brown CS, Lloyd S, Murray SA. Using consecutive Rapid Participatory Appraisal studies to assess, facilitate and evaluate health and social change in community settings. BMC Public Health. 2006;6 (no pagination)(68).
37. Annett H. Guidelines for rapid participatory appraisals to assess community health needs: a focus on health improvements for low-income urban and rural areas. Geneva: Division of Strengthening of Health Services World Health Organization available from HMSO; 1995. iv,60 p.
38. Lawlor D. Rapid participatory appraisal of young people's sexual health needs: an evaluation of meta-planning. 1999.

39. Desai A, Donetto S. Evolving ethnographic sensibilities: Using actor-network theory in health services research. BMJ Open. 2019;9 (Supplement 1):A5.
40. Vindrola-Padros C, Vindrola-Padros B. Quick and dirty? A systematic review of the use of rapid ethnographies in healthcare organisation and delivery. BMJ Qual Saf. 2018;27(4):321-30.
41. Hanckel B, Ruta D, Scott G, Peacock JL, Green J. The Daily Mile as a public health intervention: a rapid ethnographic assessment of uptake and implementation in South London, UK. BMC Public Health. 2019;19(1):1167.
42. Boyd J, Collins AB, Mayer S, Maher L, Kerr T, McNeil R. Gendered violence and overdose prevention sites: a rapid ethnographic study during an overdose epidemic in Vancouver, Canada. Addiction. 2018;113(12):2261-70.
43. Goepp JG, Meykler S, Mooney NE, Lyon C, Raso R, Julliard K. Provider insights about palliative care barriers and facilitators: results of a rapid ethnographic assessment. Am J Hosp Palliat Care. 2008;25(4):309-14.
44. Green TC, Zaller N, Palacios WR, Bowman SE, Ray M, Heimer R, et al. Law enforcement attitudes toward overdose prevention and response. Drug Alcohol Depend. 2013;133(2):677-84.
45. Ackerman S, Sarkar U, Tieu L, Hamdley M, Schillinger D, Jahn K, et al. Meaningful use in the safety net: A rapid ethnography of patient portal implementation at five community health centers in California. Journal of the American Medical Informatics Association. 2017;24(5):903-12.
46. Quintero Romero S, Bernal R, Barbiero C, Passamonte R, Cattaneo A. A rapid ethnographic study of breastfeeding in the North and South of Italy. Int Breastfeed J. 2006;1:14.
47. Kroeger K, Willilams S, Alexander-Pender C, Ford J, Vasani V. "Just one thing on the plate" - Views of community members and providers on sexual health services in the fort mcpherson area of Southwest Atlanta: Findings from a rapid ethnographic assessment. Sexually Transmitted Infections Conference: STI and AIDS World Congress. 2013;89(SUPPL. 1).
48. Ayele R, Manges KA, Leonard C, Lee M, Galenbeck E, Molla M, et al. How Context Influences Hospital Readmissions from Skilled Nursing Facilities: A Rapid Ethnographic Study. Journal of the American Medical Directors Association. 2021;22(6):1248-54.e3.
49. Manges KA, Ayele R, Leonard C, Lee M, Galenbeck E, Burke RE. Differences in transitional care processes among high-performing and low-performing hospital-SNF pairs: a rapid ethnographic approach. BMJ Quality & Safety. 2021;30(8):648-57.
50. Betsos A, Valleriani J, Boyd J, Bardwell G, Kerr T, McNeil R. "I couldn't live with killing one of my friends or anybody": A rapid ethnographic study of drug sellers' use of drug checking. International Journal of Drug Policy. 2021;87:102845.
51. Collins AB, Boyd J, Mayer S, Fowler A, Kennedy MC, Bluthenthal RN, et al. Policing space in the overdose crisis: A rapid ethnographic study of the impact of law enforcement practices on the effectiveness of overdose prevention sites. International Journal of Drug Policy. 2019;73:199-207.
52. Collins AB, Edwards S, McNeil R, Goldman J, Hallowell BD, Scagos RP, et al. A rapid ethnographic study of risk negotiation during the COVID-19 pandemic among unstably housed people who use drugs in Rhode Island. International Journal of Drug Policy. 2022;103:103626.
53. Oh H, Yamada AM. Exploring second generation Korean American alcohol use through church-based participatory research: A rapid ethnographic assessment in Los Angeles, California, United States. Health & Social Care in the Community. 2021;29(5):1359-67.
54. Dupont HB, Kaplan CD, Braam RV, Verbraeck HT, de Vries NK. The application of the rapid assessment and response methodology for cannabis prevention research among youth in the Netherlands. International Journal of Drug Policy. 2015;26(8):731-8.
55. Ong BN. Rapid appraisal and health policy. 1996.
56. Ong BN, Humphris G, Annett H, Rifkin S. Rapid appraisal in an urban setting, an example from the developed world. Soc Sci Med. 1991;32(8):909-15.
57. Organization WH, UNAIDS. SEX-RAR Guide. https://apps.who.int/iris/bitstream/handle/10665/42681/9241545585.pdf?sequence=1&isAllowed=y2002.

58. Trotter IRT, Needle R, Goosby E, Bates C, Singer M. A Methodological Model for Rapid Assessment, Response, and Evaluation: The RARE Program in Public Health. Field Methods. 2001;13(2):137-59.
59. Brown DR, Hernandez A, Saint-Jean G, Evans S, Tafari I, Brewster LG, et al. A participatory action research pilot study of urban health disparities using rapid assessment response and evaluation. American Journal of Public Health. 2008;98(1):28-38.
60. Burks DJ, Robbins R, Durtschi JP. American Indian gay, bisexual and two-spirit men: a rapid assessment of HIV/AIDS risk factors, barriers to prevention and culturally-sensitive intervention. Culture, health & sexuality. 2011;13(3):283-98.
61. Valderrama JC, Tortajada S, Alapont L, Vidal A, Perez Ma J, Castellano M, et al. Rapid assessment of drug abuse prevention needs for youth in small Spanish municipalities: Coping with resource limitations. Journal of Drug Issues. 2006;36(1):29-52.
62. Sperber NR, Bruening RA, Choate A, Mahanna E, Wang V, Powell BJ, et al. Implementing a Mandated Program Across a Regional Health Care System: A Rapid Qualitative Assessment to Evaluate Early Implementation Strategies. Quality Management in Health Care. 2019;28(3):147-54.
63. Keith RE, Crosson JC, O'Malley AS, Cromp D, Taylor EF. Using the Consolidated Framework for Implementation Research (CFIR) to produce actionable findings: a rapid-cycle evaluation approach to improving implementation. Implement Sci. 2017;12(1):15.
64. Shrank WH. The Center For Medicare And Medicaid Innovation's blueprint for rapid-cycle evaluation of new care and payment models. Health Affairs (Millwood). 2013;32(4):807-12.
65. Cohn WF, Canan CE, Knight S, Waldman AL, Dillingham R, Ingersoll K, et al. An Implementation Strategy to Expand Mobile Health Use in HIV Care Settings: Rapid Evaluation Study Using the Consolidated Framework for Implementation Research. JMIR MHealth and UHealth. 2021;9(4):e19163.
66. Last BS, Buttenheim AM, Futterer AC, Livesey C, Jaeger J, Stewart RE, et al. A pilot study of participatory and rapid implementation approaches to increase depression screening in primary care. BMC Family Practice. 2021;22(1):228.
67. Ohrling M, Ovretveit J, Lockowandt U, Brommels M, Sparring V. Management of the emergency response to the SARS-CoV-2 (COVID-19) outbreak in Stockholm, Sweden, and winter preparations. Journal of Primary Health Care. 2020;12(3):207-14.
68. Skillman M, Cross-Barnet C, Friedman Singer R, Rotondo C, Ruiz S, Moiduddin A. A Framework for Rigorous Qualitative Research as a Component of Mixed Method Rapid-Cycle Evaluation. Qualitative Health Research. 2018;29(2):279-89.
69. Zakocs R, Hill JA, Brown P, Wheaton J, Freire KE. The Data-to-Action Framework: A Rapid Program Improvement Process. Health Educ Behav. 2015;42(4):471-9.
70. Fergus CJY. Assessing and improving out-of-hours palliative care in a deprived community: a rapid appraisal study. 2010.
71. Kercsmar CM, Beck AF, Sauers-Ford H, Simmons J, Wiener B, Crosby L, et al. Association of an asthma improvement collaborative with health care utilization in medicaid-insured pediatric patients in an urban community. JAMA Pediatrics. 2017;171(11):1072-80.
72. Farrer K, Donaldson E, Blackett B, Lloyd H, Forde C, Melia D, et al. Nutritional screening of elderly patients: a health improvement approach to practice. J Hum Nutr Diet. 2014;27(2):184-91.
73. Schneeweiss S, Shrank WH, Ruhl M, Maclure M. Decision-making aligned with rapid-cycle evaluation in health care. International Journal of Technology Assessment in Health Care. 2015;31(4):214-22.
74. Palinkas LA, Mendon SJ, Hamilton AB. Innovations in Mixed Methods Evaluations. Annual Review of Public Health. 2019;40:423-42.
75. Palinkas LA, Zatzick D. Rapid Assessment Procedure Informed Clinical Ethnography (RAPICE) in Pragmatic Clinical Trials of Mental Health Services Implementation: Methods and Applied Case Study. Administration & Policy in Mental Health. 2019;46(2):255-70.

76. Moloney K, Scheuer H, Engstrom A, Schreiber M, Whiteside L, Nehra D, et al. Experiences and Insights from the Early US COVID-19 Epicenter: A Rapid Assessment Procedure Informed Clinical Ethnography Case Series. Psychiatry. 2020;83(2):115-27.
77. Palinkas LA, Engstrom A, Whiteside L, Moloney K, Zatzick D. A Rapid Ethnographic Assessment of the Impact of the COVID-19 Pandemic on Mental Health Services Delivery in an Acute Care Medical Emergency Department and Trauma Center. Administration & Policy in Mental Health. 2022;49(2):157-67.
78. Kieber-Emmons AM, Miller WL, Rubinstein EB, Howard J, Tsui J, Rankin JL, et al. A Novel Mixed Methods Approach Combining Geospatial Mapping and Qualitative Inquiry to Identify Multilevel Policy Targets: The Focused Rapid Assessment Process (fRAP) Applied to Cancer Survivorship. Journal of Mixed Methods Research. 2022;16(2):183-206.
79. Guise A, Witzel TC, Mandal S, Sabin C, Rhodes T, Nardone A, et al. A qualitative assessment of the acceptability of hepatitis C remote self-testing and self-sampling amongst people who use drugs in London, UK. BMC Infectious Diseases. 2018;18 (1) (no pagination)(281).
80. Phillips CB, Dwan K, Hepworth J, Pearce C, Hall S. Using qualitative mixed methods to study small health care organizations while maximising trustworthiness and authenticity. BMC Health Services Research. 2014;14:559.
81. Williams SP, Kinsey J, Carry MG, Terry L, Wells J, Kroeger K. Get In, Get Tested, Get Care: STD Services in Urban Urgent Care Centers. Sexually Transmitted Diseases. 2019;46(10):648-53.
82. Dunet DO, Sparling PB, Hersey J, Williams-Piehota P, Hill MD, Hanssen C, et al. A new evaluation tool to obtain practice-based evidence of worksite health promotion programs. Preventing chronic disease. 2008;5(4):A118.
83. Gray TA, Dumville JC, Christie J, Cullum NA. Rapid research and implementation priority setting for wound care uncertainties. PLoS ONE. 2017;12(12):e0188958.
84. Aspray TJ, Nesbit K, Cassidy TP, Hawthorne G. Rapid assessment methods used for health-equity audit: Diabetes mellitus among frail British care-home residents. Public Health. 2006;120(11):1042-51.
85. Banga S, Chabanon AL, Eymin C, Caroe T, Butler K, Moureau A. Near real-time safety surveillance of three seasonal influenza vaccines in Europe during influenza season 2017/18. Pharmacoepidemiology and Drug Safety. 2018;27 (Supplement 2):396.
86. Begg NT, Gill ON, White JM. COVER (Cover Of Vaccination Evaluated Rapidly): Description of the England and Wales scheme. Public Health. 1989;103(2):81-9.
87. Brook MG, Rusere L, Coppin-Browne L, McDonagh S, McSorley J. A prospective study of the effectiveness of electronic patient records in rapid-cycle assessment of treatment and partner notification outcomes for patients with genital chlamydia and gonorrhoea infection. Sexually Transmitted Infections. 2011;87(2):152-5.
88. Cake C, Ogburn E, Pinches H, Coleman G, Seymour D, Woodard F, et al. Development and evaluation of rapid data-enabled access to routine clinical information to enhance early recruitment to the national clinical platform trial of COVID-19 community treatments. Trials [Electronic Resource]. 2022;23(1):62.
89. Cowan K, Fulop NJ, Harshfield A, Ng PL, Ntouva A, Sidhu M, et al. Rapid prioritisation of topics for rapid evaluation: the case of innovations in adult social care and social work. Health Research Policy & Systems. 2021;19(1):34.
90. Crellin NE, Herlitz L, Sidhu MS, Ellins J, Georghiou T, Litchfield I, et al. Examining disparities relating to service reach and patient engagement with COVID-19 remote home monitoring services in England: a mixed methods rapid evaluation. medRxiv. 2022;22.
91. Gray A, Baker K. Assessment for change. Acute Medicine. 2017;16 (3):127-8.
92. Joseph C, Goddard N. Influenza vaccine uptake in the elderly: Results from a rapid assessment of the effectiveness of new government policy in England for the winters 2000/2001 and 2001/2002. Vaccine. 2003;21(11-12):1137-48.
93. Lester C. Priority setting with Health Inequality Impact Assessment. 2002.

94. Newlands RS, Power A, Young L, Watson M. Quality improvement of community pharmacy services: a prioritisation exercise. International Journal of Pharmacy Practice. 2018;26(1):39-48.
95. Alguacil-Ramos AM, Portero-Alonso A, Pastor-Villalba E, Muelas-Tirado J, Diez-Domingo J, Sanchis-Ferrer A, et al. Rapid assessment of enhanced safety surveillance for influenza vaccine. Public Health. 2019;168:137-41.
96. Bjornson-Benson WM, Stibolt TB, Manske KA, Zavela KJ, Youtsey DJ, Sonia Buist A. Monitoring recruitment effectiveness and cost in a clinical trial. Controlled Clinical Trials. 1993;14(2, Supplement):52-67.
97. Brown-Johnson C, Lo N, Giannitrapani K, Lowery J, Foglia MB, Walling A, et al. A Rapid Qualitative Synthesis of Insights from the Veterans Administration Life-Sustaining Treatment Decisions Initiative (LSTDI) National Implementation (QI703). Journal of Pain and Symptom Management. 2019;57 (2):457.
98. Cakir B, Uner S, Temel F, Akin L. Lot quality survey: An appealing method for rapid evaluation of vaccine coverage in developing countries - Experience in Turkey. BMC Public Health. 2008;8 (no pagination)(240).
99. Campbell D, Edwards B, Milat A, Thackway S, Whittaker E, Goudswaard L, et al. NSW Health COVID-19 Emergency Response Priority Research program: a case study of rapid translation of research into health decision making. Public Health Research & Practice. 2021;31(4):10.
100. Carrillo GA, Cohen-Wolkowiez M, D'Agostino EM, Marsolo K, Wruck LM, Johnson L, et al. Standardizing, harmonizing, and protecting data collection to broaden the impact of COVID-19 research: the rapid acceleration of diagnostics-underserved populations (RADx-UP) initiative. Journal of the American Medical Informatics Association. 2022;29(9):1480-8.
101. Centers for Disease C, Prevention. Rapid assessment of influenza vaccination coverage among HMO members--northern California influenza seasons, 2001-02 through 2004-05. MMWR Morb Mortal Wkly Rep. 2005;54(27):676-8.
102. Corley SS, Gillezeau C, Molina L, Alpert N, Eugene A, Lieberman-Cribbin W, et al. Using Rapid Research Implementation and Collaborations to Assess the Mental Health Impact of the COVID-19 Pandemic Among Community and Clinical Cohorts. Disaster Medicine & Public Health Preparedness. 2021:1-5.
103. Currow DC, Vella-Brincat J, Fazekas B, Clark K, Doogue M, Rowett D. Pharmacovigilance in hospice/palliative care: Rapid report of net clinical effect of metoclopramide. Journal of Palliative Medicine. 2012;15(10):1071-5.
104. D'Amore M, Masters P, Maroun C. Impact of an automatic therapeutic interchange program on discharge medication selection. Hospital Pharmacy. 2003;38(10):942-6.
105. Daviskiba SE, MacKenzie MA, Dow M, Johnston P, Balon R, Javanbakht A, et al. Rapid assessment of mental health of Detroit-area health care workers during the COVID-19 pandemic. Annals of Clinical Psychiatry. 2021;33(2):101-7.
106. Delaney AE, Dadlez NM, Marshall AC. Alternative approach to pediatric cardiac quality assessment for low-volume centers. Congenital Heart Disease. 2019;14(4):665-70.
107. Demeulemeester M, Lavis N, Balthazar Y, Lechien P, Heijmans S. Rapid safety assessment of a seasonal intradermal trivalent influenza vaccine. Hum Vaccin Immunother. 2017;13(4):889-94.
108. Douglas JE, Liu S, Ma J, Wolff RA, Pant S, Maitra A, et al. PIONEER-Panc: a platform trial for phase II randomized investigations of new and emerging therapies for localized pancreatic cancer. BMC Cancer. 2022;22(1):14.
109. Dudovitz RN, Russ S, Berghaus M, Iruka IU, DiBari J, Foney DM, et al. COVID-19 and Children's Well-Being: A Rapid Research Agenda. Maternal & Child Health Journal. 2021;25(11):1655-69.
110. Dunbar MS, Towe VL, Ayer L, Martineau M. Connections to Care (C2C): The Perspectives of Leaders at Community-Based Organizations That Are Integrating Mental Health Supports. Rand health q. 2018;7(4):7.

111. Eaves ER, Trotter RT, 2nd, Marquez B, Negron K, Doerry E, Mensah D, et al. Possibilities and constraints of rapid online ethnography: Lessons from a rapid assessment of COVID-19 policy for people who use drugs. Frontiers in Sociology. 2022;7:959642.
112. Elsawy W, Fouad H, Saeed K. Impact of COVID-19 on mental health and psychosocial support services in the Eastern Mediterranean Region - results of a rapid assessment. Eastern Mediterranean Health Journal. 2022;28(5):321-8.
113. Elwy AR, Clayman ML, LoBrutto L, Miano D, Ann Petrakis B, Javier S, et al. Vaccine hesitancy as an opportunity for engagement: A rapid qualitative study of patients and employees in the U.S. Veterans Affairs healthcare system. Vaccine: X. 2021;9:100116.
114. Gale RC, Wu J, Erhardt T, Bounthavong M, Reardon CM, Damschroder LJ, et al. Comparison of rapid vs in-depth qualitative analytic methods from a process evaluation of academic detailing in the Veterans Health Administration. Implementation Science. 2019;14(1):11.
115. Gagne JJ, Wang S, Schneeweiss S. A multivariable-adjusted rapid assessment of the association between zoledronic acid and myocardial infarction. Pharmacoepidemiology and Drug Safety. 2013;1):378.
116. Gomez-Ramirez O, Medeiros P, Wainer R, Iyamu I. Does the 'canada COVID-19 alert' app stand up to critical scrutiny? a rapid qualitative assessment. BMJ Open. 2021;11(SUPPL 1):A5.
117. Hamarman A, Mason P, Kroeger K, Alexander-Pender C. The impact of budget cuts among local STD programs in New Jersey: Results of a rapid ethnographic assessment. Sexually Transmitted Diseases. 2014;1):S93.
118. Hasford J. Large Simple Double-Blind Randomized Trials for the Rapid Assessment of the Effectiveness of COVID-19 Vaccines. Journal of Infectious Diseases. 2020;222(9):1571-2.
119. Hensel JM, Shaw J, Jeffs L, Ivers NM, Desveaux L, Cohen A, et al. A pragmatic randomized control trial and realist evaluation on the implementation and effectiveness of an internet application to support self-management among individuals seeking specialized mental health care: A study protocol. BMC Psychiatry. 2016;16 (1) (no pagination)(350).
120. Im H, George N. Impacts of COVID-19 on Refugee Service Provision and Community Support: A Rapid Assessment during the Pandemic. Social Work in Public Health. 2022;37(1):84-103.
121. Keniston A, Sakumoto M, Astik GJ, Auerbach A, Eid SM, Kangelaris KN, et al. Adaptability on Shifting Ground: a Rapid Qualitative Assessment of Multi-institutional Inpatient Surge Planning and Workforce Deployment During the COVID-19 Pandemic. Journal of General Internal Medicine. 2022;22:22.
122. Khalid-Khan S, Braund H, Dare J, Turnridge J, Dalgarno N. Evaluating Child and Adolescent Psychiatry sub-specialty program using rapid-cycle approach. Journal of the Canadian Academy of Child and Adolescent Psychiatry. 2020;29(4):263.
123. Khubchandani J, Sharma S, Price JH, Wiblishauser MJ, Sharma M, Webb FJ. COVID-19 Vaccination Hesitancy in the United States: A Rapid National Assessment. Journal of Community Health. 2021;46(2):270-7.
124. Kobau R, Carry M, Rubenstein BL, Denson D, Uribe C, Zajac J, et al. Implementing the COVID-19 Rapid Community Assessment on Vaccine Confidence: Lessons Learned From Alabama and Georgia. Public Health Reports. 2022;137(5):832-40.
125. Kuhlmann E, Brinzac MG, Burau V, Correia T, Ungureanu MI. Health workforce protection and preparedness during the COVID-19 pandemic: a tool for the rapid assessment of EU health systems. European Journal of Public Health. 2021;31(Supplement_4):iv14-iv20.
126. Livorsi D, Knobloch MJ, Blue LA, Swafford K, Maze L, Riggins K, et al. A rapid assessment of barriers and facilitators to safety culture in an intensive care unit. Int Nurs Rev. 2016;63(3):372-6.
127. Loosier PS, Carter MW, Hsu KK, Doshi S, Peterson Maddox BL, Kroeger K, et al. Provision of STD Services in Community Settings After the Loss and Return of State Funding to Support Service Provision: Observations From Select Providers in Massachusetts, 2010 and 2013. Journal of public health management and practice : JPHMP. 2020;26(1):E18-E27.

128. Maaskant JM, Jongerden IP, Bik J, Joosten M, Musters S, Storm-Versloot MN, et al. Strict isolation requires a different approach to the family of hospitalised patients with COVID-19: A rapid qualitative study. International journal of nursing studies. 2021;117:103858.
129. MacCarthy S, Barreras JL, Mendoza-Graf A, Galvan F, Linnemayr S. Strategies for Improving Mobile Technology-Based HIV Prevention Interventions With Latino Men Who Have Sex With Men and Latina Transgender Women. AIDS Educ Prev. 2019;31(5):407-20.
130. McDonald R, Eide D, Abel-Ollo K, Barnsdale L, Carter B, Clausen T, et al. A rapid assessment of take-home naloxone provision during COVID-19 in Europe. International Journal of Drug Policy. 2022;107:103787.
131. McNall MA, Welch VE, Ruh KL, Mildner CA, Soto T. The use of rapid-feedback evaluation methods to improve the retention rates of an HIV/AIDS healthcare intervention. Evaluation and Program Planning. 2004;27(3):287-94.
132. Minoyan N, Hoj SB, Zolopa C, Vlad D, Bruneau J, Larney S. Self-reported impacts of the COVID-19 pandemic among people who use drugs: a rapid assessment study in Montreal, Canada. Harm Reduction Journal. 2022;19(1):38.
133. Murray DD, Babiker AG, Baker JV, Barkauskas CE, Brown SM, Chang CC, et al. Design and implementation of an international, multi-arm, multi-stage platform master protocol for trials of novel SARS-CoV-2 antiviral agents: Therapeutics for Inpatients with COVID-19 (TICO/ACTIV-3). Clinical Trials. 2022;19(1):52-61.
134. Nevedal AL, Reardon CM, Opra Widerquist MA, Jackson GL, Cutrona SL, White BS, et al. Rapid versus traditional qualitative analysis using the Consolidated Framework for Implementation Research (CFIR). Implementation Science. 2021;16(1):67.
135. Palinkas LA, Whiteside L, Nehra D, Engstrom A, Taylor M, Moloney K, et al. Rapid ethnographic assessment of the COVID-19 pandemic April 2020 'surge' and its impact on service delivery in an Acute Care Medical Emergency Department and Trauma Center. BMJ Open. 2020;10(10):e041772.
136. Palinkas LA, De Leon J, Salinas E, Chu S, Hunter K, Marshall TM, et al. Impact of the COVID-19 pandemic on child and adolescent mental health policy and practice implementation. International Journal of Environmental Research and Public Health. 2021;18(18) (no pagination)(9622).
137. Palinkas LA, Springgate BF, Sugarman OK, Hancock J, Wennerstrom A, Haywood C, et al. A Rapid Assessment of Disaster Preparedness Needs and Resources during the COVID-19 Pandemic. International Journal of Environmental Research & Public Health [Electronic Resource]. 2021;18(2):07.
138. Rains E, Stuck A, Ko KJ, Crowley C, Zifferblatt J. Advancing alternative care pathways in geriatric acute unplanned care. Journal of the American Geriatrics Society. 2020;68(SUPPL 1):S130.
139. Renfro CP, Rome Z, Gatwood J, Hohmeier KC. Use of Rapid Assessment Procedures when analyzing qualitative data in pharmacy research. Research In Social & Administrative Pharmacy. 2022;18(1):2249-53.
140. Rodriguez R, Torres J, Chang A, Haggins A, Caldwell M, Miller D, et al. 54 The Rapid Evaluation of COVID-19 Vaccination in Emergency Departments for Underserved Patients Study. Annals of Emergency Medicine. 2021;78(2 Supplement):S28.
141. Rolf F, Campbell N, Thompson S, Argus G. Australians' Experience of the COVID-19 Pandemic: Advantages and Challenges of Scaling Up Qualitative Research Using Large-Scale Rapid Analysis and Building Research Capacity Across Rural Australia. International Journal of Qualitative Methods. 2021;20:16094069211051937.
142. Romeu-Labayen M, Tort-Nasarre G, Alvarez B, Subias-Miquel M, Vazquez-Segura E, Marre D, et al. Spanish nurses' experiences with personal protective equipment and perceptions of risk of contagion from COVID-19: A qualitative rapid appraisal. Journal of Clinical Nursing. 2022;31(15-16):2154-66.

143. Rosteius K, De Boer B, Staudacher S, Verbeek H. Building trusting relationships with staff members of nursing homes during rapid ethnographic research. Frontiers in Sociology. 2022;7:983728.
144. Scott K, Guigayoma J, Palinkas LA, Beaudoin FL, Clark MA, Becker SJ. The measurement-based care to opioid treatment programs project (MBC2OTP): a study protocol using rapid assessment procedure informed clinical ethnography. Addiction Science & Clinical Practice. 2022;17(1):44.
145. Seay J, Ranck A, Weiss R, Salgado C, Fein L, Kobetz E. Understanding Transgender Men's Experiences with and Preferences for Cervical Cancer Screening: A Rapid Assessment Survey. LGBT health. 2017;4(4):304-9.
146. Seay JS, Carrasquillo O, Campos NG, McCann S, Amofah A, Pierre L, et al. Cancer Screening Utilization Among Immigrant Women in Miami, Florida. Prog. 2015;9 Suppl:11-20.
147. Shaw J, Feeney H, Meunier-Sham J, Hazard K, Plante P, Petricone R. An evaluation of a rapid conversion to teleSANE in response to COVID-19. American Journal of Community Psychology. 2022;28:28.
148. Shimkhada R, Attai D, Scheitler AJ, Babey S, Glenn B, Ponce N. Using a Twitter Chat to Rapidly Identify Barriers and Policy Solutions for Metastatic Breast Cancer Care: Qualitative Study. JMIR Public Health and Surveillance. 2021;7(1):e23178.
149. Short NJ, Borthakur G, Pemmaraju N, Dinardo CD, Kadia TM, Jabbour E, et al. A multi-arm phase Ib/II study designed for rapid, parallel evaluation of novel immunotherapy combinations in relapsed/refractory acute myeloid leukemia. Leukemia & Lymphoma. 2022:1-10.
150. Solomon P, Tennille J, Lipsitt D, Plumb E, Metzger D, Blank MB. Rapid assessment of existing HIV prevention programming in a Community Mental Health Center. Journal of Prevention and Intervention in the Community. 2006;33(1-2):137-51.
151. Srinivasan M, Asch S, Vilendrer S, Thomas SC, Bajra R, Barman L, et al. Qualitative Assessment of Rapid System Transformation to Primary Care Video Visits at an Academic Medical Center. Annals of Internal Medicine. 2020;173(7):527-35.
152. Talati AN, Mallampati D, Johnson JD, West-Honart A, Vladutiu C, Menard MK. 1009 Provider satisfaction with telehealth for maternity care: a rapid assessment of a national survey. American Journal of Obstetrics and Gynecology. 2021;224(2 Supplement):S625.
153. Talwai A, Wing V, Itzkovich Y, Galaznik A, Chatterjee A, Jain R, et al. PIN83 The COVID-19 Research Database: Building One of the Largest PRO Bono Real-World DATA Repositories. Value in Health. 2021;24(Supplement 1):S121.
154. Tan RKJ, Lim JM, Lo JJ, Teo AKJ, O'Hara CA, Ching AH, et al. Conducting rapid qualitative research to support sex workers' health and social needs in the face of COVID-19: capitalising on stakeholder networks from the HIV response in Singapore to drive policymaking. Sexually Transmitted Infections. 2021;97(2):84.
155. Taubenberger S, Spencer N, Chang JC, Paul N, Fabre S, Jagessar B, et al. A rapid-cycle assessment strategy for understanding the opioid overdose epidemic in local communities. Substance Abuse. 2021;42(4):888-95.
156. Tort-Nasarre G, Alvarez B, Galbany-Estragues P, Subias-Miquel M, Vazquez-Segura E, Marre D, et al. Front-line nurses' responses to organisational changes during the COVID-19 in Spain: A qualitative rapid appraisal. Journal of Nursing Management. 2021;29(7):1983-91.
157. Toth G, Limburg H, Szabo D, Sandor GL, Nagy ZZ, Nemeth J. Rapid assessment of avoidable blindness-based healthcare costs of diabetic retinopathy in Hungary and its projection for the year 2045. British Journal of Ophthalmology. 2021;105(8):1116-20.
158. Vazquez E, Chobdee J, Nasrollahzadeh N, Cheney A. Personal Freedom and Social Responsibility in Slowing the Spread of COVID-19: A Rapid Qualitative Study. Health Education & Behavior. 2022;49(1):26-34.

159. Wesolowski A, Gibson D, Agarwal S, Lambrou A, Kirk G, Labrique A, et al. Rapid assessments of non-pharmaceutical intervention uptake and population mobility patterns elucidate SARS-CoV-2 transmission dynamics. Open Forum Infectious Diseases. 2020;7(SUPPL 1):S848.
160. Wichmann O, Stocker P, Poggensee G, Altmann D, Walter D, Hellenbrand W, et al. Pandemic influenza A(H1N1) 2009 breakthrough infections and estimates of vaccine effectiveness in Germany 2009-2010. Eurosurveillance. 2010;15(18):1-4.
161. Porter A, Black S, Dale J, Harris-Mayes R, Lawrenson R, Lyons R, et al. Electronic records in ambulances-an observational study (ERA). Emergency Medicine Journal. 2019;36 (10):E14.
162. Wiss DA, Schellenberger M, Prelip ML. Rapid Assessment of Nutrition Services in Los Angeles Substance Use Disorder Treatment Centers. Journal of Community Health. 2019;44(1):88-94.
163. Xiang JJ, Roy A, Summers C, Delvy M, O'Donovan JL, Christensen J, et al. Improving hematology/oncology clinical research recruitment via universal prescreening: The VA Connecticut (VACT) Cancer Center experience. Journal of Clinical Oncology Conference. 2021;39(28 SUPPL).
164. Agaku IT, Nkosi L, Agaku QD, Gwar J, Tsafa T. A Rapid Evaluation of the US Federal Tobacco 21 (T21) Law and Lessons From Statewide T21 Policies: Findings From Population-Level Surveys. Preventing Chronic Disease. 2022;19:E29.
165. Ash JS, Sittig DF, McMullen CK, Guappone K, Dykstra R, Carpenter J. A rapid assessment process for clinical informatics interventions. Amia 2008;Annual Symposium proceedings / AMIA Symposium. AMIA Symposium.:26-30.
166. Belford M, Robertson T, Jepson R. Using evaluability assessment to assess local community development health programmes: a Scottish case-study. BMC medical research methodology. 2017;17(1):70.
167. Haigh F, Harris E, Chok HN, Baum F, Harris-Roxas B, Kemp L, et al. Characteristics of health impact assessments reported in Australia and New Zealand 2005-2009. Australian and New Zealand journal of public health. 2013;37(6):534-46.
168. Hailey D. The use and impact of rapid health technology assessments. 2001.
169. Lewinski AA, Crowley MJ, Miller C, Bosworth HB, Jackson GL, Steinhauser K, et al. Applied Rapid Qualitative Analysis to Develop a Contextually Appropriate Intervention and Increase the Likelihood of Uptake. Medical Care. 2021;59(Suppl 3):S242-S51.
170. Li Y, Li M, Rice M, Su Y, Yang C. Phased Implementation of COVID-19 Vaccination: Rapid Assessment of Policy Adoption, Reach and Effectiveness to Protect the Most Vulnerable in the US. International Journal of Environmental Research & Public Health [Electronic Resource]. 2021;18(14):19.
