# Supplementary material for: Approaches to enabling rapid evaluation of innovations in health and social care: a scoping review of evidence from high-income countries
Source: BMJ Open. 2022 Dec 20;12(12):e064345. doi: 10.1136/bmjopen-2022-064345 (PMC10580278; doi:10.1136/bmjopen-2022-064345)
Supplement: Author's manuscript [file bmjopen-2022-064345.draft_revisions.pdf]

BMJ Open is committed to open peer review. As part of this commitment we make the peer review history of every article we publish publicly available.

When an article is published we post the peer reviewers' comments and the authors' responses online. We also post the versions of the paper that were used during peer review. These are the versions that the peer review comments apply to.

The versions of the paper that follow are the versions that were submitted during the peer review process. They are not the versions of record or the final published versions. They should not be cited or distributed as the published version of this manuscript.

BMJ Open is an open access journal and the full, final, typeset and author-corrected version of record of the manuscript is available on our site with no access controls, subscription charges or pay-per-view fees (http://bmjopen.bmj.com).

If you have any questions on BMJ Open's open peer review process please email info.bmjopen@bmj.com

BMJ Open

Rapid Evaluations of Innovations: a scoping review

Journal:	*BMJ Open*
Manuscript ID	bmjopen-2022-064345
Article Type:	Original research
Date Submitted by the Author:	03-May-2022

Complete List of Authors:	Norman, Gill; The University of Manchester Faculty of Biology Medicine and Health<br>Mason, Thomas; University of Manchester<br>Dumville, Jo C.; The University of Manchester<br>Bower, Peter; The University of Manchester Faculty of Biology Medicine and Health, NPCRDC<br>Wilson, Paul; University of Manchester, Alliance Manchester Business School<br>Cullum, Nicky; The University of Manchester Faculty of Biology Medicine and Health, School of Health Sciences; Manchester University NHS Foundation Trust, MAHSC

Keywords:	STATISTICS & RESEARCH METHODS, Organisation of health services < HEALTH SERVICES ADMINISTRATION & MANAGEMENT, Quality in health care < HEALTH SERVICES ADMINISTRATION & MANAGEMENT

SCHOLARONE™
Manuscripts

I, the Submitting Author has the right to grant and does grant on behalf of all authors of the Work (as defined in the below author licence), an exclusive licence and/or a non-exclusive licence for contributions from authors who are: i) UK Crown employees; ii) where BMJ has agreed a CC-BY licence shall apply, and/or iii) in accordance with the terms applicable for US Federal Government officers or employees acting as part of their official duties; on a worldwide, perpetual, irrevocable, royalty-free basis to BMJ Publishing Group Ltd ("BMJ") its licensees and where the relevant Journal is co-owned by BMJ to the co-owners of the Journal, to publish the Work in this journal and any other BMJ products and to exploit all rights, as set out in our licence.

The Submitting Author accepts and understands that any supply made under these terms is made by BMJ to the Submitting Author unless you are acting as an employee on behalf of your employer or a postgraduate student of an affiliated institution which is paying any applicable article publishing charge ("APC") for Open Access articles. Where the Submitting Author wishes to make the Work available on an Open Access basis (and intends to pay the relevant APC), the terms of reuse of such Open Access shall be governed by a Creative Commons licence – details of these licences and which Creative Commons licence will apply to this Work are set out in our licence referred to above.

Other than as permitted in any relevant BMJ Author's Self Archiving Policies, I confirm this Work has not been accepted for publication elsewhere, is not being considered for publication elsewhere and does not duplicate material already published. I confirm all authors consent to publication of this Work and authorise the granting of this licence.

For peer review only

Rapid Evaluations of Innovations: a scoping review

Gill Norman[1,3], Thomas Mason[2,3], Jo Dumville[1,3], Peter Bower[2,3], Paul Wilson[2,3], Nicky Cullum[1,3]

Affiliations

1. Division of Nursing, Midwifery & Social Work; School of Health Sciences; Faculty of Biology, Medicine & Health, University of Manchester, Manchester, UK.

2. Centre for Primary Care and Health Services Research, School of Health Sciences; Faculty of Biology, Medicine & Health, University of Manchester; Manchester, UK.

3. Manchester Academic Health Science Centre, Research and Innovation Division, Manchester University Foundation NHS Trust, Manchester, UK.

Address for correspondence

Gill Norman
Division of Nursing, Midwifery & Social Work;
School of Health Sciences;
Faculty of Biology, Medicine & Health,
University of Manchester
Oxford Road
Manchester
M13 9PL
E: gill.norman@manchester.ac.uk

Word count: 4414

Keywords: Rapid evaluation; Methods, Healthcare

Article Summary: Strengths and Limitations

- This review uses established scoping methodology, including a registered protocol, to map the evidence relating to rapid evaluation in health and related social care.
- Multiple databases and other sources were searched to identify relevant publications.
- High literature volume meant not all review processes could be implemented in duplicate.
- Clear evidence coding enabled the identification of distinct approaches which are reported as being rapid evaluation, and of individual comparisons of rapid and non-rapid approaches.

For peer review only - http://bmjopen.bmj.com/site/about/guidelines.xhtml

ABSTRACT

Objectives: To map the developing field of methods of rapid evaluation of innovation in health and related social care

Design: Scoping review

Setting: High income countries, health and care contexts

Methods: We searched multiple databases and websites of key organisations to identify rapid evaluations of innovations, methods of rapid evaluation or rapid evaluation of implementation in health and care in high income countries. We excluded regulatory evaluations.

We extracted information to enable us to classify and map existing studies on key characteristics. We undertook a narrative synthesis to identify the evidence and the gaps, focussing on the different approaches to conducting rapid evaluation in primary research.

Results: We identified 14069 records from our searches, evaluated 352 in full text and included 72 studies. We identified four main approaches used for rapid evaluation:

(1) Use of a methodology designed specifically for rapid evaluation;
(2) Increasing rapidity by doing less or using a less time-intensive methodology;
(3) Use of alternative technologies and/or data to increase the speed of an existing evaluation method;
(4) Adaptation of part of a non-rapid evaluation

We found little research comparing rapid and non-rapid evaluation.

Conclusions: This scoping review identified a lack of clarity about what ‘rapid evaluation’ means but identified useful preliminary categories. There is a need for clarity and consistency in terms of: what constitutes rapid evaluation; consistent terminology in reporting evaluations as rapid; the development of specific methodologies for making evaluation more rapid; and assessment of the advantages and disadvantages of rapid methodology in terms of rigour, cost and impact.

Registration: OSF.io/np72f

For peer review only - http://bmjopen.bmj.com/site/about/guidelines.xhtml

INTRODUCTION

Background

There is increasing demand for timely, rigorous evaluation of innovation in health and social care. Demand has been amplified during the COVID-19 pandemic, where the ability of research infrastructure to respond rapidly has been paramount.

We adopt the definition of innovation as: *any novel technology, device, procedure, set of behaviours, routine, or way(s) of working that is directed at improving health outcomes, administrative efficiency, cost-effectiveness or users' experience - and is implemented by planned and coordinated actions* (1). Thus, innovation can include alterations in service delivery, organisation and financing, as well as clinical and care practices.

Evaluation is a complex and multifaceted undertaking which lacks a single definition. Using a broad definition that encompasses research, typically evaluation investigates whether an overall net benefit is gained from innovation over alternative activities. Phase III randomised controlled trials (RCTs) and systematic reviews of innovations remain the 'gold-standard' of comparative effectiveness methods; although quasi-randomised approaches are often more feasible. For other uncertainties around innovations (e.g. acceptability, feasibility) there are well established quantitative, qualitative and mixed methods (and corresponding approaches to evidence synthesis).

Whilst the need for rapidity in evaluation is widely recognised, there has been limited assessment of how rapidity can be actually achieved (2-4); this is particularly the case for primary evaluations as opposed to rapid reviews (5-9).

The term 'rapid evaluation' is widely used to describe approaches that aim to adopt pragmatic methods for timely assessment of innovations, but there is uncertainty about what this means in practice. Rapid evaluation can be conceptualised to include rapid conception and inception of evaluation in response to an identified need, or rapid completion of part or all of the evaluation process, but linking these ideas to practice still requires investigation.

We undertook a scoping review to map reports of rapid evaluations of health and social care innovations; map the types of question asked in rapid evaluations and to identify where further research is needed to support more rapid evaluation. We focused the review on studies from high-income countries to increase relevance to the National Health Services in the UK.

For peer review only - http://bmjopen.bmj.com/site/about/guidelines.xhtml

Research Questions

In this scoping review we explored the following research questions:

- What methodologies and methods relevant to a UK context have been used to undertake rapid evaluations of health and social care innovations? How are these approaches expected to deliver rapidity?

- What is the comparative evidence for rapid evaluation methodologies? Have these methodologies been compared to non-rapid standard (however defined) methods or other rapid methodologies?

METHODS

Approaches to scoping for rapid evaluations

In this scoping review, we aimed to: identify and summarise evaluations addressing health-related questions in ways which had been described as 'rapid'; document the ways in which study authors considered that evaluation was made 'rapid'; and summarise evidence that compares the risks and benefits of rapid evaluation and alternative approaches. We accepted authors' definitions of what constituted 'rapid evaluation'. We considered evaluations of the safety and effectiveness of innovations as well as a wider set of outcomes, including acceptability and user experience. In this scoping review we did not seek to assess the quality of studies.

We adopted an iterative approach to review methods which we planned for in our protocol, which was completed before the screening stage of the review and published on OSF (10). We have followed the reporting approach recommended in the extension of PRISMA for scoping reviews (PRISMA-ScR) where possible given this planned flexibility (11).

Patient and Public Involvement

No patients or members of the public were involved.

Inclusion eligibility

Our inclusion criteria (Table 1) were developed using a PICOS approach and used to screen the results of the database search. We developed the inclusion criteria iteratively to ensure that the most relevant research was captured, anticipating this flexibility in the protocol.

We only included publications in English. We planned to document relevant non-English publications but not to extract data or include them due to the available resources. However, the non-English publications identified were ineligible (based on assessment of an English language abstract).

For peer review only

Search

We searched Ovid MEDLINE, EMBASE and Health Management Information Consortium (HMIC) databases to February 2020. We also looked at websites of key organisations involved in rapid evaluations (e.g. RSET, BRACE, Nuffield Trust and Health Foundation, THIS Institute) and checked references of identified studies. Finally, we searched Google Scholar and checked the first 1000 records against the main database searches. The database search strategy (Appendix 1) was implemented by an information specialist. We selected terms to balance sensitivity and specificity. There were two main elements to the search: a search for rapid evaluations undertaken from database inception, and a separate search from 2014 onwards using the search strategy from a previous, high-quality scoping review of rapid reviews which had searched up to 2014 (5). We subsequently narrowed the scope of our search to exclude rapid reviews (Table 1).

Table 1: Eligibility Criteria

**Study design:** Publications which evaluate, compare, document the use of, provide guidance on, or otherwise describe one or more rapid evaluation methods for assessing innovations according to the study authors' definition (of rapid evaluation). We did not include rapid reviews or papers relating to methods for rapid reviews. Where studies used a rapid review as the first stage of primary evaluation, we included them only if the primary evaluation element was also described as rapid or using rapid methods. Evaluations could be primarily quantitative or qualitative in focus (or use mixed methods). We also excluded pilot studies which were not explicitly described as rapid evaluations.
**Innovations (interventions):** Publications on rapid evaluation of innovations or their implementation in the field of health and of social care as it relates to health. Innovation is broadly defined as encompassing intentional change.(1) We subsequently broadened this to encompass interventions or services not described as innovations. We excluded publications reporting methods which related only tangentially to interventions or services such as Rapid Assessments of Needs and Rapid Epidemiological Assessments.
**Populations:** We included any study relating to rapid evaluation methods and did not further limit eligibility based on the study population beyond the requirement that the study be undertaken in a health or related care setting in a high-income country using World Bank definitions.(12) We also excluded studies which looked at evaluation in a regulatory context because these were more closely related to review methodology.
**Outcomes:** We accepted any health-related outcome measure; this includes any clinical measure relevant to the intervention including patient-reported outcomes or experience; measures of care process or service use; and measurements of costs or

For peer review only - http://bmjopen.bmj.com/site/about/guidelines.xhtml

activity as well as health economic measures such as cost-effectiveness. This was used to ensure the relevance of the study to health and related social care.

For peer review only

For peer review only - http://bmjopen.bmj.com/site/about/guidelines.xhtml

Screening

One researcher screened initial search results by title and abstract in Endnote and selected potentially relevant records for full text evaluation.(13) A 5% sample was exported to Microsoft Excel (Version 365) and screened by a second researcher for consistency. Records obtained at full text were screened by one researcher, with a second researcher assessing 10% to check consistency and was subsequently available for discussion in cases of uncertainty. We did not undertake duplicate screening for all records because of the high volume of retrieved records and because the sample which were duplicate screened showed a very high level of consistency (at abstract stage only a single record was identified which had been excluded in error by the first screen).

Data extraction

We extracted sufficient information to enable us to classify studies on their key characteristics and to thereby 'chart' the available literature (11, 14). Following the recommendations of Levac, (15) we piloted a bespoke data form using Microsoft Excel to enable data extraction, using two researchers, with a small sample of included studies. The following data were extracted by one researcher (with a 10% sample cross-checked by a second researcher, who was also consulted in cases of uncertainty).

- Study ID (First author, year of publication)
- Country (individual); country group (Africa, Asia/middle east, Europe, North America, Central/South America), OECD class (LMIC or high income)
- Definition(s) of rapid evaluation used by authors including timescale and whether the focus was evaluation/implementation/methods
- Study designs as described by authors (qualitative/quantitative/mixed methods)
- Question(s) the evaluation sought to address
- Collection/analysis methods (e.g. survey, observation, interviews)
- Nature of innovations evaluated (if practical example)
- Impact of rapid methods (for comparative studies)
- Stage, scope, characteristics and purpose of evaluation e.g. local/regional/national; formative/iterative/summative
- Evaluation methods adaptation to context/complexity of intervention including whether stakeholder participation was reported. This was based on the relevant items from the TiDIER scale.(16)

We undertook this charting across all studies that met our inclusion criteria. These data were then used to map studies in terms of what made the study 'rapid'. The classification was undertaken by one researcher and discussed by the review team.

Summaries of study characteristics are provided in Appendix 2 (Supplementary Table 1, Supplementary Table 2 and Supplementary Table 3)

For peer review only - http://bmjopen.bmj.com/site/about/guidelines.xhtml

Synthesis and analysis

We developed a narrative synthesis of the evidence relating to each of our two review questions; this was supported by structured tables to inform the themes identified. We structured the synthesis by which question the evidence was addressing and by the methods used to make evaluation rapid.

RESULTS

Results of the search and screening process

We identified 14,069 unique records (Figure 1). Preliminary assessment determined 1464 records to be potentially eligible from which we excluded 1044 records of rapid reviews. The remaining 420 records were assessed in full text and reduced to 352 records detailing rapid evaluations of innovations. We excluded 226 records of studies in LMICs and 54 which looked at regulatory process evaluations (a list of excluded studies is available on request). We included 72 studies in the review (Table 2); these were methods papers, primary evaluations or evaluations of implementation undertaken in high income countries. Most studies were undertaken in primary care, community settings, or in specific non-hospital-based services (often described as ‘clinics’), reflecting the health and related care focus of the review.

For peer review only

Table 2: Summary table of included studies

**Means of making evaluation rapid** [1] [2]	**Examples of approach**
Methods specifically designed for rapid evaluation: 42 studies with supporting methods papers	Rapid assessment/appraisal process/procedure and adaptations (17-48)<br><br>Rapid assessment and response and adaptations (22, 23, 28, 49-57)<br><br>Rapid participatory appraisal and adaptations (33, 34, 58-63)<br><br>Rapid ethnographic assessment/ethnography and adaptations (64-73)<br><br>Methods from quality improvement (74-81)
Doing less or using a less time-intensive version of an existing methodology: 13 studies	Shorter research period (82-85)<br><br>Less intensive method (44, 86, 87)<br><br>Fewer participants or fewer/smaller study sites (68, 86, 88-92)
Adaptation of part of a standard method: 15 studies	Priority setting (93, 94)<br><br>Ethics approval, recruitment and participation, barriers (95-102)<br><br>Study design (103, 104)<br><br>Data collection and analysis (104-108)
Use of alternative technology or data source: 10 studies	Automation or otherwise increased speed of data collection, collation or analysis (44, 86, 109, 110)<br><br>Acquisition or use of existing data sets (105, 111-115)

[1] Some studies appear in more than one category because multiple means were employed
[2] Some studies in each category are represented by multiple references

For peer review only - http://bmjopen.bmj.com/site/about/guidelines.xhtml

Methodologies and methods used for rapid evaluation

After mapping included studies, we identified four categories that summarise approaches employed with the aim of achieving rapid evaluation (Table 2):

(1) Use of a methodology designed specifically for rapid evaluation which involved the whole research process

(2) Increasing rapidity by doing less, or using a less time-intensive methodology

(3) Adaptation of a non-rapid evaluation to make this a rapid part of the wider study (e.g., the recruitment stage of a trial or the analysis phase of a qualitative study)

(4) Use of alternative technologies and/or data sources to increase rapidity of an existing evaluation methodology

Some studies used more than one approach to rapidity; (for example an adaptation of a part of a wider study combined with the use of an alternative technology) and are therefore represented in multiple categories.

Below we expand on each category, giving illustrative examples for each.

1. ***Methodology specifically designed for rapid evaluation (rapid methodology)***

The largest number of papers concerned methodological approaches used specifically for rapid evaluation (examples and methodologies detailed in Appendix 2, Supplementary Table 4). These were represented by 42 studies (17-29, 31-33, 35-49, 51-67, 69-79, 94, 116). Some studies are represented by more than one publication and by supporting methods papers identified from references.

Examples include methods described variously as rapid assessment procedures or processes (22, 23); rapid evaluation (41); rapid appraisal procedures or processes (37, 39, 45) and rapid ethnographic assessments, ethnographic appraisals or ethnography (64-67, 69). Many of these approaches were closely related while some were explicit adaptations of other rapid approaches, such as rapid appraisal (45), rapid participatory appraisal (33, 62) rapid participatory appraisal with meta-planning (61), qualitative rapid appraisal, rigorous analysis (Q-RARA)(43), rapid assessment and response (RAR) (28), rapid assessment response and evaluation (RARE) (53); and rapid assessment procedure informed clinical ethnography (RAPICE) (70, 71). Four studies described themselves as adaptations of rapid assessment processes for specific evaluation purposes (17, 18, 21, 24). Some approaches, such as ‘plan, do, study, act’ and rapid cycle evaluation were drawn from areas such as quality improvement and wider implementation science (75, 76, 78). Three of the papers examined limitations of a particular methodology or looked at the impact of rapid

For peer review only - http://bmjopen.bmj.com/site/about/guidelines.xhtml

applications of these methodologies (31, 72, 73). This included highlighting the need for researchers to have received an appropriate level of training in the methodology.

The overarching characteristics of the methodologies designed to increase rapidity were that they were qualitative or mixed methods. None were exclusively quantitative nor were capable of *comparative* assessment of innovations (as is often required for health technology assessment). The actual methods employed in these studies shared core features including: multiple assessment methods (e.g., a combination of interviews of a small number of participants and wider surveys), and triangulation of data from the different approaches. The types of evaluation questions addressed in these studies largely focused on the applicability of previous research; acceptability of interventions; barriers and facilitators to intervention implementation or access; and on stakeholder views and experiences.

2. **Increasing rapidity by doing less, or using a less time-intensive version of an existing methodology**

The second approach to rapid evaluation involved undertaking evaluation on a smaller scale or in a less-intensive way (Table 3); sometimes with overlap with use of an alternative technology (see section 4 below) (44, 86). We identified 13 studies of this type (44, 45, 68, 82-86, 88-92).

Sometimes authors carried out a smaller study, with fewer sites and/or participants meaning that less data collection and analysis was required. Shortening the data-collection period was also adopted (82-84). One of these studies (85) comprised one of the few comparative studies identified in the review (see section below). Some studies balanced the impact of using smaller samples by employing techniques identified in methods designed specifically for rapid evaluation above, such as stating that they used random or pseudo-random participant/site selection, or selected a subsample of participants in such a way to ensure representativeness (86, 91, 117, 118). In other cases, no such measures were reported; such studies accepted – explicitly or implicitly – the trade-off between rapidity and representativeness or reliability of research (92). An alternative approach involved using a less-intensive method resulting in reduced information collected for each participant or site. This included using a briefer form of a research tool (survey or questionnaire) (86, 87).

For peer review only - http://bmjopen.bmj.com/site/about/guidelines.xhtml

Table 3: Approaches to increasing rapidity through less evaluation/less time-intensive evaluation

Approach employed with aim of increasing rapidity	Methods	Example
Shortening response period	Survey; safety monitoring cohort. Survey also used increased number of reminders to improve response rate	Joseph 2003 (82), Demeulemeester 2017 (83), Ghandi-Banga 2018 (84)
Using telephone instead of face-to-face interviews	Interviews	Williams 2019 (44), Wichmann 2009 (86)
Using a rapid interview method	Interviews; need to apply rapid method appropriately noted	Hamilton 2013 (87)
Using fewer participants in interviews	Interviews. Additional measures included random or quasi-random sampling of participants; use of samples selected for representativeness; use of small core group for all stages of project.	Wichmann 2009 (86), Dunbar 2018 (88), Brown-Johnson 2019 (89), Hamaraman 2014 (90), Quintero Romero 2006 (68), Loosier 2010 (91)
Using fewer study sites	Random or quasi-random site selection (e.g. housing blocks; in some instances the selection of sites/participants is the same process.	Brown-Johnson 2019, Hamaraman 2014 (89, 90), Cakir 2008 (117)
Evaluating a small/low volume site	Site audit/evaluation – acknowledged small site volume made rapidity possible	Delaney 2019 (92)
Using a shorter timescale for pre/post data periods	Interrupted time series – non-rapid application of method also used (more pre and post data).	McCarthy 2019 (85)

For peer review only - http://bmjopen.bmj.com/site/about/guidelines.xhtml

3. Adaptation of part of a standard evaluation method

Fifteen studies reported methods they used with the aim of increasing the rapidity of a discrete element or stage of an evaluation, where the evaluation as a whole used standard methods (Table 4).(93-108) This approach usually targeted the earlier stages of an evaluation, including research priority setting (93, 94), ethical approval (95), and participant identification and recruitment. At later stages of the evaluation process we identified continuous collection or data analyses as a means to increase rapidity of evaluation in RCTs (104, 106). In qualitative studies the focus was on rapid methods for data analysis (107, 108), including one study which compared rapid and standard analysis (see below) (107). Here, as in section 2 above there was some overlap with the use of technological innovation to increase rapidity (see section 4 below) (105).

For peer review only

For peer review only - http://bmjopen.bmj.com/site/about/guidelines.xhtml

Table 4: Adaptation of a discrete stage of a non-rapid evaluation to make this a rapid part of the wider study

Study stage targeted	Rapid methods	Examples
Priority setting	To improve rapidity of research question prioritisation (applicable to multiple designs)	Gray 2017, Newlands 2018 (93, 94)
Ethics approval	Intended to reduce time to obtain ethical approval by addressing requirements for multiple approvals (applicable to multiple designs)	Khan 2014 (95)
Recruitment and participation	Reducing time taken to identify participants and complete recruitment through methods to pre-specify or identify potential participants (primarily in RCTs)	Bower 2015, Brown 2019, Deane 2017 (96-98)
Rapid bilingual appraisal	Reducing barriers to inclusion in rapid evaluation; applicable to multiple study designs where language is a potential barrier	Whelan 2004 (99)
Assessment of barriers to process	Tool for rapid assessment of barriers to process; assessed within an RCT	Alolod 2015, 2016 Traino 2012 (100-102)
Structure of treatment groups/assignment	Proposal for flexible structuring of treatment assignment and use of single control group for multiple potential vaccine candidates in multi-stage RCT	Moodie 2013 (103)
Nesting of additional studies	Continuous rapid data analysis in realist study embedded in standard RCT	Hensel 2016 (104)
Data collection	Bespoke reporting platform; cohort study protocol to rapidly assess vaccines as well as epidemiology of disease	Simpson 2015 (105)

For peer review only - http://bmjopen.bmj.com/site/about/guidelines.xhtml

	Data tracking and rapid continuous feedback to participants (My Own Health Report). Designed to provide continuous collection and analysis of data in a pragmatic RCT	Glasgow 2014 (106)
Data analysis	Rapid analysis of data with or without other rapid stages; used in qualitative (comparative) study; embedded realist study; cohort study.	Taylor 2018, Burgess-Allen 2010, Glasgow 2014, Hensel 2016 (104, 106-108)

For peer review only

4. Using alternative technology or data source

The use of different or newer technology to rapidly acquire and/or analyse routine data was another approach to making evaluation more rapid (Table 5). As we have noted there is some overlap between this and the classifications above, but our synthesis considered this separately as it represents a technological streamlining of an existing process rather than an adaptation of the research design (44, 86). These ten studies involved using technology to automate or otherwise try to improve rapidity of one or more of data collection, collation, or analysis (44, 86, 105, 109-115). Studies which involved acquiring or using existing data sets to enable modelling of effectiveness were also included (111, 114, 119). Seven of these studies were undertaken in the context of monitoring of uptake and safety profiles of vaccinations.

For peer review only

For peer review only - http://bmjopen.bmj.com/site/about/guidelines.xhtml

Table 5: Use of an alternative technology or data source

Study authors' suggested rapid innovation	Research design	Impact/intention of method	Example(s)
New digital systems	Registry study	Introduction of new computerised systems to assess vaccination coverage of routine immunisation programmes; computerised child registers also became available.<br><br>Computer-assisted telephone surveys. Authors stated that it enabled more timely feedback to co-ordinators of district vaccination programmes and rapid identification of random samples for interview	Begg1989 (109). Wichmann 2009 (86), Williams 2019 (44)
Using bespoke data platform	Cohort	Nationally representative sample assessed using linking of multiple data sources from clinical practice, laboratory results and death certification. Designed to assess pandemic influenza reporting and vaccination impact; plan to be implemented for future influenza pandemic	Simpson 2015 (105)
Secure web-based tool for pharmacovigilance	Cohort	Secure web-based tool for small number of set fields focused on single medications in hospice context using rapid prospective reporting at agreed time-points to allow rapid aggregation of data on small numbers of participants from multiple centres	Currow 2012 (110)

For peer review only - http://bmjopen.bmj.com/site/about/guidelines.xhtml

Automation of data collection	Registry study	Use of hospital routine data collection to populate registry with validation to analyse costs for two different methods of triage of designated patient group	Noack 2018 (111)
Automation of data processing	Registry study	Use of regional/organisational vaccine information system to rapidly assess influenza vaccine campaign safety and coverage	Alguacil-Ramos 2019 (115), CDC 2005(112)
Use of existing data sets	Registry-based studies	Use of data from (e.g.) an insurance database or hospital data registry to populate a comparison of a specified adverse event or to analyse costs	Gagne 2013 (113),Noack 2018 (111)
Simulation of impact of vaccination using existing health care data sets	Simulation study	Use of established data sets to run simulations on how vaccine coverage and population characteristics impact ability to detect safety signals for influenza vaccinations. The authors recommended this be used only as a method to detect signals and generate hypotheses.	Dodd 2018 (114)

For peer review only

Studies comparing a rapid with a non-rapid method

We found only two studies which compared a rapid evaluation method with a non-rapid method, and we did not find any comparisons of different types of rapid evaluations.

One study compared the use of rapid evaluation with standard evaluation of an enhanced health programme in care homes. The study was described as assessing rapid evaluation but the rapid evaluation itself took 2.5 years (85). Nevertheless, this was the only explicit comparison we found. This study assessed the cost consequences of implementing the programme and compared the use of shorter and longer-term interrupted time-series (ITS) analyses, using shorter and longer time-series of aggregated data (85). The authors found that the longer-term analyses yielded a different interpretation, an improved model fit and greater precision; and they considered that the shorter-term analyses produced results likely to be misleading. The time period required for acceptable internally valid ITS based studies is contested more generally (120-123).

A second study compared rapid with usual qualitative analysis in an evaluation of a home birth service in the UK (107). The study authors reported on the total time taken in the data-management process as well as the analysis component. The agreement between the findings and recommendations of the two processes was also considered. The authors concluded that rapid analysis delivered a modest timesaving which may have been greater if there had been greater concordance between the characteristics of the two research teams. They also reported substantial overlap in findings between the two analysis approaches, although their recommendations diverged more. The two analyses were undertaken by different research teams which had differing levels of contextual experience, different stakeholder audiences and differing opportunities for analysis discussion and this was identified as a limitation in the study, together with the fact that rapid analysis was a novel approach for the researchers involved. The study authors concluded that rapid analysis may potentially deliver valid findings while taking less time but recommended further comparisons using additional data sets with more comparable research teams.

For peer review only - http://bmjopen.bmj.com/site/about/guidelines.xhtml

DISCUSSION

Summary

This scoping review identified a lack of clarity in approaches described as 'rapid evaluation' in primary research. The approaches to rapid evaluation which we identified could be grouped into four broad categories (1) studies using specific methodologies designed for rapid evaluation; (2) studies which carried out less evaluation or less intensive evaluation; (3) studies which used rapid methods only for a particular stage of evaluation; and (4) studies which used technology to increase rapidity. There was very little comparative research on the impact of using rapid rather than non-rapid evaluation (85, 107).

It is notable that we did not identify many rapid evaluation methodologies clearly designed to assess the comparative effectiveness of innovations. Studies using bespoke, rapid methodologies used qualitative or mixed methods and mainly assessed aspects of user experience and acceptability along with associated barriers and facilitators, and the implementation of innovations in particular contexts. This may reflect the types of questions which are considered suitable for rapid evaluation and the impetus to develop rapid evaluation methods in these areas of research, but it may also reflect conceptions of what evaluation is possible within a limited timeframe.

Strengths and Limitations

Due to a high volume of identified literature at the initial and second stages of the review process we did not use duplicate screening and coding by two independent researchers for all studies. We acknowledge that this may have increased the risk of error or bias in the review processes. However, we screened and coded subsets of the records at each stage in duplicate and found very high levels of agreement, so we do not believe that the impact is likely to be substantial. This is particularly the case as we consulted a second reviewer in cases of uncertainty for other records, both in screening and in coding, meaning that consensus was used where judgments were difficult.

Bias is also possible because we employed a degree of flexibility and iteration in the selection of studies for the review. However, we prespecified this flexible approach to produce a review that would be relevant to the context of the NHS in England, while remaining comprehensive enough to capture relevant research methods. We have documented this in our methods and results to ensure transparency about the review processes.

A related limitation concerns the decision to limit our inclusion criteria to reports from high income countries, as we may have omitted methods for rapid evaluation which might be transferable to the UK context. We identified three types of studies where this might apply. The first relates to specific methods for rapid evaluation designed

For peer review only - http://bmjopen.bmj.com/site/about/guidelines.xhtml

and used solely in LMIC. We identified a substantial number of examples of rapid assessment of avoidable blindness (124) and adaptations of this approach may be relevant to health conditions seen in the UK. Secondly, we identified the use of lot-quality surveys (a method of using small sample sizes to determine if areas have achieved a specified target) as a method of doing research with fewer participants; this was only represented in studies from LMIC (117, 118). Finally, we found an example of a method of increasing rapidity by making part of a study more rapid which was only identified in LMIC contexts but may be particularly relevant to disadvantaged or marginalised participants in the UK. Six studies used preparatory methods to ensure informed consent so that enrolment for subsequent studies could be undertaken rapidly because necessary groundwork had been undertaken (99, 125-129). We note that some of our excluded studies from LMIC described their methods as 'rapid' but with limited information on what promoted rapidity. Our initial assessment suggested that evaluations completed within a period of weeks, or a few months was what qualified them as rapid in the eyes of the authors. It is possible that a systematic review with wider inclusion criteria and the resources to contact authors may have identified specific methods which would have relevance to UK settings, but which are not represented in either our included studies or the excluded studies summarised here.

Because this is a scoping review we did not conduct quality assessment of the identified studies; we therefore document the nature of the research available rather than assessing its reliability (14, 15). We do not attempt to evaluate the success or otherwise of methods proposed as enabling rapid evaluation but have accepted study authors' own assessments of the usefulness or success of their methods. A further limitation of this review is that we were unable to identify (and therefore include) studies that may have carried out work which represents rapid evaluation but have failed to describe it as such (for example, retrospective quantitative analyses). This might bias the mapping of studies identified if particular types of studies exhibit differential propensity to report themselves as rapid.

Conversely, we identified some evaluations which the study authors described as rapid, but which might not commonly be considered as rapid, except in comparison to an even more detailed and lengthy standard process. We explicitly excluded pilot studies (often RCTs) because these were considered to be assessing feasibility rather than undertaking evaluation (130). This may have contributed to a skewing of the literature identified towards non-quantitative methods, but the pilot studies we did identify did not label themselves as being rapid studies e.g. (131-133). We are aware that we may therefore have omitted some examples of studies which would meet some definitions of rapid. However, these limitations would not explain the relative paucity of studies comparing rapid to alternative methods in the identified literature, and this appears to be a genuine evidence gap.

Interpretation of the studies

For peer review only - http://bmjopen.bmj.com/site/about/guidelines.xhtml

The drive towards rapid evaluation raises tensions for those commissioning and delivering research. Evaluation studies aim to achieve rigour (broadly, internal and external validity, or their qualitative equivalents) and scope (answering a range of questions, including those relating to access, effectiveness, cost effectiveness, acceptability, equity and implementation). In most circumstances these must be achieved under constraints of cost and time. The greater the scope of the project, and the greater the protection against validity threats, the higher is the cost and time. These known trade-offs are being explored by organisations such as BRACE and RSET (134).

Occasionally, researchers can trade time against cost. For example, national recruitment drives (such as those under COVID in trials such as RECOVERY) have maintained high levels rigour under time pressure by deploying significant resources (135). Some of the factors delaying evaluation delivery can be managed through additional resources. However, some, such as long-term follow up, cannot be managed in that way. In regulatory contexts these trade-offs may be addressed through mechanisms of post-marketing surveillance (136), but this only applies to a subset of innovations such as pharmaceuticals or medical devices.

Where studies cannot be accelerated through additional resource, or where costs are constrained, rapid evaluation essentially involves trading off rigour or scope against time. Some of those trade- offs can be profound. For example, a study of an innovation might eschew questions of comparative effectiveness and cost-effectiveness entirely and restrict the scope of the study to issues of staff acceptability and implementation. This seemed to be the approach adopted in some of the studies identified in the review. Quantitative rapid evaluations are more likely to evaluate impacts of innovations on short-term indicators (such as processes of care) which are often proxies for outcomes where longer-term follow-up is required. Therefore, the limitations of such approaches are likely to depend on the strength and consistency of the relationship between the proxy and the ideal longer-term outcome. Some decision-making frameworks (such as the NICE digital framework) specifically identify some interventions where such trade-offs are legitimate (137). Contextual pressures (such as the pandemic) may force trade-offs to ensure timely decisions.

Other trade-offs will be more marginal. For example, a decision maker might accept a smaller sample (and hence a lower level of quantitative precision) or more selective recruitment (at some cost in external validity). It is also the case that reduced statistical power related to using shorter post-intervention time periods might be at least partly offset by using a comparative design (such as difference-in-differences) rather than interrupted time series, since estimates from comparative designs are based on more information than non-comparative approaches. Such trade-offs impact on rigour and scope in a more limited way that might be more acceptable. Larger trade-offs will be potentially easier to assess in terms of their impact – for example, eschewing questions of comparative effectiveness will, or

For peer review only - http://bmjopen.bmj.com/site/about/guidelines.xhtml

should, restrict what can be claimed about an innovation. Some trade-offs are more difficult to judge in terms of their impact.

The role of rapid evaluation methodology is to reduce impacts on rigour and scope as far as possible, while maintaining the benefits in terms of time. We identified several examples of these in the literature, although there was limited comparative work on their success (85, 107). Since conducting prospective comparisons of rapid and non-rapid evaluation methods is time-consuming and resource intensive, it may be possible to use retrospective analysis of existing data to explore the impact of using shorter analysis periods. For example, it would be possible to conduct a sensitivity analysis of truncated sets of timepoints in an interrupted time series, and to compare the results to the full analysis. Performing such analyses on several sets of data from different studies would allow assessment of whether there was a consistent impact on results but would be possible with only a limited set of study designs. In this the studies relating to regulatory decision-making, excluded from this review, may provide useful design features.

Future research

There is a need for clarity and consistency in terms of what constitutes rapid evaluation and, in particular, what differentiates rapid from non-rapid evaluation. Consensus on definitions and more consistent labelling will be important in allowing ongoing assessment of this developing field. We suggest that better description would involve clarity about what aspects of the research process were conducted more rapidly, when this occurred in the research process, and what the potential impacts could be.

The development of specific rapid methodologies and technologies is needed to provide a toolkit for research teams to meet the challenges of rapid evaluation.

The development of new, rapid methodologies needs to be matched by comparative work on their advantages and disadvantages, such as the impact on uncertainty for decision-makers. Such comparative work will be challenging, adding cost and complexity in a context where commissioners and researchers are seeking to reduce both and will require innovative methodologies, such as testing rapid methods nested within ‘standard’ projects e.g. (104, 106) to maximise efficiency.

For peer review only - http://bmjopen.bmj.com/site/about/guidelines.xhtml

Declarations

Ethics approval and consent to participate

This is a scoping review of previously published studies so ethical approval and consent to participate are not applicable.

Consent for publication

Not applicable

Availability of data and materials

This is a scoping review of previously published studies, so all the data used in the review is already in the public domain. Full lists of all studies considered after initial screening are available from the corresponding author on reasonable request, together with the dataset generated by the review process. Studies whose data substantially contributed to the review are cited in the bibliography.

Competing interests

The authors declare that they have no competing interests.

Funding

This research was funded by the National Institute for Health and Care Research Applied Research Collaboration Greater Manchester; grant award NIHR200174. The views expressed in this publication are those of the author(s) and not necessarily those of the National Institute for Health and Care Research or the Department of Health and Social Care.

Author contributions

GN and TM carried out the review process with input from JD, PB, PW and NC. All authors contributed to interpretation of the data. GN wrote the first draft of the manuscript text. JD, TM, PB, PW and NC reviewed and substantially edited and revised the manuscript.

Acknowledgments

The authors are grateful to Dr Su Golder for design and implementation of the search strategy.

For peer review only - http://bmjopen.bmj.com/site/about/guidelines.xhtml

REFERENCES

1. Greenhalgh T, Robert G, MacFarlane F, Bate P, Kyriakidou O. Diffusion of Innovations in Service Organizations: Systematic Review and Recommendations The Milbank Quarterly. 2004;82(4):581-629.
2. Johnson GA, Vindrola-Padros C. Rapid qualitative research methods during complex health emergencies: A systematic review of the literature. Soc Sci Med. 2017;189:63-75.
3. Vindrola-Padros C. Can We Re-Imagine Research So It Is Timely, Relevant and Responsive? Comment on "Experience of Health Leadership in Partnering with University-Based Researchers in Canada: A Call to 'Re-Imagine' Research". International Journal of Health Policy and Management. 2021;10(3):172-5.
4. vindrola-Padros C, Johnson GA. Rapid Techniques in Qualitative Research: A Critical Review of the Literature. . Qualitative Health Research. 2020;30(10):1596-604.
5. Tricco AC, Antony J, Zarin W, Strifler L, Ghassemi M, Ivory J, et al. A scoping review of rapid review methods. BMC Medicine. 2015;13 (1) (no pagination)(224).
6. Tricco AC, Zarin W, Antony J, Hutton B, Moher D, Sherifali D, et al. An international survey and modified Delphi approach revealed numerous rapid review methods. Journal of Clinical Epidemiology. 2016;70:61-7.
7. Cooper K, Martyn-St James M, Pandor A, Kaltenthaler E, Wong R. The impact of searching fewer databases in health technology assessment rapid reviews. International Journal of Technology Assessment in Health Care. 2017;33 (Supplement 1):49-50.
8. Cooper K, Martyn-St James M, Pandor A, Wong R, Kaltenthaler E. Selecting rapid review methods for health technology assessment. International Journal of Technology Assessment in Health Care. 2017;33 (Supplement 1):225-6.
9. Patnode CD, Eder ML, Walsh ES, Viswanathan M, Lin JS. The Use of Rapid Review Methods for the U.S. Preventive Services Task Force. American Journal of Preventive Medicine. 2018;54(1 Supplement 1):S19-S25.
10. Norman G, Dumville JC, Wilson P, Cullum NA, Mason T, Bower P. Scoping review of rapid evaluations https://osf.io/np72f/: Open Science Framework 2020 [
11. Tricco AC, Lillie E, Zarin W, O'Brien KK, Colquhoun H, Levac D, et al. PRISMA Extension for Scoping Reviews (PRISMA-ScR): Checklist and Explanation. Ann Intern Med. 2018;169(7):467-73.
12. WorldBank. The world by income and region https://datatopics.worldbank.org/world-development-indicators/the-world-by-income-and-region.html: The World Bank; 2020 [
13. Team TE. EndNote. Endnote 20 ed. Philadelphia, PA: Clarivate; 2013.
14. Arksey H, O'Malley L. Scoping studies: towards a methodological framework. International Journal of Social Research Methodology. 2005;8(1):19-32.
15. Levac D, Colquhoun H, O'Brien K. Scoping studies: advancing the methodology. Implement Sci. 2010;5:69.
16. Hoffmann TC, Glasziou PP, Boutron I, Milne R, Perera R, Moher D, et al. Better reporting of interventions: template for intervention description and replication (TIDieR) checklist and guide. BMJ : British Medical Journal. 2014;348:g1687.
17. Fergus CJ, Chinn DJ, Murray SA. Assessing and improving out-of-hours palliative care in a deprived community: a rapid appraisal study. Palliative Medicine. 2010;24(5):493-500.
18. Kieber-Emmons A, Crabtree BF, Miller W. A new mixed methods approach to uncover multi-level barriers and facilitators of cancer survivorship. Journal of Clinical Oncology Conference. 2018;36(7).
19. Wright A, Sittig DF, Ash JS, Erickson JL, Hickman TT, Paterno M, et al. Lessons learned from implementing service-oriented clinical decision support at four sites: A qualitative study. International Journal of Medical Informatics. 2015;84(11):901-11.
20. Manderson L, Aaby P. An epidemic in the field? Rapid assessment procedures and health research. Social Science and Medicine. 1992;35(7):839-50.

For peer review only - http://bmjopen.bmj.com/site/about/guidelines.xhtml

21. McMullen CK, Ash JS, Sittig DF, Bunce A, Guappone K, Dykstra R, et al. Rapid assessment of clinical information systems in the healthcare setting: an efficient method for time-pressed evaluation. Methods of information in medicine. 2011;50(4):299-307.
22. Needle RH, Trotter IRT, Goosby E, Bates C, Von Zinkernagel D. Methodologically sound rapid assessment and response: Providing timely data for policy development on drug use interventions and HIV prevention. International Journal of Drug Policy. 2000;11(1-2):19-23.
23. Needle RH, Trotter RT, 2nd, Singer M, Bates C, Page JB, Metzger D, et al. Rapid assessment of the HIV/AIDS crisis in racial and ethnic minority communities: an approach for timely community interventions. American Journal of Public Health. 2003;93(6):970-9.
24. Holdsworth LM, Safaeinili N, Winget M, Lorenz KA, Lough M, Asch S, et al. Adapting rapid assessment procedures for implementation research using a team-based approach to analysis: a case example of patient quality and safety interventions in the ICU. Implement Sci. 2020;15(1):12.
25. Peiro R. Rapid appraisal methodology for 'health for all' policy formulation analysis. 2003.
26. Peiro R, Alvarez-Dardet C, Plasencia A, Borrell C, Colomer C, Moya C, et al. Rapid appraisal methodology for 'health for all' policy formulation analysis. Health Policy. 2002;62(3):309-28.
27. Fitch C, Rhodes T, Stimson GV. Origins of an epidemic: The methodological and political emergence of rapid assessment. International Journal of Drug Policy. 2000;11(1-2):63-82.
28. Stimson GV, Fitch C, DesJarlais D, Poznyak V, Perlis T, Oppenheimer E, et al. Rapid assessment and response studies of injection drug use: knowledge gain, capacity building, and intervention development in a multisite study. American Journal of Public Health. 2006;96(2):288-95.
29. Fitch C, Stimson GV, Rhodes T, Poznyak V. Rapid assessment: an international review of diffusion, practice and outcomes in the substance use field. Soc Sci Med. 2004;59(9):1819-30.
30. Rhodes T, Stimson GV, Fitch C, Ball A, Renton A. Rapid assessment, injecting drug use, and public health. The Lancet. 1999;354(9172):65-8.
31. Campbell J. A critical appraisal of participatory methods in development research. International Journal of Social Research Methodology. 2002;5(1):19-29.
32. Dale J, Shipman C, Lacock L, Davies M. Creating a shared vision of out of hours care: using rapid appraisal methods to create an interagency, community oriented, approach to service development. Bmj. 1996;312(7040):1206-10.
33. Murray SA. Listening to local voices : adapting rapid appraisal to assess health and social needs in general practice. 1994.
34. Murray SA, Tapson J, Turnbull L, McCallum J, Little A. Listening to local voices: Adapting rapid appraisal to assess health and social needs in general practice. British Medical Journal. 1994;308(6930):698-700.
35. Harvey HD, Fleming P, Patterson M. A rapid appraisal method for reviewing the effectiveness of workplace smoking policies in large and medium sized organisations. Journal of The Royal Society for the Promotion of Health. 2001;121(1):50-5.
36. Harvey HD, Fleming P. A rapid appraisal method for the selection and pre-testing of environmental health leaflets. Journal of The Royal Society for the Promotion of Health. 2000;120(2):112-6.
37. Green J, Perkins C, Steinbach R, Edwards P. Reduced street lighting at night and health: A rapid appraisal of public views in England and Wales. Health Place. 2015;34:171-80.
38. Beebe J, Given L. Rapid assessment process. The sage encyclopedia of qualitative research methods

Thousand Oaks, California: SAGE Publications; 2012.
39. Rowa-Dewar N, Ager W, Ryan K, Hargan I, Hubbard G, Kearney N. Using a rapid appraisal approach in a nationwide, multisite public involvement study in Scotland. Qualitative Health Research. 2008;18(6):863-9.

For peer review only - http://bmjopen.bmj.com/site/about/guidelines.xhtml

40. Murphy A, Chikovani I, Uchaneishvili M, Makhashvili N, Roberts B. Barriers to mental health care utilization among internally displaced persons in the republic of Georgia: a rapid appraisal study. BMC health services research. 2018;18(1):306.
41. Foreman-Mackey A, Bayoumi AM, Miskovic M, Kolla G, Strike C. 'It's our safe sanctuary': Experiences of using an unsanctioned overdose prevention site in Toronto, Ontario. International Journal of Drug Policy. 2019;73:135-40.
42. Guise A, Witzel TC, Mandal S, Sabin C, Rhodes T, Nardone A, et al. A qualitative assessment of the acceptability of hepatitis C remote self-testing and self-sampling amongst people who use drugs in London, UK. BMC Infectious Diseases. 2018;18 (1) (no pagination)(281).
43. Phillips CB, Dwan K, Hepworth J, Pearce C, Hall S. Using qualitative mixed methods to study small health care organizations while maximising trustworthiness and authenticity. BMC Health Services Research. 2014;14:559.
44. Williams SP, Kinsey J, Carry MG, Terry L, Wells J, Kroeger K. Get In, Get Tested, Get Care: STD Services in Urban Urgent Care Centers. Sexually Transmitted Diseases. 2019;46(10):648-53.
45. Hamilton S. Felt needs assessment of mental health (rapid service appraisal): Barking and Havering. (Public health research report, ISSN 09689818 ; no 11). 1993:31.
46. Aspray TJ, Nesbit K, Cassidy TP, Hawthorne G. Rapid assessment methods used for health-equity audit: Diabetes mellitus among frail British care-home residents. Public Health. 2006;120(11):1042-51.
47. Livorsi D, Knobloch MJ, Blue LA, Swafford K, Maze L, Riggins K, et al. A rapid assessment of barriers and facilitators to safety culture in an intensive care unit. Int Nurs Rev. 2016;63(3):372-6.
48. Solomon P, Tennille J, Lipsitt D, Plumb E, Metzger D, Blank MB. Rapid assessment of existing HIV prevention programming in a Community Mental Health Center. Journal of Prevention and Intervention in the Community. 2006;33(1-2):137-51.
49. Dupont HB, Kaplan CD, Braam RV, Verbraeck HT, de Vries NK. The application of the rapid assessment and response methodology for cannabis prevention research among youth in the Netherlands. International Journal of Drug Policy. 2015;26(8):731-8.
50. Ong BN. Rapid appraisal and health policy. 1996.
51. Ong BN, Humphris G, Annett H, Rifkin S. Rapid appraisal in an urban setting, an example from the developed world. Soc Sci Med. 1991;32(8):909-15.
52. Organization WH, UNAIDS. SEX-RAR Guide. https://apps.who.int/iris/bitstream/handle/10665/42681/9241545585.pdf?sequence=1&isAllowed=y2002.
53. Trotter IRT, Needle R, Goosby E, Bates C, Singer M. A Methodological Model for Rapid Assessment, Response, and Evaluation: The RARE Program in Public Health. Field Methods. 2001;13(2):137-59.
54. Valderrama JC, Tortajada S, Alapont L, Vidal A, Perez Ma J, Castellano M, et al. Rapid assessment of drug abuse prevention needs for youth in small Spanish municipalities: Coping with resource limitations. Journal of Drug Issues. 2006;36(1):29-52.
55. Brown DR, Hernandez A, Saint-Jean G, Evans S, Tafari I, Brewster LG, et al. A participatory action research pilot study of urban health disparities using rapid assessment response and evaluation. American Journal of Public Health. 2008;98(1):28-38.
56. Burks DJ, Robbins R, Durtschi JP. American Indian gay, bisexual and two-spirit men: a rapid assessment of HIV/AIDS risk factors, barriers to prevention and culturally-sensitive intervention. Culture, health & sexuality. 2011;13(3):283-98.
57. Beebe J. Rapid assessment and response: Sound methodology for producing timely responses. International Journal of Drug Policy. 2000;11(1-2):29-31.
58. Brown CS, Lloyd S, Murray SA. Using consecutive Rapid Participatory Appraisal studies to assess, facilitate and evaluate health and social change in community settings. BMC Public Health. 2006;6 (no pagination)(68).

For peer review only - http://bmjopen.bmj.com/site/about/guidelines.xhtml

59. Lazenbatt A, Lynch U, O'Neill E. Revealing the hidden 'troubles' in Northern Ireland: The role of participatory rapid appraisal. Health Education Research. 2001;16(5):567-78.
60. Annett H. Guidelines for rapid participatory appraisals to assess community health needs: a focus on health improvements for low-income urban and rural areas. Geneva: Division of Strengthening of Health Services World Health Organization available from HMSO; 1995. iv,60 p.
61. Lawlor D. Rapid participatory appraisal of young people's sexual health needs: an evaluation of meta-planning. 1999.
62. Murray SA. Experiences with "rapid appraisal" in primary care: involving the public in assessing health needs, orientating staff, and educating medical students. Bmj. 1999;318(7181):440-4.
63. Sperber NR, Bruening RA, Choate A, Mahanna E, Wang V, Powell BJ, et al. Implementing a Mandated Program Across a Regional Health Care System: A Rapid Qualitative Assessment to Evaluate Early Implementation Strategies. Quality management in health care. 2019;28(3):147-54.
64. Hanckel B, Ruta D, Scott G, Peacock JL, Green J. The Daily Mile as a public health intervention: a rapid ethnographic assessment of uptake and implementation in South London, UK. BMC Public Health. 2019;19(1):1167.
65. Boyd J, Collins AB, Mayer S, Maher L, Kerr T, McNeil R. Gendered violence and overdose prevention sites: a rapid ethnographic study during an overdose epidemic in Vancouver, Canada. Addiction. 2018;113(12):2261-70.
66. Goepp JG, Meykler S, Mooney NE, Lyon C, Raso R, Julliard K. Provider insights about palliative care barriers and facilitators: results of a rapid ethnographic assessment. Am J Hosp Palliat Care. 2008;25(4):309-14.
67. Green TC, Zaller N, Palacios WR, Bowman SE, Ray M, Heimer R, et al. Law enforcement attitudes toward overdose prevention and response. Drug Alcohol Depend. 2013;133(2):677-84.
68. Quintero Romero S, Bernal R, Barbiero C, Passamonte R, Cattaneo A. A rapid ethnographic study of breastfeeding in the North and South of Italy. Int Breastfeed J. 2006;1:14.
69. Kroeger K, Willilams S, Alexander-Pender C, Ford J, Vasani V. "Just one thing on the plate" - Views of community members and providers on sexual health services in the fort mcpherson area of Southwest Atlanta: Findings from a rapid ethnographic assessment. Sexually Transmitted Infections Conference: STI and AIDS World Congress. 2013;89(SUPPL. 1).
70. Palinkas LA, Mendon SJ, Hamilton AB. Innovations in Mixed Methods Evaluations. Annual Review of Public Health. 2019;40:423-42.
71. Palinkas LA, Zatzick D. Rapid Assessment Procedure Informed Clinical Ethnography (RAPICE) in Pragmatic Clinical Trials of Mental Health Services Implementation: Methods and Applied Case Study. Adm Policy Ment Health. 2019;46(2):255-70.
72. Desai A, Donetto S. Evolving ethnographic sensibilities: Using actor-network theory in health services research. BMJ Open. 2019;9 (Supplement 1):A5.
73. Vindrola-Padros C, Vindrola-Padros B. Quick and dirty? A systematic review of the use of rapid ethnographies in healthcare organisation and delivery. BMJ Qual Saf. 2018;27(4):321-30.
74. Keith RE, Crosson JC, O'Malley AS, Cromp D, Taylor EF. Using the Consolidated Framework for Implementation Research (CFIR) to produce actionable findings: a rapid-cycle evaluation approach to improving implementation. Implement Sci. 2017;12(1):15.
75. Shrank WH. The Center For Medicare And Medicaid Innovation's blueprint for rapid-cycle evaluation of new care and payment models. Health Affairs (Millwood). 2013;32(4):807-12.
76. Schneeweiss S, Shrank WH, Ruhl M, Maclure M. Decision-making aligned with rapid-cycle evaluation in health care. International Journal of Technology Assessment in Health Care. 2015;31(4):214-22.
77. Farrer K, Melia D, Blackett B, Lloyd H, Beadman C, Donaldson E, et al. Nutritional screening of elderly patients-a health improvement approach to practice. Proceedings of the Nutrition Society. 2011;70 (OCE5):E317.

For peer review only - http://bmjopen.bmj.com/site/about/guidelines.xhtml

78. Farrer K, Donaldson E, Blackett B, Lloyd H, Forde C, Melia D, et al. Nutritional screening of elderly patients: a health improvement approach to practice. J Hum Nutr Diet. 2014;27(2):184-91.
79. Dunet DO, Sparling PB, Hersey J, Williams-Piehota P, Hill MD, Hanssen C, et al. A new evaluation tool to obtain practice-based evidence of worksite health promotion programs. Preventing chronic disease. 2008;5(4):A118.
80. Gray A, Baker K. Assessment for change. Acute Medicine. 2017;16 (3):127-8.
81. Kercsmar CM, Beck AF, Sauers-Ford H, Simmons J, Wiener B, Crosby L, et al. Association of an asthma improvement collaborative with health care utilization in medicaid-insured pediatric patients in an urban community. JAMA Pediatrics. 2017;171(11):1072-80.
82. Joseph C, Goddard N. Influenza vaccine uptake in the elderly: Results from a rapid assessment of the effectiveness of new government policy in England for the winters 2000/2001 and 2001/2002. Vaccine. 2003;21(11-12):1137-48.
83. Demeulemeester M, Lavis N, Balthazar Y, Lechien P, Heijmans S. Rapid safety assessment of a seasonal intradermal trivalent influenza vaccine. Hum Vaccin Immunother. 2017;13(4):889-94.
84. Banga S, Chabanon AL, Eymin C, Caroe T, Butler K, Moureau A. Near real-time safety surveillance of three seasonal influenza vaccines in Europe during influenza season 2017/18. Pharmacoepidemiology and Drug Safety. 2018;27 (Supplement 2):396.
85. McCarthy A, McMeekin P, Haining S, Bainbridge L, Laing C, Gray J. Rapid evaluation for health and social care innovations: challenges for "quick wins" using interrupted time series. BMC Health Services Research. 2019;19(1):964.
86. Wichmann O, Stocker P, Poggensee G, Altmann D, Walter D, Hellenbrand W, et al. Pandemic influenza A(H1N1) 2009 breakthrough infections and estimates of vaccine effectiveness in Germany 2009-2010. Eurosurveillance. 2010;15(18):1-4.
87. Hamilton AB. Qualitative Methods in Rapid Turn-Around Health Services Research. Spotlight on Women's Health; https://www.hsrd.research.va.gov/for_researchers/cyber_seminars/archives/780-notes.pdf: VA HSR&D Cyberseminar; 2013.
88. Dunbar MS, Towe VL, Ayer L, Martineau M. Connections to Care (C2C): The Perspectives of Leaders at Community-Based Organizations That Are Integrating Mental Health Supports. Rand health q. 2018;7(4):7.
89. Brown-Johnson C, Lo N, Giannitrapani K, Lowery J, Foglia MB, Walling A, et al. A Rapid Qualitative Synthesis of Insights from the Veterans Administration Life-Sustaining Treatment Decisions Initiative (LSTDI) National Implementation (QI703). Journal of Pain and Symptom Management. 2019;57 (2):457.
90. Hamarman A, Mason P, Kroeger K, Alexander-Pender C. The impact of budget cuts among local STD programs in New Jersey: Results of a rapid ethnographic assessment. Sexually Transmitted Diseases. 2014;1):S93.
91. Loosier PS, Carter MW, Hsu KK, Doshi S, Peterson Maddox BL, Kroeger K, et al. Provision of STD Services in Community Settings After the Loss and Return of State Funding to Support Service Provision: Observations From Select Providers in Massachusetts, 2010 and 2013. Journal of public health management and practice : JPHMP. 2020;26(1):E18-E27.
92. Delaney AE, Dadlez NM, Marshall AC. Alternative approach to pediatric cardiac quality assessment for low-volume centers. Congenital Heart Disease. 2019;14(4):665-70.
93. Gray TA, Dumville JC, Christie J, Cullum NA. Rapid research and implementation priority setting for wound care uncertainties. PLoS ONE. 2017;12(12):e0188958.
94. Newlands RS, Power A, Young L, Watson M. Quality improvement of community pharmacy services: a prioritisation exercise. International Journal of Pharmacy Practice. 2018;26(1):39-48.
95. Khan MA, Barratt MS, Krugman SD, Serwint JR, Dumont-Driscoll M, Investigators C. Variability of the institutional review board process within a national research network. Clin Pediatr (Phila). 2014;53(6):556-60.

For peer review only - http://bmjopen.bmj.com/site/about/guidelines.xhtml

96. Bower P, Howells K, McCorkindale S, Bridges L, Sidaway M. Rapid recruitment of large cohort to support trials in general practice: The role of FARSITE. Trials Conference: 3rd International Clinical Trials Methodology Conference United Kingdom. 2015;16(Supplement 2).
97. Brown JSL, Murphy C, Kelly J, Goldsmith K. How can we successfully recruit depressed people? Lessons learned in recruiting depressed participants to a multi-site trial of a brief depression intervention (the 'CLASSIC' trial). Trials. 2019;20(1):131.
98. Deane AM, Hodgson CL, Young P, Little L, Singh V, Poole A, et al. The rapid and accurate categorisation of critically ill patients (RACE) to identify outcomes of interest for longitudinal studies: a feasibility study. Anaesthesia and intensive care. 2017;45(4):476-84.
99. Whelan AM. Consultation with non-English speaking communities: rapid bilingual appraisal. 2004.
100. Alolod G, Siminoff LA, Traino HM. Usability of the rapid assessment of hospital procurement barriers in donation (RAPID) as a tool for OPO hospital development staff. Transplantation. 2015;2):S47.
101. Alolod GP, Traino HM, Siminoff LA. Utility and Usability of the Rapid Assessment of Hospital Procurement Barriers in Donation (RAPiD) as a Tool for OPO Hospital Development Staff. Prog Transplant. 2016;26(3):241-8.
102. Traino HM, Alolod GP, Shafer T, Siminoff LA. Interim results of a national test of the rapid assessment of hospital procurement barriers in donation (RAPiD). American Journal of Transplantation. 2012;12(11):3094-103.
103. Moodie Z, Janes H, Huang Y. New clinical trial designs for HIV vaccine evaluation. Curr Opin HIV AIDS. 2013;8(5):437-42.
104. Hensel JM, Shaw J, Jeffs L, Ivers NM, Desveaux L, Cohen A, et al. A pragmatic randomized control trial and realist evaluation on the implementation and effectiveness of an internet application to support self-management among individuals seeking specialized mental health care: A study protocol. BMC Psychiatry. 2016;16 (1) (no pagination)(350).
105. Simpson CR, Lone N, McMenamin J, Gunson R, Robertson C, Ritchie LD, et al. Early estimation of pandemic influenza Antiviral and Vaccine Effectiveness (EAVE): use of a unique community and laboratory national data-linked cohort study. Health Technol Assess. 2015;19(79):1-32.
106. Glasgow RE, Kessler RS, Ory MG, Roby D, Gorin SS, Krist A. Conducting rapid, relevant research: Lessons learned from the my own health report project. American Journal of Preventive Medicine. 2014;47(2):212-9.
107. Taylor B, Henshall C, Kenyon S, Litchfield I, Greenfield S. Can rapid approaches to qualitative analysis deliver timely, valid findings to clinical leaders? A mixed methods study comparing rapid and thematic analysis. BMJ Open. 2018;8 (10) (no pagination)(e019993).
108. Burgess-Allen J, Owen-Smith V. Using mind mapping techniques for rapid qualitative data analysis in public participation processes. Health Expect. 2010;13(4):406-15.
109. Begg NT, Gill ON, White JM. COVER (Cover Of Vaccination Evaluated Rapidly): Description of the England and Wales scheme. Public Health. 1989;103(2):81-9.
110. Currow DC, Vella-Brincat J, Fazekas B, Clark K, Doogue M, Rowett D. Pharmacovigilance in hospice/palliative care: Rapid report of net clinical effect of metoclopramide. Journal of Palliative Medicine. 2012;15(10):1071-5.
111. Noack PS, Moore JA, Poon M. Advanced Imaging Reduces Cost Compared to Standard of Care in Emergency Department of Triage of Acute Chest Pain. Health Services Research. 2018;53(4):2384-405.
112. Centers for Disease C, Prevention. Rapid assessment of influenza vaccination coverage among HMO members--northern California influenza seasons, 2001-02 through 2004-05. MMWR Morb Mortal Wkly Rep. 2005;54(27):676-8.

For peer review only - http://bmjopen.bmj.com/site/about/guidelines.xhtml

113. Gagne JJ, Wang S, Schneeweiss S. A multivariable-adjusted rapid assessment of the association between zoledronic acid and myocardial infarction. Pharmacoepidemiology and Drug Safety. 2013;1):378.
114. Dodd CN, de Ridder M, Huang WT, Weibel D, Giner-Soriano M, Perez-Vilar S, et al. Incidence rates of narcolepsy diagnoses in Taiwan, Canada, and Europe: The use of statistical simulation to evaluate methods for the rapid assessment of potential safety issues on a population level in the SOMNIA study. PLoS ONE. 2018;13(10):e0204799.
115. Alguacil-Ramos AM, Portero-Alonso A, Pastor-Villalba E, Muelas-Tirado J, Diez-Domingo J, Sanchis-Ferrer A, et al. Rapid assessment of enhanced safety surveillance for influenza vaccine. Public Health. 2019;168:137-41.
116. Lester C. Priority setting with health inequality impact assessment. 2001.
117. Cakir B, Uner S, Temel F, Akin L. Lot quality survey: An appealing method for rapid evaluation of vaccine coverage in developing countries - Experience in Turkey. BMC Public Health. 2008;8 (no pagination)(240).
118. Akande TM, Eshetu M, Bonsu G. Rapid assessment as an evaluation tool for Polio National Immunisation Days in Brong Ahafo Region, Ghana. Annals of African Medicine. 2005;4(4):172-6.
119. Gagne JJ, Bykov K, Najafzadeh M, Choudhry NK, Martin D, Kahler KH, et al. Prospective benefit-risk monitoring of new drugs for rapid assessment of net favorability in electronic healthcare data. Pharmacoepidemiology and Drug Safety. 2015;1):38-9.
120. Hawley S, Ali MS, Berencsi K, Judge A, Prieto-Alhambra D. Sample size and power considerations for ordinary least squares interrupted time series analysis: a simulation study. . Clinical Epidemiology. 2019;11:197-205.
121. Ramsay CR, Matowe L, Grilli R, Grimshaw JM, Thomas RE. Interrupted time series designs in health technology assessment: lessons from two systematic reviews of behavior change strategies. . International Journal of Technology Assessment in Health Care. 2003;19(4):613-23.
122. Hategeka C, Ruton H, Karamouzian M, Lynd L, Law MR. Use of interrupted time series methods in the evaluation of health system quality improvement interventions: a methodological systematic review. BMJ Global Health. 2020;5:e003567.
123. EPOC. EPOC Resources for Review Authors https://epoc.cochrane.org/resources/epoc-resources-review-authors: Cochrane Collaboration; 2017 [
124. Pradhan S, Deshmukh A, Giri Shrestha P, Basnet P, Kandel RP, Lewallen S, et al. Prevalence of blindness and cataract surgical coverage in Narayani Zone, Nepal: A rapid assessment of avoidable blindness (RAAB) study. British Journal of Ophthalmology. 2018;102(3):291-4.
125. Kengne-Ouafo JA, Nji TM, Tantoh WF, Nyoh DN, Tendongfor N, Enyong PA, et al. Perceptions of consent, permission structures and approaches to the community: a rapid ethical assessment performed in North West Cameroon. BMC Public Health. 2014;14:1026.
126. Abay S, Addissie A, Davey G, Farsides B, Addissie T. Rapid Ethical Assessment on Informed Consent Content and Procedure in Hintalo-Wajirat, Northern Ethiopia: A Qualitative Study. PLoS ONE. 2016;11(6):e0157056.
127. Addissie A, Abay S, Feleke Y, Newport M, Farsides B, Davey G. Cluster randomized trial assessing the effects of rapid ethical assessment on informed consent comprehension in a low-resource setting. BMC medical ethics. 2016;17(1):40.
128. Gebresilase TT, Andargie Z, Tsegay G, Aseffa A, Davey G, Newport M, et al. Using rapid ethical appraisal (REA) to design a contextualized consent process for biomedical studies the Ethiopia. American Journal of Tropical Medicine and Hygiene. 2015;93 (4 Supplement):23.
129. Bull S, Farsides B, Ayele FT. Tailoring information provision and consent processes to research contexts: The value of rapid assessments. Journal of Empirical Research on Human Research Ethics. 2012;7(1):37-52.
130. Feeley N, Cossette S, Côté J, Héon M, Stremler R, Martorella G, et al. The importance of piloting an RCT intervention. Canadian Journal of Nursing Research. 2009;41(2):85-99.

For peer review only - http://bmjopen.bmj.com/site/about/guidelines.xhtml

131. Jones A, Pierce M, Sutton M, Mason T, Millar T. Does paying service providers by results improve recovery outcomes for drug misusers in treatment in England? Addiction. 2018;113(2):279-86.
132. Mason T, Sutton M, Whittaker W, McSweeney T, Millar T, Donmall M, et al. The impact of paying treatment providers for outcomes: difference-in-differences analysis of the 'payment by results for drugs recovery' pilot. Addiction. 2015;110(7):1120-8.
133. Mason T, Whittaker W, Jones A, Sutton M. Did paying drugs misuse treatment providers for outcomes lead to unintended consequences for hospital admissions? Difference-in-differences analysis of a pay-for-performance scheme in England. Addiction. 2021;116(11):3082-93.
134. BRACE. The trade off between rigour and real world evidence needs https://www.birmingham.ac.uk/research/brace/blogs/.aspx: University of Birmingham; 2021 [
135. RECOVERY. RECOVERY. randomised evaluation of covid-19 therapy https://www.recoverytrial.net/2021 [
136. Raj N, Fernandes S, Charyulu N, Dubey A, Ravi GS, Hebbar S. Postmarket surveillance: a review on key aspects and measures on the effective functioning in the context of the United Kingdom and Canada. Therapeutic Advances in Drug Safety. 2019;10:2042098619865413.
137. NICE, Excellence NIfHaC. Evidence standards framework for digital health technologies https://www.nice.org.uk/about/what-we-do/our-programmes/evidence-standards-framework-for-digital-health-technologies: National Institute for Health and Care Excellence; 2021 [

For peer review only

For peer review only - http://bmjopen.bmj.com/site/about/guidelines.xhtml

Figure 1: PRISMA diagram showing study flow for the review

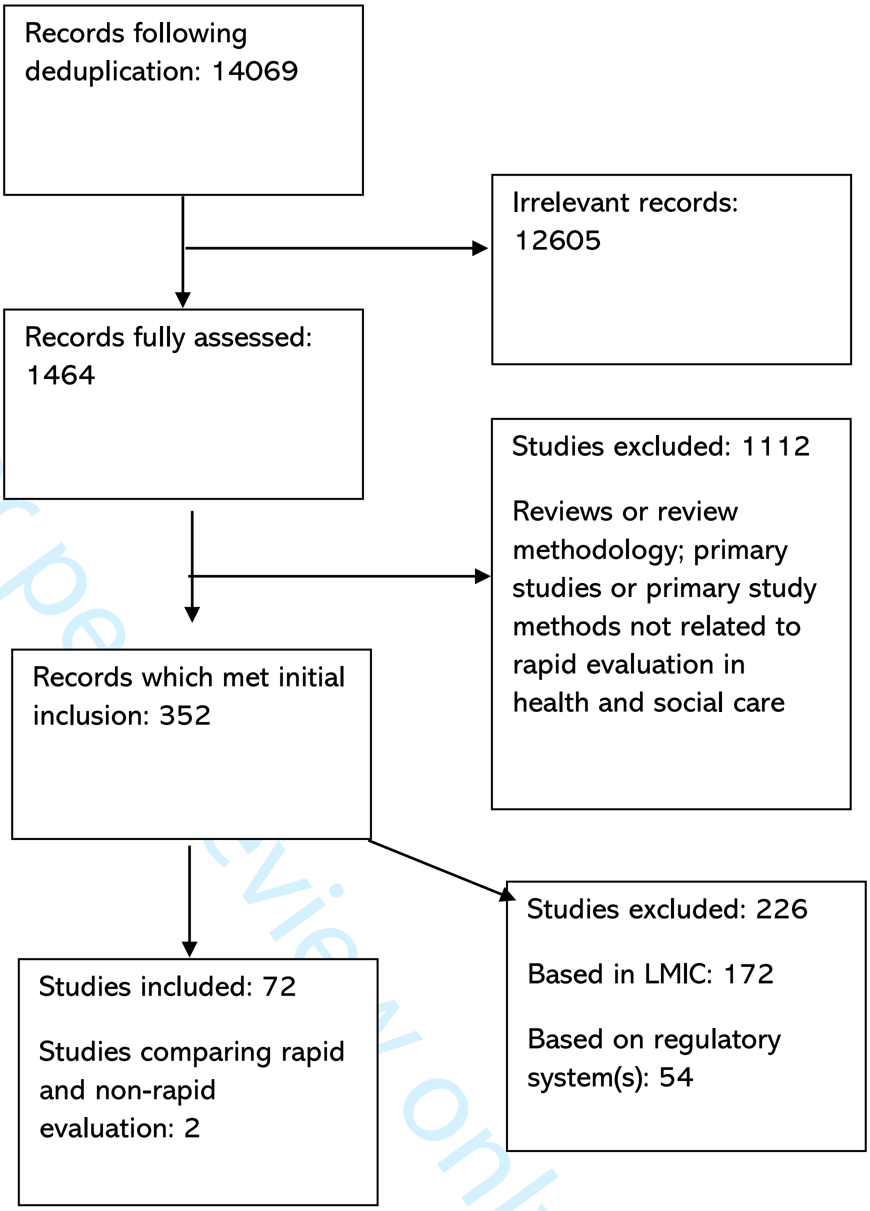

For peer review only

Appendix 1: Medline Search for rapid evaluations

Rapid evaluations

Database: Ovid MEDLINE(R) and Epub Ahead of Print, In-Process & Other Non-Indexed Citations and Daily <1946 to February 24, 2020>
Search Strategy:
----
1 rapid evaluation.tw. (1469)
2 (Rapid adj2 (assessment* or report* or qualitative research or research or studies or study or evaluation* or appraisal* or approach* or response brief* or response program*)).ti. (2911)
3 ((Fast or faster) adj (assessments or reports or qualitative research or research or studies or evaluations or appraisals or approaches or response briefs or response program*)).tw. (87)
4 ((limit* or short* or tight or efficient or pragmatic) adj2 (deadline* or time or timescale or timeline or time period or time frame) adj2 (assessments or reports or qualitative research or research or studies or evaluations or appraisals or approaches or response briefs or response program*)).tw. (275)
5 ((Speed or speedy) adj (assessments or reports or qualitative research or research or studies or evaluations or appraisals or approaches or response briefs or response program*)).tw. (88)
6 (Abbreviated adj (assessments or reports or qualitative research or research or studies or evaluations or appraisals or approaches or response briefs or response program*)).tw. (11)
7 (Accelerated adj (assessments or reports or qualitative research or research or studies or evaluations or appraisals or approaches or response briefs or response program*)).tw. (152)
8 (Brief adj (assessments or reports or qualitative research or research or studies or evaluations or appraisals or approaches or response program*)).tw. (494)
9 (constrained by time adj10 (assessments or reports or qualitative research or research or studies or evaluations or appraisals or approaches or response briefs or response program*)).tw. (3)
10 (Expedited adj (assessments or reports or qualitative research or research or studies or evaluations or appraisals or approaches or response briefs or response program*)).tw. (14)
11 (Express adj (assessments or reports or qualitative research or research or studies or evaluations or appraisals or approaches or response briefs or response program*)).tw. (7)
12 (Immediate adj (qualitative research or research or studies or evaluations or appraisals or approaches or response briefs or response program*)).tw. (61)
13 ((Quick adj2 dirty) and (assessments or reports or qualitative research or research

For peer review only

or studies or evaluations or appraisals or approaches or response briefs or response program*)).tw. (16)

14 (Quick* adj (assessments or reports or qualitative research or research or studies or evaluations or appraisals or approaches or response briefs or response program*)).tw. (71)

15 (Rapid adj (assessments or reports or qualitative research or research or studies or evaluations or appraisals or approaches or response brief* or response program*)).tw. (292)

16 (Stream-line* adj (assessments or reports or qualitative research or research or studies or evaluations or appraisals or approaches or response briefs or response program*)).tw. (1)

17 (Streamline* adj (assessments or reports or qualitative research or research or studies or evaluations or appraisals or approaches or response briefs or response program*)).tw. (59)

18 (timeliness adj6 (assessments or reports or qualitative research or research or studies or evaluations or appraisals or approaches or response briefs or response program*)).tw. (211)

19 (Timely adj (assessments or reports or qualitative research or research or studies or evaluations or appraisals or approaches or response briefs or response program*)).tw. (146)

20 or/1-19 (5967)

21 ((Fast or faster) adj (appraisal or approach or study or assessment or report)).tw. (577)

22 ((limit* or short* or tight or efficient or pragmatic) adj2 (deadline* or time or timescale or timeline or time period or time frame) adj2 (appraisal or approach or study or assessment or report)).tw. (650)

23 ((Speed or speedy) adj (appraisal or approach or study or assessment or report)).tw. (115)

24 (Abbreviated adj (appraisal or approach or study or assessment or report)).tw. (45)

25 (Accelerated adj (appraisal or approach or study or assessment or report)).tw. (79)

26 (Brief adj (appraisal or approach or study or assessment or report)).tw. (6166)

27 (constrained by time adj10 (appraisal or approach or study or assessment or report)).tw. (3)

28 (Expedited adj (appraisal or approach or study or assessment or report)).tw. (21)

29 (Express adj (appraisal or approach or study or assessment or report)).tw. (56)

30 (Immediate adj (appraisal or approach or study or assessment or report)).tw. (376)

31 ((Quick adj2 dirty) and (appraisal or approach or study or assessment or report)).tw. (23)

32 (Quick* adj (appraisal or approach or study or assessment or report)).tw. (433)

33 (Rapid adj (appraisal or approach or study or assessment or report)).tw. (4268)

34 (Stream-line* adj (appraisal or approach or study or assessment or report)).tw. (3)
35 (Streamline* adj (appraisal or approach or study or assessment or report)).tw. (234)
36 (timeliness adj6 (appraisal or approach or study or assessment or report)).tw. (277)
37 (Timely adj (appraisal or approach or study or assessment or report)).tw. (329)
38 or/21-37 (13624)
39 evidence-based practice/ (9666)
40 evidence-based medicine/ (72011)
41 Decision Making/ (92841)
42 exp Research/mt, st, sn, td [Methods, Standards, Statistics & Numerical Data, Trends] (84281)
43 exp Animals/ not (exp Animals/ and Humans/) (4671550)
44 39 or 40 or 41 or 42 (250808)
45 38 and 44 (214)
46 20 or 45 (6162)
47 46 not 43 (5569)
48 47 and 20*.ed. (3932)

Rapid reviews

Database: Ovid MEDLINE(R) and Epub Ahead of Print, In-Process & Other Non-Indexed Citations and Daily <1946 to February 24, 2020>
Search Strategy:
----
1 ((abbreviated adj review?) or (abbreviated adj synthes?s) or (accelerated adj2 review?) or (accelerated adj2 synthes?s) or (brief adj synthes?s) or (expedited adj2 review?) or (expedited adj2 synthes?s) or (meta adj method$) or (meta adj evaluat$) or (rapid adj2 review?) or (rapid adj2 assess$) or "rapid health technology assess$" or (rapid adj HTA?) or (rapid adj approach$) or (rapid adj search$) or (realis$ adj approach$) or (realis$ adj evaluat$) or (realis$ adj synthes?s) or (speed$ adj2 review?) or (streamline$ adj2 review?) or (streamline$ adj2 synthes?s) or (stream-line$ adj2 review?) or (stream-line$ adj2 synthes?s) or (fast$ adj2 review?) or (fast$ adj2 synthes?s)).tw. (9897)
2 (rapid adj2 synthes?s).tw. not (ch or cs).fs. (1152)
3 1 or 2 (11015)
4 exp Animals/ not (exp Animals/ and Humans/) (4671550)
5 3 not 4 (9598)

6 5 and ("2014$" or "2015$" or "2016$" or "2017$" or "2018$" or "2019$" or "2020$").ed. (3345)

For peer review only

Appendix 2: Further information on identified studies

Supplementary table 1: Primary studies in the UK

Study ID	Type of study	Rapid descriptor	Study question	Methods used	Data collected
Aspray 2006(63)	Mixed methods	Rapid evaluation	Equity of care diabetes standards in care homes	Interviews, checklists, record review	Quality of care metrics; experience of patients and carers
Banga 2018(64)	Quantitative	Rapid safety evaluation	Coverage and AE in influenza vaccination	Enhanced passive surveillance, spontaneous reporting, usage data	Uptake and AE (especially reporting within 7 days)
Begg 1989(65)	Quantitative	Rapid evaluation	Vaccination coverage of sentinel vaccines at key timepoints	Analysis of quarterly data cohorts	Proportion of eligible children immunised
Brook 2011(66)	Quantitative	Rapid cycle assessment	Sexual health clinic service evaluation	Electronic patient record analysis	Proportion of people treated and partners tested
Brown 2006(29)	Qualitative	Rapid participatory appraisal	Healthy environment - perceptions of need and concern	Documentation review, observations, interviews	Perceived needs and concerns around health-related needs (environment-based)
Dale 1996(16)	Qualitative	Rapid appraisal	Out of hours primary care strengths and weaknesses	Quantitative and qualitative survey methods	Availability of out of hours care and integration with other services
Farrer 2014(54)	Mixed methods	Rapid test changes	Changes in dietician assessments	Evaluation of ward data records	Use of new tool within designated time frame and accuracy of use
Fergus 2010(1)	Mixed methods	Rapid participatory appraisal	Palliative care out of hours provision	Routine data; semi-structured interviews; direct observations	Service provision and demand for services, barriers to accessing services

For peer review only

Gray 2017(67)	Mixed methods	Initial review	Rapid assessment of patients in acute medical unit	Staff feedback and information distribution	Triage of patients within timeframes, changes in times to assessment etc.
Green 2015(21)	Qualitative	Rapid appraisal	Reduced street lighting at night	Ethnographic data, household survey, document review	Public views on intervention
Guise 2018(58)	Qualitative	Rapid assessment	Remote hepatitis C testing	Focus groups and semi-structured interviews	Acceptability of approach, ease of use, other considerations including result management
Hamilton 1993(23)	Qualitative	Rapid appraisal	Planning for mental health provision	Semi-structured interviews	Service priorities for provision at borough level
Hanckel 2019(34)	Qualitative	Rapid ethnographic assessment	Daily mile in school	Interviews, observations, routine data	Implementation methods and perceived benefits
Joseph 2003(68)	Quantitative	Rapid reporting	Influenza vaccination	Data collection from records	Immunization uptake among population aged 65+
Lazenbatt 2001 (28)	Mixed methods	Rapid participatory appraisal	Women's access to health service provision	Overview of provision	Access to health services and related barriers
Lawlor 1999(31)	Qualitative	Rapid participatory appraisal	Sexual health needs in young people	Meta-planning	Needs in sexual health services planning
Lester 2002(69)	Mixed methods	Health inequality impact rapid appraisal	Impact of health inequalities on community and services	Complex multidisciplinary approaches	Cycle of deprivation – methods to break this
Newlands 2018(70)	Mixed methods	Rapid approach to knowledge translation	Quality improvement of pharmacy services	Nominal group technique, Delphi process	Priorities for knowledge translation into practice

For peer review only

Rowa-Dewar 2008(24)	Qualitative	Rapid appraisal approach	Public perceptions of cancer services	Multisite rapid appraisals in communities including those hard to reach	Views of cancer care in communities considered hard to reach

Supplementary Table 2: Primary studies in non-UK high income countries

Study ID	Country	Type of study	Rapid descriptor	Study question	Methods used	Data collected
Alguacil-Ramos 2019(71)	Spain	Quantitative	Rapid detection of safety signals	Adverse events of influenza vaccination	Population based register, real-time consultation	Specific adverse events, demographics impacted
Baum 1995(72)	USA	Quantitative	Rapid technology assessment	Magnetic resonance angiography	RCT of test and treat	Sensitivity and specificity
Boyd 2018(35)	Canada	Qualitative	Rapid ethnographic study	Supervised consumption sites for drug overdose preventions	Observations and interviews	Experiences of participants
Brown 2008(46)	USA	Mixed methods	Rapid assessment response and evaluation	Guiding interventions for urban health disparities	Mapping, observations, interviews, focus groups	Care received by key groups
Brown-Johnson 2019(73)	USA	Qualitative	Lightning report rapid qualitative synthesis	Barriers and facilitators of life-sustaining treatment decisions initiative	Interviews	Provision

For peer review only

Burks 2011(47)	USA	Qualitative	Rapid assessment	Barriers to prevention and treatment of HIV	Multiple triangulated methods with high user involvement	Barriers to accessing care
Cakir 2008(74)	Turkey	Quantitative	Rapid evaluation	Routine vaccination coverage	Survey, interviews	Vaccines received, reasons for non-vaccination
CDC 2005(75)	USA	Quantitative	Rapid assessment	Vaccination coverage	Automated rapid analysis system	Adverse events, vaccination coverage, adherence to guidelines
Currow 2012(76)	Australia & New Zealand	Quantitative	Rapid report	Metoclopramide	Data on use and symptoms in cohort	Benefit and adverse events, treatment adherence
D'Amore 2003(77)	USA	Quantitative	Brief report	Automatic therapeutic interchange programme	Pharmacy records review	Impact on outpatient therapy
Delaney 2019(78)	USA	Mixed	Rapid approach assessment	Low volume paediatric cardiac care programmes	Retrospective chart review	Quality of care metrics
Demeulemeester 2017(79)	Belgium	Quantitative	Rapid safety assessment	Seasonal influenza vaccination	Vaccination surveillance	Specified and unspecified adverse events
Dunbar 2018(80)	USA	Mixed methods	Brief research study	Connections to care programme	Interviews	effect on clients, government and community organisation spending
Dupont 2015(41)	Netherlands	Qualitative	Rapid assessment and response	Evidence based interventions for young people	Stepwise fieldwork	Use and patterns of use of cannabis

For peer review only

Foreman-Mackey 2019 (26)	Canada	Quantitative	Rapid evaluation	Overdose prevention sites	Semi-structured interviews	Experiences of people accessing smoking and injection services
Gagne 2013(81)	USA	Quantitative	Rapid assessment	Zoledronic acid adverse event	Analysis of data from commercial claims database	Myocardial infarctions in people taking zoledronic acid vs other bisphosphonates
Goepp 2008(36)	USA	Qualitative	Rapid assessment	Barriers and facilitators to inpatient palliative care	Interviews	factors influencing service uptake
Green 2013 (37)	USA	Qualitative	Rapid assessment	law enforcement overdose prevention	Interviews	Experiences of overdose prevention and naloxone
Hamarman 2014 (82)	USA	Qualitative	Rapid assessment	Sexually transmitted disease programmes	Interviews and observations	Service provision; integration and collaboration
Hensel 2016(83)	Canada	Mixed methods	Pragmatic randomised trial	Online platform for mental health self-management	Data analysis of trial outcomes; participant interviews	Recovery at 3 months
Kercsmar 2017 (53)	USA	Quantitative	Rapid evaluation	Paediatric asthma care redesign	Control charts (annotated)	Hospitalisations and ED visits
Kieber-Emmons 2018(2)	USA	Mixed methods	Rapid assessment	Care for cancer patients/survivors	Geographic information systems, observations, interviews	Mortality rates, insurance coverage, provision of care, delivery methods
Kroeger 2013 (40)	USA	Mixed methods	Rapid ethnographic assessment	Sexual health services	Interviews	Views on available services/potential changes to services

For peer review only

Livorsi 2016 (84)	USA	Mixed methods	Rapid assessment	Barriers and facilitators to safety culture in an intensive care unit	Interviews	Reporting errors, approachability of authority figures and handovers.
Loosier 2020 (85)	USA	Qualitative	Rapid ethnographic assessment	Sexual health services	Interviews	Financial and care capability parameters
MacCarthy 2019 (86)	USA	Qualitative	Rapid assessment process	HIV prevention information	Interviews	HIV prevention information on mobile technology
Murphy 2018 (25)	Georgia	Mixed methods	Rapid appraisal	Barriers to mental health services	Document review; semi-structured interviews, triangulation	Barriers to access for internally displaced populations
Quintero Romero 2006 (39)	Italy	Mixed methods	Rapid ethnographic study	Information and support for breastfeeding	Interviews	Access to and need for support, experiences of services
Seay 2017(87)	USA	Quantitative	Rapid assessment	Transgender experience and use of cervical screening	Survey	Uptake, causes of low uptake
Seay 2015(88)	USA	Quantitative	Rapid assessment	cancer screening in immigrant communities	Survey	Uptake and access disparities
Solomon 2006(89)	USA	Qualitative	Rapid assessment procedure	HIV prevention programming in Community Mental Health Centre	Interviews, focus groups, ecological assessment	Availability and perception of availability of services and materials

For peer review only

Sperber 2019(49)	USA	Qualitative	Rapid qualitative assessment	Walking programme for hospitalised older adults	interview, document review	Impacts of early stage implementation
Valderrama 2006 (48)	Spain	Qualitative	Rapid assessment	Drug abuse prevention needs of young people	Interviews (from rapid assessment and response evaluation methods)	Coverage of mandated prevention needs
Wichmann 2010 (90)	Germany	Quantitative	Rapid assessment	Pandemic influenza vaccination effectiveness	Data records of influenza cases vaccination status	Effectiveness estimates – breakthrough infections
Williams 2019 (91)	USA	Mixed methods	Rapid gap assessment	Sexual health services coverage	Site visits, checklists, online data sources, open-ended questioning	Service provision in urgent care centres; challenges experienced
Wiss 2019 (92)	USA	Quantitative	Rapid assessment	nutritional services in drugs misuse treatment services	Survey	Service provision and characteristics

For peer review only

Supplementary table 3: Studies assessing implementation in high income countries

Study ID	Country	Type of study	Rapid descriptor	Study question	Results
Ash 2008 (93)	USA	Mixed methods	Rapid assessment process	Informatics in clinical decision support	implementation for rapid assessment process and actionable feedback
Belford 2017 (94)	UK	Mixed methods	Evaluability assessment giving quick feedback	Community development programme to reduce health inequality	Stakeholder involvement to produce logic model of programme theory and evaluation opinions
Boucaud-Maitre 2016 (95)	France	Quantitative	Accelerated assessment	Accelerated approval process for medicines	Subsequent HTA assessment of therapeutic value
Haigh 2013 (96)	Australia and New Zealand	Quantitative	Rapid health impact assessment	Use of health impact reports including rapid assessments	Quality assessment of reports to develop improvements in practice
Hailey 2001 (97)	Canada	Quantitative	Rapid assessment process	Rapid HTAs	Rapid HTA programme in provincial healthcare system in response to urgent requests for advice
Holdsworth 2020 (8)	USA	Mixed methods	Rapid assessment	ICU redesign for patient safety	Implementation methods and perceived benefits; barriers and facilitators
Keith 2017 (50)	USA	Qualitative	Rapid cycle evaluation	Barriers and facilitators to different comprehensive primary care initiative components	Consolidated framework for implementation research useful for rapid cycle evaluation of practice transformation initiatives

For peer review only

Sperber 2019 (49)	USA	Qualitative	Rapid qualitative assessment	Early strategies to implement evidence-based walking program for hospitalized older adults	Lessons from early stage implementation
Wright 2015 (3)	USA	Qualitative	Rapid assessment process	Best practice for a clinical decision support system	Challenges and lessons for implementation

For peer review only

Supplementary Table 4: Descriptions for methodologies described as specifically designed to enable rapid evaluation

Name of methodology	Description of methods	Example(s)
Rapid assessment/appraisal procedure/process (RAP) [multiple terms used for variations and adaptations of core methods.]	Exemplifies use of triangulation of multiple data sources (e.g. interviews and surveys with or without additional data sources). Multiple adaptations identified. Use focused on provision and delivery, barriers, facilitators and perceptions of benefit.	Fergus 2010 (1)(adaptation) Kieber-Emmons 2018 (adapted) (2); Wright 2015; (3) Manderson 1992;(4) McMullen 2011(5) (for clinical informatics); Needle 2000/3(6, 7)<br>Adapted for use in evaluation of changes. Holdsworth(8)<br>Used for policy formulation. Peiro(9, 10)<br>Fitch 2000; 2004 Stimson 2006(11-13)<br>Limitations(14, 15)
Rapid appraisal [term used for variations and adaptations of core methods]	Interviews with range of individuals, documentary evidence, focused ethnographic visits, survey; thematic analysis. Not all methods used in all iterations. Related to methods of rapid assessment and response and rapid assessment, response and evaluation.(16) (17, 18) (19, 20)<br>Use focused on views or assessments of policies or services.	Green 2015(21) (refs Beebe 2012) (22) Hamilton 1993(23) Rowa-dewar 2008 (24) Harvey 2001(19, 20) Murphy 2018(25)
Rapid evaluation approach of an emergent program	Semi-structured interviews with multiple types of stakeholders; triangulation of data from different stakeholders using same methods; thematic analysis. Focus on service user experiences.	Foreman-Mackey 2019(26, 27) (refs Beebe 2012 (22))
Rapid participatory appraisal	Adaptation of rapid appraisal to involve community in design of appraisal study; includes use of consecutive studies in same community. Use focused on access (and barriers to access) to services; perspectives, experiences and satisfaction relating to services.	Brown 2006 (28) Lazenblatt 2001 (29) Murray 1994, 1999(17, 18), Annett 1995 (guidance) (30)

For peer review only

Rapid participatory appraisal with meta-planning	Uses peer facilitation of data collection; triangulates two separate groups of participant responses.	Lawlor 1999 (31)
Rapid ethnographic assessment/appraisal /rapid ethnography	Assessment of intervention in naturalistic setting. Interviews, focus groups, observations of implementation, analysis of routine data sources (not all methods in all studies). Typically used interview and survey data, triangulated. Includes but is not limited to evaluation of services; focus on views and experiences. Question mark over whether truly ethnographic. (32, 33)	Hanckel 2019(34-37) Boyd 2018 (35) Goepp 2008,(36) Green 2013(37) Ackerman 2017(38) Quintero-Romero;(39) Kroeger 2013(40)
Rapid assessment and response (RAR)	Interviews with representative individuals/ groups (also described as focus groups), integrates findings with data from existing records and desk research; researcher training required for effectiveness. Focus on knowledge and service capacity and development	Dupont 2015;(41) Stimson 2006(13) Ong 1996, 1991(42, 43) WHO 2002 (guidance for psychoactive drugs and sexual risk behaviours) (44)
Rapid assessment, response and evaluation (RARE)	A model for using RAR within context of public health policy development; (45) RAR in implementation evaluation. Substantive training component for proper implementation. Rapid qualitative ethnographic methods: use of existing data; direct involvement of community leaders/health providers; explicit evaluation component. Focus on care coverage, access and barriers.	Trotter 2001(45) Needle 2000,2003(6, 7) Brown 2008(46)Burks 2011(47) Valderrama 2006 (48)(combined with policy level surveying)
Rapid cycle evaluation	Use of consolidated framework for implementation research (CFIR) using observational field notes and semi-structured interviews across multiple sites to enable rapid cycle data analysis and generate actionable findings during the research project life cycle. Using Expert recommendations for	Keith 2017 (Shrank 2013)(50, 51)

For peer review only

	implementing change (ERIC) (27, 49) Identifies barriers and facilitators to components of initiatives.	
Plan-do-study-act cycles	Method drawn from quality improvement. Small-scale implementation and evaluation of changes, with weekly or monthly (e.g.) tracking of routinely collected data at ward/hospital/district level. Shares some features with studies using real-time feedback in other designs.(52)	Farrer 2014; Kercsmar 2017 (53, 54)
Rapid cycle evaluation building in plan do study act (RAPID)	Method to support decision making based on rapid cycle evaluation of electronic data	Schneeweiss 2015(55)
Rapid assessment procedure informed clinical ethnography (RAPICE)	Mixed methods approach using a combination of rapid appraisal and ethnography to understand implementation processes. Linked to a pragmatic trial; focused on barriers and facilitators.	Palinkas 2019a/b (56, 57)
Qualitative rapid assessment	Focus groups and semi-structured interviews; thematic analysis; focus on acceptability and feasibility.	Guise 2018(58)
Qualitative rapid appraisal, Rigorous analysis (Q-RARA)	Qualitative mixed methods: site visit, semi-structured interviews, observations, photographs, floor plans, and social scanning data; integrated qualitative analysis approach. Focus on organisational function and processes in small primary health care organisations.	Phillips 2014(59)
Rapid gap assessment approach	Mixed methods involving targeted site identification; checklist-based data collection; notes from participating centres and open ended questions; use of online search methods to identify sites. Approach described as modified rapid gap assessment.	Williams 2019(60)
Swift Worksite Assessment and Translation (SWAT)	Multiple evaluation approaches – site visits, interviews etc. Includes disseminations stage; aims to assess effectiveness of	Dunet 2008(61)

For peer review only

	workplace health policies may be suitable for innovations aimed at HCP	
Pilot implementation	Although we did not include studies described purely as pilots this represented a rapid assessment of implementation of change – assessment undertaken after one week. Similar to plan-do-study-act cycle approaches. Focus on measures of target meeting for triage time.	Gray 2017(62)

For peer review only

References for supplementary material

1. Fergus CJ, Chinn DJ, Murray SA. Assessing and improving out-of-hours palliative care in a deprived community: a rapid appraisal study. Palliative Medicine. 2010;24(5):493-500.
2. Kieber-Emmons A, Crabtree BF, Miller W. A new mixed methods approach to uncover multi-level barriers and facilitators of cancer survivorship. Journal of Clinical Oncology Conference. 2018;36(7).
3. Wright A, Sittig DF, Ash JS, Erickson JL, Hickman TT, Paterno M, et al. Lessons learned from implementing service-oriented clinical decision support at four sites: A qualitative study. International Journal of Medical Informatics. 2015;84(11):901-11.
4. Manderson L, Aaby P. An epidemic in the field? Rapid assessment procedures and health research. Social Science and Medicine. 1992;35(7):839-50.
5. McMullen CK, Ash JS, Sittig DF, Bunce A, Guappone K, Dykstra R, et al. Rapid assessment of clinical information systems in the healthcare setting: an efficient method for time-pressed evaluation. Methods of information in medicine. 2011;50(4):299-307.
6. Needle RH, Trotter IRT, Goosby E, Bates C, Von Zinkernagel D. Methodologically sound rapid assessment and response: Providing timely data for policy development on drug use interventions and HIV prevention. International Journal of Drug Policy. 2000;11(1-2):19-23.
7. Needle RH, Trotter RT, 2nd, Singer M, Bates C, Page JB, Metzger D, et al. Rapid assessment of the HIV/AIDS crisis in racial and ethnic minority communities: an approach for timely community interventions. American Journal of Public Health. 2003;93(6):970-9.
8. Holdsworth LM, Safaeinili N, Winget M, Lorenz KA, Lough M, Asch S, et al. Adapting rapid assessment procedures for implementation research using a team-based approach to analysis: a case example of patient quality and safety interventions in the ICU. Implement Sci. 2020;15(1):12.
9. Peiro R. Rapid appraisal methodology for 'health for all' policy formulation analysis. 2003.
10. Peiro R, Alvarez-Dardet C, Plasencia A, Borrell C, Colomer C, Moya C, et al. Rapid appraisal methodology for 'health for all' policy formulation analysis. Health Policy. 2002;62(3):309-28.
11. Fitch C, Rhodes T, Stimson GV. Origins of an epidemic: The methodological and political emergence of rapid assessment. International Journal of Drug Policy. 2000;11(1-2):63-82.
12. Fitch C, Stimson GV, Rhodes T, Poznyak V. Rapid assessment: an international review of diffusion, practice and outcomes in the substance use field. Soc Sci Med. 2004;59(9):1819-30.
13. Stimson GV, Fitch C, DesJarlais D, Poznyak V, Perlis T, Oppenheimer E, et al. Rapid assessment and response studies of injection drug use: knowledge gain, capacity building, and intervention development in a multisite study. American Journal of Public Health. 2006;96(2):288-95.
14. Rhodes T, Stimson GV, Fitch C, Ball A, Renton A. Rapid assessment, injecting drug use, and public health. The Lancet. 1999;354(9172):65-8.
15. Campbell J. A critical appraisal of participatory methods in development research. International Journal of Social Research Methodology. 2002;5(1):19-29.
16. Dale J, Shipman C, Lacock L, Davies M. Creating a shared vision of out of hours care: using rapid appraisal methods to create an interagency, community oriented, approach to service development. Bmj. 1996;312(7040):1206-10.
17. Murray SA. Listening to local voices : adapting rapid appraisal to assess health and social needs in general practice. 1994.
18. Murray SA. Experiences with "rapid appraisal" in primary care: involving the public in assessing health needs, orientating staff, and educating medical students. Bmj. 1999;318(7181):440-4.
19. Harvey HD, Fleming P. A rapid appraisal method for the selection and pre-testing of environmental health leaflets. Journal of The Royal Society for the Promotion of Health. 2000;120(2):112-6.

For peer review only

20. Harvey HD, Fleming P, Patterson M. A rapid appraisal method for reviewing the effectiveness of workplace smoking policies in large and medium sized organisations. Journal of The Royal Society for the Promotion of Health. 2001;121(1):50-5.
21. Green J, Perkins C, Steinbach R, Edwards P. Reduced street lighting at night and health: A rapid appraisal of public views in England and Wales. Health Place. 2015;34:171-80.
22. Beebe J, Given L. Rapid assessment process. The sage encyclopedia of qualitative research methods

Thousand Oaks, California: SAGE Publications; 2012.
23. Hamilton S. Felt needs assessment of mental health (rapid service appraisal): Barking and Havering. (Public health research report, ISSN 09689818 ; no 11). 1993:31.
24. Rowa-Dewar N, Ager W, Ryan K, Hargan I, Hubbard G, Kearney N. Using a rapid appraisal approach in a nationwide, multisite public involvement study in Scotland. Qualitative Health Research. 2008;18(6):863-9.
25. Murphy A, Chikovani I, Uchaneishvili M, Makhashvili N, Roberts B. Barriers to mental health care utilization among internally displaced persons in the republic of Georgia: a rapid appraisal study. BMC health services research. 2018;18(1):306.
26. Foreman-Mackey A, Bayoumi AM, Miskovic M, Kolla G, Strike C. 'It's our safe sanctuary': Experiences of using an unsanctioned overdose prevention site in Toronto, Ontario. International Journal of Drug Policy. 2019;73:135-40.
27. Beebe J. Rapid assessment and response: Sound methodology for producing timely responses. International Journal of Drug Policy. 2000;11(1-2):29-31.
28. Lazenbatt A, Lynch U, O'Neill E. Revealing the hidden 'troubles' in Northern Ireland: The role of participatory rapid appraisal. Health Education Research. 2001;16(5):567-78.
29. Brown CS, Lloyd S, Murray SA. Using consecutive Rapid Participatory Appraisal studies to assess, facilitate and evaluate health and social change in community settings. BMC Public Health. 2006;6 (no pagination)(68).
30. Annett H. Guidelines for rapid participatory appraisals to assess community health needs: a focus on health improvements for low-income urban and rural areas. Geneva: Division of Strengthening of Health Services World Health Organization available from HMSO; 1995. iv,60 p.
31. Lawlor D. Rapid participatory appraisal of young people's sexual health needs: an evaluation of meta-planning. 1999.
32. Desai A, Donetto S. Evolving ethnographic sensibilities: Using actor-network theory in health services research. BMJ Open. 2019;9 (Supplement 1):A5.
33. Vindrola-Padros C, Vindrola-Padros B. Quick and dirty? A systematic review of the use of rapid ethnographies in healthcare organisation and delivery. BMJ Qual Saf. 2018;27(4):321-30.
34. Hanckel B, Ruta D, Scott G, Peacock JL, Green J. The Daily Mile as a public health intervention: a rapid ethnographic assessment of uptake and implementation in South London, UK. BMC Public Health. 2019;19(1):1167.
35. Boyd J, Collins AB, Mayer S, Maher L, Kerr T, McNeil R. Gendered violence and overdose prevention sites: a rapid ethnographic study during an overdose epidemic in Vancouver, Canada. Addiction. 2018;113(12):2261-70.
36. Goepp JG, Meykler S, Mooney NE, Lyon C, Raso R, Julliard K. Provider insights about palliative care barriers and facilitators: results of a rapid ethnographic assessment. Am J Hosp Palliat Care. 2008;25(4):309-14.
37. Green TC, Zaller N, Palacios WR, Bowman SE, Ray M, Heimer R, et al. Law enforcement attitudes toward overdose prevention and response. Drug Alcohol Depend. 2013;133(2):677-84.
38. Ackerman S, Sarkar U, Tieu L, Hamdley M, Schillinger D, Jahn K, et al. Meaningful use in the safety net: A rapid ethnography of patient portal implementation at five community health centers in California. Journal of the American Medical Informatics Association. 2017;24(5):903-12.
39. Quintero Romero S, Bernal R, Barbiero C, Passamonte R, Cattaneo A. A rapid ethnographic study of breastfeeding in the North and South of Italy. Int Breastfeed J. 2006;1:14.

For peer review only

40. Kroeger K, Willilams S, Alexander-Pender C, Ford J, Vasani V. "Just one thing on the plate" - Views of community members and providers on sexual health services in the fort mcpherson area of Southwest Atlanta: Findings from a rapid ethnographic assessment. Sexually Transmitted Infections Conference: STI and AIDS World Congress. 2013;89(SUPPL. 1).
41. Dupont HB, Kaplan CD, Braam RV, Verbraeck HT, de Vries NK. The application of the rapid assessment and response methodology for cannabis prevention research among youth in the Netherlands. International Journal of Drug Policy. 2015;26(8):731-8.
42. Ong BN. Rapid appraisal and health policy. 1996.
43. Ong BN, Humphris G, Annett H, Rifkin S. Rapid appraisal in an urban setting, an example from the developed world. Soc Sci Med. 1991;32(8):909-15.
44. Organization WH, UNAIDS. SEX-RAR Guide. https://apps.who.int/iris/bitstream/handle/10665/42681/9241545585.pdf?sequence=1&isAllowed=y2002.
45. Trotter IRT, Needle R, Goosby E, Bates C, Singer M. A Methodological Model for Rapid Assessment, Response, and Evaluation: The RARE Program in Public Health. Field Methods. 2001;13(2):137-59.
46. Brown DR, Hernandez A, Saint-Jean G, Evans S, Tafari I, Brewster LG, et al. A participatory action research pilot study of urban health disparities using rapid assessment response and evaluation. American Journal of Public Health. 2008;98(1):28-38.
47. Burks DJ, Robbins R, Durtschi JP. American Indian gay, bisexual and two-spirit men: a rapid assessment of HIV/AIDS risk factors, barriers to prevention and culturally-sensitive intervention. Culture, health & sexuality. 2011;13(3):283-98.
48. Valderrama JC, Tortajada S, Alapont L, Vidal A, Perez Ma J, Castellano M, et al. Rapid assessment of drug abuse prevention needs for youth in small Spanish municipalities: Coping with resource limitations. Journal of Drug Issues. 2006;36(1):29-52.
49. Sperber NR, Bruening RA, Choate A, Mahanna E, Wang V, Powell BJ, et al. Implementing a Mandated Program Across a Regional Health Care System: A Rapid Qualitative Assessment to Evaluate Early Implementation Strategies. Quality management in health care. 2019;28(3):147-54.
50. Keith RE, Crosson JC, O'Malley AS, Cromp D, Taylor EF. Using the Consolidated Framework for Implementation Research (CFIR) to produce actionable findings: a rapid-cycle evaluation approach to improving implementation. Implement Sci. 2017;12(1):15.
51. Shrank WH. The Center For Medicare And Medicaid Innovation's blueprint for rapid-cycle evaluation of new care and payment models. Health Affairs (Millwood). 2013;32(4):807-12.
52. Fergus CJY. Assessing and improving out-of-hours palliative care in a deprived community: a rapid appraisal study. 2010.
53. Kercsmar CM, Beck AF, Sauers-Ford H, Simmons J, Wiener B, Crosby L, et al. Association of an asthma improvement collaborative with health care utilization in medicaid-insured pediatric patients in an urban community. JAMA Pediatrics. 2017;171(11):1072-80.
54. Farrer K, Donaldson E, Blackett B, Lloyd H, Forde C, Melia D, et al. Nutritional screening of elderly patients: a health improvement approach to practice. J Hum Nutr Diet. 2014;27(2):184-91.
55. Schneeweiss S, Shrank WH, Ruhl M, Maclure M. Decision-making aligned with rapid-cycle evaluation in health care. International Journal of Technology Assessment in Health Care. 2015;31(4):214-22.
56. Palinkas LA, Mendon SJ, Hamilton AB. Innovations in Mixed Methods Evaluations. Annual Review of Public Health. 2019;40:423-42.
57. Palinkas LA, Zatzick D. Rapid Assessment Procedure Informed Clinical Ethnography (RAPICE) in Pragmatic Clinical Trials of Mental Health Services Implementation: Methods and Applied Case Study. Adm Policy Ment Health. 2019;46(2):255-70.
58. Guise A, Witzel TC, Mandal S, Sabin C, Rhodes T, Nardone A, et al. A qualitative assessment of the acceptability of hepatitis C remote self-testing and self-sampling amongst people who use drugs in London, UK. BMC Infectious Diseases. 2018;18 (1) (no pagination)(281).

For peer review only

59. Phillips CB, Dwan K, Hepworth J, Pearce C, Hall S. Using qualitative mixed methods to study small health care organizations while maximising trustworthiness and authenticity. BMC Health Services Research. 2014;14:559.
60. Williams SP, Kinsey J, Carry MG, Terry L, Wells J, Kroeger K. Get In, Get Tested, Get Care: STD Services in Urban Urgent Care Centers. Sexually Transmitted Diseases. 2019;46(10):648-53.
61. Dunet DO, Sparling PB, Hersey J, Williams-Piehota P, Hill MD, Hanssen C, et al. A new evaluation tool to obtain practice-based evidence of worksite health promotion programs. Preventing chronic disease. 2008;5(4):A118.
62. Gray TA, Dumville JC, Christie J, Cullum NA. Rapid research and implementation priority setting for wound care uncertainties. PLoS ONE. 2017;12(12):e0188958.
63. Aspray TJ, Nesbit K, Cassidy TP, Hawthorne G. Rapid assessment methods used for health-equity audit: Diabetes mellitus among frail British care-home residents. Public Health. 2006;120(11):1042-51.
64. Banga S, Chabanon AL, Eymin C, Caroe T, Butler K, Moureau A. Near real-time safety surveillance of three seasonal influenza vaccines in Europe during influenza season 2017/18. Pharmacoepidemiology and Drug Safety. 2018;27 (Supplement 2):396.
65. Begg NT, Gill ON, White JM. COVER (Cover Of Vaccination Evaluated Rapidly): Description of the England and Wales scheme. Public Health. 1989;103(2):81-9.
66. Brook MG, Rusere L, Coppin-Browne L, McDonagh S, McSorley J. A prospective study of the effectiveness of electronic patient records in rapid-cycle assessment of treatment and partner notification outcomes for patients with genital chlamydia and gonorrhoea infection. Sexually Transmitted Infections. 2011;87(2):152-5.
67. Gray A, Baker K. Assessment for change. Acute Medicine. 2017;16 (3):127-8.
68. Joseph C, Goddard N. Influenza vaccine uptake in the elderly: Results from a rapid assessment of the effectiveness of new government policy in England for the winters 2000/2001 and 2001/2002. Vaccine. 2003;21(11-12):1137-48.
69. Lester C. Priority setting with Health Inequality Impact Assessment. 2002.
70. Newlands RS, Power A, Young L, Watson M. Quality improvement of community pharmacy services: a prioritisation exercise. International Journal of Pharmacy Practice. 2018;26(1):39-48.
71. Alguacil-Ramos AM, Portero-Alonso A, Pastor-Villalba E, Muelas-Tirado J, Diez-Domingo J, Sanchis-Ferrer A, et al. Rapid assessment of enhanced safety surveillance for influenza vaccine. Public Health. 2019;168:137-41.
72. Baum RA, Rutter CM, Sunshine JH, Blebea JS, Blebea J, Carpenter JP, et al. Multicenter trial to evaluate vascular magnetic resonance angiography of the lower extremity. American College of Radiology Rapid Technology Assessment Group. Jama. 1995;274(11):875-80.
73. Brown-Johnson C, Lo N, Giannitrapani K, Lowery J, Foglia MB, Walling A, et al. A Rapid Qualitative Synthesis of Insights from the Veterans Administration Life-Sustaining Treatment Decisions Initiative (LSTDI) National Implementation (QI703). Journal of Pain and Symptom Management. 2019;57 (2):457.
74. Cakir B, Uner S, Temel F, Akin L. Lot quality survey: An appealing method for rapid evaluation of vaccine coverage in developing countries - Experience in Turkey. BMC Public Health. 2008;8 (no pagination)(240).
75. Centers for Disease C, Prevention. Rapid assessment of influenza vaccination coverage among HMO members--northern California influenza seasons, 2001-02 through 2004-05. MMWR Morb Mortal Wkly Rep. 2005;54(27):676-8.
76. Currow DC, Vella-Brincat J, Fazekas B, Clark K, Doogue M, Rowett D. Pharmacovigilance in hospice/palliative care: Rapid report of net clinical effect of metoclopramide. Journal of Palliative Medicine. 2012;15(10):1071-5.
77. D'Amore M, Masters P, Maroun C. Impact of an automatic therapeutic interchange program on discharge medication selection. Hospital Pharmacy. 2003;38(10):942-6.

For peer review only

78. Delaney AE, Dadlez NM, Marshall AC. Alternative approach to pediatric cardiac quality assessment for low-volume centers. Congenital Heart Disease. 2019;14(4):665-70.
79. Demeulemeester M, Lavis N, Balthazar Y, Lechien P, Heijmans S. Rapid safety assessment of a seasonal intradermal trivalent influenza vaccine. Hum Vaccin Immunother. 2017;13(4):889-94.
80. Dunbar MS, Towe VL, Ayer L, Martineau M. Connections to Care (C2C): The Perspectives of Leaders at Community-Based Organizations That Are Integrating Mental Health Supports. Rand health q. 2018;7(4):7.
81. Gagne JJ, Wang S, Schneeweiss S. A multivariable-adjusted rapid assessment of the association between zoledronic acid and myocardial infarction. Pharmacoepidemiology and Drug Safety. 2013;1):378.
82. Hamarman A, Mason P, Kroeger K, Alexander-Pender C. The impact of budget cuts among local STD programs in New Jersey: Results of a rapid ethnographic assessment. Sexually Transmitted Diseases. 2014;1):S93.
83. Hensel JM, Shaw J, Jeffs L, Ivers NM, Desveaux L, Cohen A, et al. A pragmatic randomized control trial and realist evaluation on the implementation and effectiveness of an internet application to support self-management among individuals seeking specialized mental health care: A study protocol. BMC Psychiatry. 2016;16 (1) (no pagination)(350).
84. Livorsi D, Knobloch MJ, Blue LA, Swafford K, Maze L, Riggins K, et al. A rapid assessment of barriers and facilitators to safety culture in an intensive care unit. Int Nurs Rev. 2016;63(3):372-6.
85. Loosier PS, Carter MW, Hsu KK, Doshi S, Peterson Maddox BL, Kroeger K, et al. Provision of STD Services in Community Settings After the Loss and Return of State Funding to Support Service Provision: Observations From Select Providers in Massachusetts, 2010 and 2013. Journal of public health management and practice : JPHMP. 2020;26(1):E18-E27.
86. MacCarthy S, Barreras JL, Mendoza-Graf A, Galvan F, Linnemayr S. Strategies for Improving Mobile Technology-Based HIV Prevention Interventions With Latino Men Who Have Sex With Men and Latina Transgender Women. AIDS Educ Prev. 2019;31(5):407-20.
87. Seay J, Ranck A, Weiss R, Salgado C, Fein L, Kobetz E. Understanding Transgender Men's Experiences with and Preferences for Cervical Cancer Screening: A Rapid Assessment Survey. LGBT health. 2017;4(4):304-9.
88. Seay JS, Carrasquillo O, Campos NG, McCann S, Amofah A, Pierre L, et al. Cancer Screening Utilization Among Immigrant Women in Miami, Florida. Prog. 2015;9 Suppl:11-20.
89. Solomon P, Tennille J, Lipsitt D, Plumb E, Metzger D, Blank MB. Rapid assessment of existing HIV prevention programming in a Community Mental Health Center. Journal of Prevention and Intervention in the Community. 2006;33(1-2):137-51.
90. Wichmann O, Stocker P, Poggensee G, Altmann D, Walter D, Hellenbrand W, et al. Pandemic influenza A(H1N1) 2009 breakthrough infections and estimates of vaccine effectiveness in Germany 2009-2010. Eurosurveillance. 2010;15(18):1-4.
91. Porter A, Black S, Dale J, Harris-Mayes R, Lawrenson R, Lyons R, et al. Electronic records in ambulances-an observational study (ERA). Emergency Medicine Journal. 2019;36 (10):E14.
92. Wiss DA, Schellenberger M, Prelip ML. Rapid Assessment of Nutrition Services in Los Angeles Substance Use Disorder Treatment Centers. Journal of community health. 2019;44(1):88-94.
93. Ash JS, Sittig DF, McMullen CK, Guappone K, Dykstra R, Carpenter J. A rapid assessment process for clinical informatics interventions. Amia 2008;Annual Symposium proceedings / AMIA Symposium. AMIA Symposium.:26-30.
94. Belford M, Robertson T, Jepson R. Using evaluability assessment to assess local community development health programmes: a Scottish case-study. BMC medical research methodology. 2017;17(1):70.
95. Boucaud-Maitre D, Altman JJ. Do the EMA accelerated assessment procedure and the FDA priority review ensure a therapeutic added value? 2006-2015: a cohort study. European Journal of Clinical Pharmacology. 2016;72(10):1275-81.

For peer review only

96. Haigh F, Harris E, Chok HN, Baum F, Harris-Roxas B, Kemp L, et al. Characteristics of health impact assessments reported in Australia and New Zealand 2005-2009. Australian and New Zealand journal of public health. 2013;37(6):534-46.
97. Hailey D. The use and impact of rapid health technology assessments. 2001.

For peer review only

Preferred Reporting Items for Systematic reviews and Meta-Analyses extension for Scoping Reviews (PRISMA-ScR) Checklist

SECTION	ITEM	PRISMA-ScR CHECKLIST ITEM	REPORTED ON PAGE #
**TITLE**			
Title	1	Identify the report as a scoping review.	1
**ABSTRACT**			
Structured summary	2	Provide a structured summary that includes (as applicable): background, objectives, eligibility criteria, sources of evidence, charting methods, results, and conclusions that relate to the review questions and objectives.	2
**INTRODUCTION**			
Rationale	3	Describe the rationale for the review in the context of what is already known. Explain why the review questions/objectives lend themselves to a scoping review approach.	3
Objectives	4	Provide an explicit statement of the questions and objectives being addressed with reference to their key elements (e.g., population or participants, concepts, and context) or other relevant key elements used to conceptualize the review questions and/or objectives.	4
**METHODS**			
Protocol and registration	5	Indicate whether a review protocol exists; state if and where it can be accessed (e.g., a Web address); and if available, provide registration information, including the registration number.	4
Eligibility criteria	6	Specify characteristics of the sources of evidence used as eligibility criteria (e.g., years considered, language, and publication status), and provide a rationale.	5
Information sources*	7	Describe all information sources in the search (e.g., databases with dates of coverage and contact with authors to identify additional sources), as well as the date the most recent search was executed.	5
Search	8	Present the full electronic search strategy for at least 1 database, including any limits used, such that it could be repeated.	Supplementary material
Selection of sources of evidence†	9	State the process for selecting sources of evidence (i.e., screening and eligibility) included in the scoping review.	5,6
Data charting process‡	10	Describe the methods of charting data from the included sources of evidence (e.g., calibrated forms or forms that have been tested by the team before their use, and whether data charting was done independently or in duplicate) and any processes for obtaining and confirming data from investigators.	7
Data items	11	List and define all variables for which data were sought and any assumptions and simplifications made.	7
Critical appraisal of individual	12	If done, provide a rationale for conducting a critical appraisal of included sources of evidence; describe	N/A

For peer review only - http://bmjopen.bmj.com/site/about/guidelines.xhtml

SECTION	ITEM	PRISMA-ScR CHECKLIST ITEM	REPORTED ON PAGE #
sources of evidence§		the methods used and how this information was used in any data synthesis (if appropriate).	
Synthesis of results	13	Describe the methods of handling and summarizing the data that were charted.	8
**RESULTS**			
Selection of sources of evidence	14	Give numbers of sources of evidence screened, assessed for eligibility, and included in the review, with reasons for exclusions at each stage, ideally using a flow diagram.	7,8 (Figure 1)
Characteristics of sources of evidence	15	For each source of evidence, present characteristics for which data were charted and provide the citations.	Click here to enter text.
Critical appraisal within sources of evidence	16	If done, present data on critical appraisal of included sources of evidence (see item 12).	N/A
Results of individual sources of evidence	17	For each included source of evidence, present the relevant data that were charted that relate to the review questions and objectives.	P9, Table 2; supplementary information
Synthesis of results	18	Summarize and/or present the charting results as they relate to the review questions and objectives.	Click here to enter text.
**DISCUSSION**			
Summary of evidence	19	Summarize the main results (including an overview of concepts, themes, and types of evidence available), link to the review questions and objectives, and consider the relevance to key groups.	10-19
Limitations	20	Discuss the limitations of the scoping review process.	20,21
Conclusions	21	Provide a general interpretation of the results with respect to the review questions and objectives, as well as potential implications and/or next steps.	22,23
**FUNDING**			
Funding	22	Describe sources of funding for the included sources of evidence, as well as sources of funding for the scoping review. Describe the role of the funders of the scoping review.	24

JBI = Joanna Briggs Institute; PRISMA-ScR = Preferred Reporting Items for Systematic reviews and Meta-Analyses extension for Scoping Reviews.
* Where *sources of evidence* (see second footnote) are compiled from, such as bibliographic databases, social media platforms, and Web sites.
† A more inclusive/heterogeneous term used to account for the different types of evidence or data sources (e.g., quantitative and/or qualitative research, expert opinion, and policy documents) that may be eligible in a scoping review as opposed to only studies. This is not to be confused with *information sources* (see first footnote).
‡ The frameworks by Arksey and O'Malley (6) and Levac and colleagues (7) and the JBI guidance (4, 5) refer to the process of data extraction in a scoping review as data charting.
§ The process of systematically examining research evidence to assess its validity, results, and relevance before using it to inform a decision. This term is used for items 12 and 19 instead of "risk of bias" (which is more applicable to systematic reviews of interventions) to include and acknowledge the various sources of evidence that may be used in a scoping review (e.g., quantitative and/or qualitative research, expert opinion, and policy document).

From: Tricco AC, Lillie E, Zarin W, O'Brien KK, Colquhoun H, Levac D, et al. PRISMA Extension for Scoping Reviews (PRISMAScR): Checklist and Explanation. Ann Intern Med. 2018;169:467–473. doi: 10.7326/M18-0850.

For peer review only - http://bmjopen.bmj.com/site/about/guidelines.xhtml

Approaches to enabling rapid evaluation of innovations in health and social care: a scoping review of evidence from high income countries

Journal:	*BMJ Open*
Manuscript ID	bmjopen-2022-064345.R1
Article Type:	Original research
Date Submitted by the Author:	23-Nov-2022

Complete List of Authors:	Norman, Gill; The University of Manchester Faculty of Biology Medicine and Health<br>Mason, Thomas; University of Manchester; Lancaster University, Division of Health Research<br>Dumville, Jo C.; The University of Manchester<br>Bower, Peter; The University of Manchester Faculty of Biology Medicine and Health, NPCRDC<br>Wilson, Paul; University of Manchester, Alliance Manchester Business School<br>Cullum, Nicky; The University of Manchester Faculty of Biology Medicine and Health, School of Health Sciences; Manchester University NHS Foundation Trust, MAHSC

**Primary Subject Heading**:	Research methods
Secondary Subject Heading:	Health services research
Keywords:	Organisation of health services < HEALTH SERVICES ADMINISTRATION & MANAGEMENT, Quality in health care < HEALTH SERVICES ADMINISTRATION & MANAGEMENT, STATISTICS & RESEARCH METHODS

SCHOLARONE™
Manuscripts

I, the Submitting Author has the right to grant and does grant on behalf of all authors of the Work (as defined in the below author licence), an exclusive licence and/or a non-exclusive licence for contributions from authors who are: i) UK Crown employees; ii) where BMJ has agreed a CC-BY licence shall apply, and/or iii) in accordance with the terms applicable for US Federal Government officers or employees acting as part of their official duties; on a worldwide, perpetual, irrevocable, royalty-free basis to BMJ Publishing Group Ltd ("BMJ") its licensees and where the relevant Journal is co-owned by BMJ to the co-owners of the Journal, to publish the Work in this journal and any other BMJ products and to exploit all rights, as set out in our licence.

The Submitting Author accepts and understands that any supply made under these terms is made by BMJ to the Submitting Author unless you are acting as an employee on behalf of your employer or a postgraduate student of an affiliated institution which is paying any applicable article publishing charge ("APC") for Open Access articles. Where the Submitting Author wishes to make the Work available on an Open Access basis (and intends to pay the relevant APC), the terms of reuse of such Open Access shall be governed by a Creative Commons licence – details of these licences and which Creative Commons licence will apply to this Work are set out in our licence referred to above.

Other than as permitted in any relevant BMJ Author's Self Archiving Policies, I confirm this Work has not been accepted for publication elsewhere, is not being considered for publication elsewhere and does not duplicate material already published. I confirm all authors consent to publication of this Work and authorise the granting of this licence.

For peer review only

Approaches to enabling rapid evaluation of innovations in health and social care: a scoping review of evidence from high income countries

Gill Norman[1,4], Thomas Mason[2,3], Jo Dumville[1,4], Peter Bower[3,4], Paul Wilson[3,4], Nicky Cullum[1,4]

Affiliations

1. Division of Nursing, Midwifery & Social Work; School of Health Sciences; Faculty of Biology, Medicine & Health, University of Manchester, Manchester, UK.

2. Division of Health Research, Lancaster University, Lancaster, United Kingdom, LA1 4YW

3. Centre for Primary Care and Health Services Research, School of Health Sciences; Faculty of Biology, Medicine & Health, University of Manchester; Manchester, UK.

4. Manchester Academic Health Science Centre, Research and Innovation Division, Manchester University Foundation NHS Trust, Manchester, UK.

Address for correspondence

Gill Norman
Division of Nursing, Midwifery & Social Work;
School of Health Sciences;
Faculty of Biology, Medicine & Health,
University of Manchester
Oxford Road
Manchester
M13 9PL
E: gill.norman@manchester.ac.uk

References: 245
Abstract word count: 296
Main text word count: 6406

For peer review only

ABSTRACT

Objective: The COVID-19 pandemic increased demand for rapid evaluation of innovation in health and social care. Assessment of rapid methodologies is lacking although challenges in ensuring rigour and effective use of resources are known. We mapped reports of rapid evaluations of health and social care innovations, categorised different approaches to rapid evaluation, explored comparative, and identified knowledge gaps.

Design: Systematic scoping review

Data sources: MEDLINE, EMBASE and Health Management Information Consortium (HMIC) databases were searched through13 September 2022.

Eligibility criteria for selecting studies: We included publications reporting primary research or methods for rapid evaluation of interventions or services in health and social care in high income countries.

Data extraction and synthesis: Two reviewers developed and piloted a data extraction form. One reviewer extracted data, a second reviewer checked 10% of studies; disagreements and uncertainty were resolved through consensus. We used narrative synthesis to map different approaches to conducting rapid evaluation.

Results: We identified 16759 records and included 162 which met inclusion criteria.

We identified four main approaches for rapid evaluation:

(1) Using methodology designed specifically for rapid evaluation;
(2) Increasing rapidity by doing less or using less time-intensive methodology;
(3) Using alternative technologies and/or data to increase speed of existing evaluation method;
(4) Adapting part of non-rapid evaluation

The COVID-19 pandemic resulted in an increase in publications and some limited changes in identified methods. We found little research comparing rapid and non-rapid evaluation.

Conclusions: We found a lack of clarity about what 'rapid evaluation' means but identified some useful preliminary categories. There is a need for clarity and consistency about what constitutes rapid evaluation; consistent terminology in reporting evaluations as rapid; development of specific methodologies for making evaluation more rapid; and assessment of advantages and disadvantages of rapid methodology in terms of rigour, cost and impact.

For peer review only - http://bmjopen.bmj.com/site/about/guidelines.xhtml

Strengths and limitations of this study

- Strengths include a prespecified, published review protocol which anticipated the need for flexibility in the structure and focus of a wide-ranging scoping review. Our paper adheres internationally adopted recommendations for reporting scoping reviews (PRISMA-ScR).
- A further strength is our search strategy which was developed and implemented with an information specialist and covered multiple databases.
- One limitation of this study is our dependence on whether and how study authors described their studies as “rapid” and the lack of clarity in the reporting of the included studies.
- A further limitation is that we did not screen or chart every study in duplicate, however analysis of those that we did assess in duplicate suggests this is unlikely to have impacted substantially on the review content.

INTRODUCTION

Background

The need to support innovation in health and social care sits alongside the need to ensure that innovations are appropriately evaluated to ensure safe, effective and cost-effective care for patients and service users. In the absence of adequate evaluation new technologies or practices may be adopted which do not have proven benefits and which may even result in harm. However, evaluation needs to be conducted quickly and efficiently if it is to provide timely information for decisionmakers. There is increasing demand for timely, rigorous evaluation of innovation in health and social care. Demand has been amplified during the COVID-19 pandemic, where the ability of research infrastructure to respond rapidly has been paramount.

The term ‘rapid evaluation’ is widely used to describe approaches that aim to adopt pragmatic methods for timely assessment of innovations, but there is uncertainty about what this means in practice. Rapid evaluation can be conceptualised to include rapid conception and inception of evaluation in response to an identified need, or rapid completion of part or all of the evaluation process, but linking these ideas to practice still requires investigation.

Whilst the need for rapidity in evaluation is widely recognised, there has been limited assessment of how rapidity can be actually achieved (1-3); this is particularly the case for primary evaluations as opposed to rapid reviews (4-8). There is a growing body of research

For peer review only - http://bmjopen.bmj.com/site/about/guidelines.xhtml

on the development of formal methods specifically designed for rapid evaluation (9-11), but this forms only part of a wider literature reporting "rapid" studies.

Scoping reviews of evaluation approaches can map available approaches, identify gaps where further development is needed and provide insights into the strengths and weaknesses of existing methods (12, 13). Understanding the different types of approaches and the range of methods adopted by researchers to achieve rapidity of evaluation in practice may inform the development of new approaches to evaluation of innovations. It also has the potential to provide additional insights into the trade-offs and limitations which may result from rapidity in terms of rigour or transferability of findings (14). Finally it may identify research questions in which there has been limited use of rapid evaluation approaches and where further development might be prioritised.

We therefore undertook a scoping review to map reports of rapid evaluations of health and social care innovations; map the types of question asked in rapid evaluations and to identify where further research is needed to support more rapid evaluation. We focused the review on studies from high-income countries to increase relevance to the National Health Services in the UK.

Research Questions

In this scoping review we explored the following research questions:

- What methodologies and methods relevant to a UK context have been used to undertake rapid evaluations of health and social care innovations? How are these approaches expected to deliver rapidity?

- What is the comparative evidence for rapid evaluation methodologies? Have these methodologies been compared to non-rapid standard (however defined) methods or other rapid methodologies?

METHODS

Approaches to scoping for rapid evaluations

In this scoping review, we aimed to: identify and summarise evaluations addressing health-related questions in ways which had been described as 'rapid'; document the ways in which study authors considered that evaluation was made 'rapid'; and summarise evidence that compares the risks and benefits of rapid evaluation and alternative approaches. We accepted authors' definitions of what constituted 'rapid evaluation'. We considered evaluations of the safety and effectiveness of innovations as well as a wider set of outcomes, including acceptability and user experience. In this scoping review we did not seek to assess the quality of studies.

For peer review only - http://bmjopen.bmj.com/site/about/guidelines.xhtml

We adopt the definition of innovation as: *any novel technology, device, procedure, set of behaviours, routine, or way(s) of working that is directed at improving health outcomes, administrative efficiency, cost-effectiveness or users' experience - and is implemented by planned and coordinated actions* (15). Thus, innovation can include alterations in service delivery, organisation and financing, as well as clinical and care practices.

We considered evaluation as a complex and multifaceted undertaking which lacks a single definition. Using a broad definition that encompasses research, typically evaluation investigates whether an overall net benefit is gained from innovation over alternative activities. Phase III randomised controlled trials (RCTs) and systematic reviews of innovations remain the 'gold-standard' of comparative effectiveness methods; although quasi-randomised approaches are often more feasible. For other uncertainties around innovations (e.g. acceptability, feasibility) there are well established quantitative, qualitative and mixed methods (and corresponding approaches to evidence synthesis).

We adopted an iterative approach to review methods which we planned for in our protocol, which was completed before the screening stage of the review and published on OSF (16). In particular we have narrowed the scope of the review and focused on addressing a subset of the original questions posed. We have done this in order to engage fully with the questions we considered most central to our objectives (see above). We have followed the reporting approach recommended in the extension of PRISMA for scoping reviews (PRISMA-ScR) where possible given this planned flexibility (17).

Inclusion eligibility

Our inclusion criteria (Table 1) were developed using a PICOS approach and used to screen the results of the database search. We developed the inclusion criteria iteratively to ensure that the most relevant research was captured, anticipating this flexibility in the protocol.

We only included publications in English. We planned to document relevant non-English publications but not to extract data or include them due to the available resources. However, the great majority of non-English publications identified were ineligible (based on assessment of an English language abstract).

Search

We searched PubMed/MEDLINE, EMBASE and Health Management Information Consortium (HMIC) databases. This main search was updated on 13 September 2022, having initially been conducted in February 2020). We also looked at websites of key organisations involved in rapid evaluations (e.g. RSET, BRACE, Nuffield Trust and Health Foundation, THIS Institute) and checked references of identified studies. Finally, we searched Google Scholar and checked the first 1000 records against the main database searches. The database search strategy (Appendix 1, Supplementary information) was designed and implemented by an information specialist. We selected terms to balance sensitivity and specificity. There were

For peer review only - http://bmjopen.bmj.com/site/about/guidelines.xhtml

two main elements to the initial search: a search for rapid evaluations undertaken from database inception, and a search from 2014 onwards using the search strategy from a previous, high-quality scoping review of rapid reviews which had searched up to 2014 (5).

We subsequently narrowed the scope of our search to exclude rapid evidence synthesis (i.e. rapid reviews (Table 1)) as it became clear that the volume of recent rapid reviews and related methodological papers would require a separate piece of work. Given that considerable work on rapid review methodology is being undertaken (18-21), we therefore took the decision to focus on primary research. Our update search in September 2022 did not include this facet. While we excluded papers on rapid synthesis, we nevertheless included syntheses of rapid primary studies (Table 1).

Table 1: Eligibility Criteria

**Study design:** Publications which evaluate, compare, document the use of, provide guidance on, or otherwise describe one or more rapid evaluation methods for assessing innovations according to the study authors' definition (of rapid evaluation). We also included previous systematic or scoping reviews of rapid evaluations but noted overlap with included primary studies. We did not include rapid reviews or papers relating to methods for rapid reviews. Where studies used a rapid review as the first stage of primary evaluation, we included them only if the primary evaluation element was also described as rapid or using rapid methods. Evaluations could be primarily quantitative or qualitative in focus (or use mixed methods). We also excluded pilot studies which were not explicitly described as rapid evaluations.
**Innovations (interventions):** Publications on rapid evaluation of innovations or their implementation in the field of health and of social care as it relates to health. Innovation is broadly defined as encompassing intentional change (15). We subsequently broadened this to encompass interventions or services not described as innovations. We excluded publications reporting methods which related only tangentially to interventions or services such as Rapid Assessments of Needs and Rapid Epidemiological Assessments.
**Populations:** We included any study relating to rapid evaluation methods and did not further limit eligibility based on the study population beyond the requirement that the study be undertaken in a health or related care setting in a high-income country using World Bank definitions (22). We also excluded studies which looked at evaluation in a regulatory context because these represented accelerated evidence review processes and were therefore considered to represent review methodology rather than primary evaluation.
**Outcomes:** We accepted any health-related outcome measure; this includes any clinical measure relevant to the intervention including patient-reported outcomes or experience; measures of care process or service use; and measurements of costs or activity as well as health economic measures such as cost-effectiveness. This was used to ensure the relevance of the study to health and related social care.

For peer review only - http://bmjopen.bmj.com/site/about/guidelines.xhtml

Screening

One researcher screened initial search results by title and abstract in Endnote and selected potentially relevant records for full text evaluation (23). A 5% sample was exported to Microsoft Excel (Version 365) and screened by a second researcher for consistency. Records obtained at full text were screened by one researcher, with a second researcher assessing 10% to check consistency, and subsequently available for discussion in cases of uncertainty. We did not undertake duplicate screening for all records because of the high volume of retrieved records and because the sample which were duplicate screened showed a very high level of consistency (at abstract stage we identified one additional record requiring full-text assessment for every 200 records dual screened; only a single included study was identified which would have been excluded in error). We adopted a conservative approach to full text exclusion decisions and resolved uncertainty through discussion.

Data extraction

We extracted sufficient information to enable us to classify studies on their key characteristics and to thereby 'chart' the available literature (17, 24). Following the recommendations of Levac (25) we piloted a bespoke data form using Microsoft Excel to enable data extraction, using two researchers, with a small sample of included studies. The following data were extracted by one researcher (with a 10% sample cross-checked by a second researcher, who was also consulted in cases of uncertainty).

- Study ID (First author, year of publication)
- Country (individual); country group (Africa, Asia/middle east, Europe, North America, Central/South America), OECD class (low and middle income (LMIC) or high income)
- Definition(s) of rapid evaluation used by authors including timescale and whether the focus was evaluation/implementation/methods
- Study designs as described by authors (qualitative/quantitative/mixed methods)
- Question(s) the evaluation sought to address
- Collection/analysis methods (e.g. survey, observation, interviews)
- Nature of innovations evaluated (if practical example)
- Impact of rapid methods (for comparative studies)
- Stage, scope, characteristics and purpose of evaluation e.g. local/regional/national; formative/iterative/summative
- Evaluation methods adaptation to context/complexity of intervention including whether stakeholder participation was reported. This was based on the relevant items from the TiDIER scale (26).

We undertook this charting across all studies that met our inclusion criteria. These data were then used to map studies in terms of what made the study 'rapid'. The classification was undertaken by one researcher and discussed by the review team.

For peer review only - http://bmjopen.bmj.com/site/about/guidelines.xhtml

Synthesis and analysis

We developed a narrative synthesis of the evidence relating to each of our two review questions. We structured the synthesis by which question the evidence was addressing and by the methods used to make evaluation rapid. We used data charting to identify studies which shared common features and to group them into categories based on the different approaches to enabling rapidity which were represented. We used discussion and consensus to refine these categories. The synthesis was supported by structured tables to inform the approaches identified and the ways in which these were implemented.

RESULTS

Results of the search and screening process

We identified 16,759 unique records (Figure 1). Preliminary assessment determined 1835 records to be potentially eligible from which we excluded 1337 records of rapid reviews, primary studies not related to rapid evaluation or studies not reported in English. We excluded a further 272 records of studies in LMICs and 64 which looked at regulatory process evaluations (a list of excluded studies is available on request).

We included 162 studies in the review (Table 2); these were methods papers, primary evaluations or evaluations of implementation undertaken in high income countries; one was a systematic review (9) which included eight of the primary studies included here (27-33). Ninety of the included studies were identified from the update search in September 2022. Most studies were undertaken in primary care, community settings, or in specific non-hospital-based services (often described as 'clinics'), reflecting the health and related care focus of the review. Further details of the included studies are reported in Appendix 2 in Supplementary Tables 1-4 (see Supplementary information).

For peer review only

Table 2: Summary table of included studies

**Means of making evaluation rapid** [1] [2]	**Examples of approach**
Methods specifically designed for rapid evaluation: 117 studies with supporting methods papers	Rapid evaluation or assessment/appraisal process/procedure and adaptations (9, 14, 34-89)<br><br>Rapid assessment and response and adaptations (11, 39, 40, 45, 90-98)<br><br>Rapid participatory appraisal and adaptations (50, 51, 99-105)<br><br>Rapid ethnographic assessment/ethnography and adaptations (106-131)<br><br>Methods from quality improvement (28, 29, 31-33, 105, 132-143)
Doing less or using a less time-intensive version of an existing methodology: 28 studies	Shorter research period (144-147)<br><br>Less intensive method (61, 88, 148-159)<br><br>Fewer participants or fewer/smaller study sites (67, 110, 148, 160-167)
Adaptation of part of a standard method: 44studies	Priority setting (168-171)<br><br>Ethics approval, recruitment and participation, barriers (28, 150-155, 159, 172-183)<br><br>Study design (184-191)<br><br>Data collection and analysis (156, 158, 185, 192-203)
Use of alternative technology or data source: 28 studies	Automation or otherwise increased speed of data collection, collation or analysis (61, 148, 150-156, 158, 159, 204-206)<br><br>Acquisition or use of existing data sets (140, 183, 192, 207-217)

[1] Some studies appear in more than one category because multiple means were employed
[2] Some studies in each category are represented by multiple references

For peer review only - http://bmjopen.bmj.com/site/about/guidelines.xhtml

Methodologies and methods used for rapid evaluation

After mapping included studies, we identified four categories that summarise approaches employed with the aim of achieving rapid evaluation (Table 2):

(1) Use of a methodology designed specifically for rapid evaluation which involved the whole research process

(2) Increasing rapidity by doing less, or using a less time-intensive methodology

(3) Adaptation of a non-rapid evaluation to make this a rapid part of the wider study (e.g., the recruitment stage of a trial or the analysis phase of a qualitative study)

(4) Use of alternative technologies and/or data sources to increase rapidity of an existing evaluation methodology

Some studies used more than one approach to rapidity; (for example an adaptation of a part of a wider study combined with the use of an alternative technology) and are therefore represented in multiple categories.

Below we expand on each category, giving illustrative examples for each.

1. ***Methodology specifically designed for rapid evaluation (rapid methodology)***

The largest number of papers concerned methodological approaches used specifically for rapid evaluation (examples and methodologies detailed in Appendix 2, Supplementary Table 1). These were represented by 117 studies (9-11, 29, 31-46, 48-50, 52-73, 75-88, 90, 92-109, 111-126, 128-136, 139-143, 169, 218-220). Some studies are represented by more than one publication and by supporting methods papers identified from references. Individual study details are in Supplementary Tables 2 -4 (Appendix 2).

Examples include methods described as rapid assessments or rapid assessment studies (82-87, 89, 219), rapid assessment procedures or processes (39, 40); rapid evaluation (58, 82-87, 219); rapid appraisal procedures or processes (54, 56, 62) and rapid ethnographic assessments, ethnographic appraisals or ethnography (106-109, 111, 116-121).

Many of these approaches were closely related while some were explicit adaptations of other rapid approaches, such as rapid appraisal (62), rapid participatory appraisal (50, 103) rapid participatory appraisal with meta-planning (102), qualitative rapid appraisal, rigorous analysis (Q-RARA)(60), rapid assessment and response (RAR) (45), rapid assessment response and evaluation (RARE) (94); focused rapid assessment process (fRAP)(78) and rapid assessment procedure informed clinical (or community) ethnography (RAPICE)(112, 113, 125, 126). A paper based on Rapid Assessment of Avoidable Blindness, an approach specific

For peer review only - http://bmjopen.bmj.com/site/about/guidelines.xhtml

to vision services, was also identified,(77) although most examples of this were excluded because they related to LMIC.

Four studies described themselves as adaptations of rapid assessment processes for specific evaluation purposes (34, 35, 38, 41). Some approaches, such as 'plan, do, study, act', rapid cycle evaluation and rapid feedback evaluation were drawn from areas such as quality improvement and wider implementation science (31-33, 105, 132, 133, 135, 141-143).

Four of the papers examined limitations of a particular methodology, ways in which these limitations could be reduced,(123) or looked at the impact of rapid applications of these methodologies (48, 114, 115). This included highlighting the need for researchers to have received an appropriate level of training in the methodology.

The overarching characteristics of the methodologies designed to increase rapidity were that they were qualitative or mixed methods. None were exclusively quantitative nor were capable of *comparative* assessment of innovations (as is often required for health technology assessment). The actual methods employed in these studies shared core features including: multiple assessment methods (e.g., a combination of interviews of a small number of participants and wider surveys), and triangulation of data from the different approaches, either different methods (e.g. surveys and interviews) or the same approach with different groups (e.g. interviews with different types of stakeholders). The types of evaluation questions addressed in these studies largely focused on the applicability of previous research; acceptability of interventions; barriers and facilitators to intervention implementation or access; and on stakeholder views and experiences.

In addition to the marked increase in the number of studies identified, the principal change we identified in those conducted during the Covid-19 pandemic was the use of online recruitment and videoconferencing software within established methodologies.

2. **Increasing rapidity by doing less, or using a less time-intensive version of an existing methodology**

The second approach to rapid evaluation involved undertaking evaluation on a smaller scale or in a less-intensive way (Table 3); sometimes with overlap with use of an alternative technology (see section 4 below) (61, 148). We identified 28 studies or related methods papers of this type (61, 67, 88, 110, 144-167) (see Appendix 2, Supplementary Tables 2 and 3 for details).

Sometimes authors carried out a smaller study, with fewer sites and/or participants meaning that less data collection and analysis was required. Shortening the data-collection period was also adopted (144-146). One of these studies (147) comprised one of the few comparative studies identified in the review (see section below). Some studies balanced the impact of using smaller samples by employing techniques identified in methods designed specifically for rapid evaluation above, such as stating that they used random or pseudo-

For peer review only - http://bmjopen.bmj.com/site/about/guidelines.xhtml

random participant/site selection, or selected a subsample of participants in such a way to ensure representativeness (148, 163, 166). In other cases, no such measures were reported; such studies accepted – explicitly or implicitly – the trade-off between rapidity and representativeness or reliability of research (164). An alternative approach involved using a less-intensive method resulting in reduced information collected for each participant or site. This included using a briefer form of a research tool (survey, questionnaire or interview proforma) (67, 148, 149, 165). A variation on this was the use of group interviews as a substitute for individual interviews (157). A proposal for an RCT using this principal of doing less at every stage was also identified (167). Updating the search in 2022 identified for the first time studies which used the internet to make their research less time intensive. Examples of this identified as making a study rapid were online surveys, online recruitment, or videoconferencing software for interviews or focus groups (150-156, 158, 159). These are primarily examples of using technology to make research rapid but are included here due to the time-intensity implications of their use.

For peer review only

For peer review only - http://bmjopen.bmj.com/site/about/guidelines.xhtml

Table 3: Approaches to increasing rapidity through less evaluation/less time-intensive evaluation

**Approach employed with aim of increasing rapidity**	**Methods**	**Example**
Shortening response period	Survey; safety monitoring cohort. Survey also used increased number of reminders to improve response rate	Joseph 2003, Demeulemeester 2017, Ghandi-Banga 2018(144-146)
Using an online survey	Survey hosted on webpage and/or social media sites.	Attal 2020, Daviskiba 2021, Elsawy 2022, Hammond 2022; Khubchandani 2021, Talati 2021, Petrella 2021(150-155, 159)
Using telephone or videoconference instead of face-to-face interviews	Interviews. Additional measures included purposive sampling following identification on social media	Williams 2019, Wichmann 2009, Fisher 2021, Srinivasan 2020 (61, 148, 156, 157)
	Focus groups. Use of breakout rooms within a larger meeting on videoconference platform<br>Group interviews instead	Keniston 2022(158)
Using a rapid interview method	Interviews; need to apply rapid method appropriately noted<br>Using a group interview instead of individual interviews<br>Use of rapid data collection methods	Hamilton 2013, Srinivasan 2020, Wanat 2022 (88, 149, 157)
Using fewer participants in interviews	Interviews. Additional measures included random or quasi-random sampling of participants; use of samples selected for representativeness; use of small core group for all stages of project.	Wichmann 2009, Dunbar 2018, Brown-Johnson 2019, Hamaraman 2014, Quintero Romero 2006, Loosier 2010 (110, 148, 160-163)
Using fewer study sites	Random or quasi-random site selection (e.g. housing blocks; in some instances the selection of sites/participants is the same process.	Brown-Johnson 2019, Hamaraman 2014, Cakir 2008, Rodriguez 2021 (161, 162, 165, 166)
Evaluating a small/low volume site	Site audit/evaluation – acknowledged small site volume made rapidity possible	Delaney 2019 (164)
Using a shorter timescale for pre/post data periods	Interrupted time series – non-rapid application of method also used (more pre and post data).	McCarthy 2019 (147)

For peer review only

Using simpler methods and recording less data at fewer timepoints	Asking fewer questions at each time point in a single or repeated measures survey. Use of fewer sites and convenience sampling also reported.<br>Proposal for "Large Simple Randomised Trials" (wide, simple eligibility criteria; central randomization; recording few baseline data; reduced/no follow-up visits; outcome assessments of hard endpoints, preferably by registries). This is proposed for simple short-term treatment for large populations in relation to universal vaccinations.	McDonald 2022, Rodriguez 2021, Hasford 2020 (67, 165, 167)

For peer review only

For peer review only - http://bmjopen.bmj.com/site/about/guidelines.xhtml

3. Adaptation of part of a standard evaluation method

Forty-four studies reported methods they used with the aim of increasing the rapidity of a discrete element or stage of an evaluation, where the evaluation as a whole used standard methods (Table 4) (150-156, 158, 159, 168, 169, 172-179, 182-190, 192-203) (see Appendix 2, Supplementary Tables 2 and 3 for details).

This approach often targeted the earlier stages of an evaluation, including research priority setting (168-171), ethical approval (172), and participant identification and recruitment (173-175, 180, 181). Increased use of online recruitment and data collection was identified following the onset of the Covid-19 pandemic (150-155). Developments in RCT design were also seen over this period (183, 184, 186-189).

At later stages of the evaluation process we identified continuous or contemporaneous collection or data analyses as a means to increase rapidity of evaluation in RCTs and other quantitative study designs (185, 193, 198). In qualitative studies the focus was on rapid methods for data analysis (185, 193-197, 201, 202), including three studies which compared rapid and standard analysis (see below) (194, 196, 197). Here, as in section 2 above there was some overlap with the use of technological innovation to increase rapidity (see section 4 below) (192). This is particularly the case with the use of web-based or virtual technology in specific stages of studies. Here we noted increasing use (from a low base) of methods for rapid analysis of qualitative data in the context of the Covid-19 pandemic (185, 193-196, 201, 202, 206).

For peer review only

For peer review only - http://bmjopen.bmj.com/site/about/guidelines.xhtml

Table 4: Adaptation of a discrete stage of a non-rapid evaluation to make this a rapid part of the wider study

Study stage targeted	Rapid methods	Examples
Priority setting	To improve rapidity of research question prioritisation (applicable to multiple designs). Use of virtual meetings in some instances.	Gray 2017, Newlands 2018, Cowan 2021, Dudovitz 2021 (168-171)
Ethics approval	Intended to reduce time to obtain ethical approval by addressing requirements for multiple approvals (applicable to multiple designs)	Khan 2014 (172)
Recruitment and participation	Reducing time taken to identify participants and complete recruitment through methods to pre-specify or identify potential participants, (primarily in RCTs) including through using participants from previous studies (in surveys); use of near real-time data from Test-and-Trace to identify potential participants in time-sensitive trial.	Bower 2015, Brown 2019, Deane 2017, Corley 2021, Xiang 2021, Wesolowski 2020, Cake 2022 (173-175, 180-183)
	Using multiple methods for recruitment alongside rapid analysis of success for each method. Use of websites or social media to identify and recruit participants for surveys or interview studies (conduct of interview studies noted below)	Bjornson-Benson 1993, Attal 2020, Daviskiba 2021, Elsawy 2022, Hammond 2022, Khubchandani 2021, Talati 2021, Petrella 2021(28, 150-155, 159)
Rapid bilingual appraisal	Reducing barriers to inclusion in rapid evaluation; applicable to multiple study designs where language is a potential barrier	Whelan 2004 (176)
Assessment of barriers to process	Tool for rapid assessment of barriers to process; assessed within an RCT	Alolod 2015, 2016 Traino 2012 (177-179)
Structure of treatment groups/assignment	Proposal for flexible structuring of treatment assignment and use of single control group for multiple potential vaccine candidates in multi-stage RCT<br>Implementation of platform trials for multiple intervention assessment	Moodie 2013, Douglas 2022, Griffiths 2021, Murray 2022, Short 2022, RECOVERY (167, 184, 186-191)
Nesting of additional studies	Continuous rapid data analysis in realist study embedded in standard RCT	Hensel 2016 (185)

For peer review only

For peer review only - http://bmjopen.bmj.com/site/about/guidelines.xhtml

Data collection	Bespoke reporting platform; cohort study protocol to rapidly assess vaccines as well as epidemiology of disease	Simpson 2015 (192)
	Data tracking and rapid continuous feedback to participants (My Own Health Report). Designed to provide continuous collection and analysis of data in a pragmatic RCT	Glasgow 2014 (193)
	Use of multiple publicly available data sets to evaluate vaccine impact	Li 2021(198)
	Use of online, web-based methods to interview individual participants as alternative or optional method or to conduct focus groups	Fisher 2021, Keniston 2022 (156, 158)
Data analysis	Rapid analysis of data with or without other rapid stages; used in qualitative (comparative) and mixed methods studies; embedded realist study; cohort study; focus group<br>Specific methods include Rapid Identification of Themes from Audio recordings (RITA). Other methods noted include measures to improve rigour (e.g. triangulation, reflexivity, peer examination).	Taylor 2018, Burgess-Allen 2010, Glasgow 2014, Hensel 2016, Nevedal 2021, Vazquez 2022, Rolf 2021, Lewsinki 2021, Tan 2021, Elwy 2021, Gale 2019(185, 193-197, 199-203)

For peer review only - http://bmjopen.bmj.com/site/about/guidelines.xhtml

4. Using alternative technology or data source

The use of different or newer technology to rapidly acquire and/or analyse routine data was another approach to making evaluation more rapid (Table 5). As we have noted there is some overlap between this and the classifications above, but our synthesis considered this separately as it represents a technological streamlining of an existing process rather than an adaptation of the research design (61, 148). Perhaps unsurprisingly this is an area in which we see the emergence of substantial numbers of studies and new adaptations of methods with the response to the Covid-19 pandemic.

The 28 studies discussed here involved using technology to automate or otherwise try to improve rapidity of one or more of data collection, collation, or analysis (61, 140, 148, 150-156, 158, 159, 183, 192, 204-215, 217). Studies which involved developing, acquiring, sharing or using existing data sets to enable modelling of effectiveness were also included (30, 140, 183, 207, 210, 213-217). Seven studies were undertaken in the context of monitoring of uptake and safety profiles of vaccinations even prior to Covid-19. For study details see Supplementary Tables 2 and 3 (Appendix 2).

For peer review only

Table 5: Use of an alternative technology or data source

Study authors' suggested rapid innovation	Research design	Impact/intention of method	Example(s)
New digital systems	Registry study<br><br><br><br><br>Surveys	Introduction of new computerised systems to assess vaccination coverage of routine immunisation programmes; computerised child registers also became available.<br><br>Computer-assisted telephone surveys. Authors stated that it enabled more timely feedback to co-ordinators of district vaccination programmes and rapid identification of random samples for interview<br>Online surveys; use of wholly online surveys hosted on webpages or social media to recruit participants and conduct survey<br>Computer-based rapid recruitment<br>Computer-based rapid data-collection/analysis	Williams 2019, Wichmann 2009, Begg1989, Attal 2020, Daviskiba 2021, Elsawy 2022, Hammond 2022, Khubchandani 2021, Talati 2021, Petrella 2021(61, 148, 150-155, 159, 204)
Use of video conferencing technology	Assessments or evaluations using interviews or focus groups	Videoconference platforms used to enable interviews or focus groups; in some cases multiple sessions were conducted simultaneously	Fisher 2021, Keniston 2022(156, 158)
Using bespoke data platform	Cohort	Nationally representative sample assessed using linking of multiple data sources from clinical practice, laboratory results and death certification. Designed to assess pandemic influenza reporting and vaccination impact; plan to be implemented for future influenza pandemic	Simpson 2015 (192)
Secure web-based tool for pharmacovigilance	Cohort	Secure web-based tool for small number of set fields focused on single medications in hospice context using rapid prospective reporting at agreed time-points to allow rapid aggregation of data on small numbers of participants from multiple centres	Currow 2012 (205)

For peer review only - http://bmjopen.bmj.com/site/about/guidelines.xhtml

Automation of data collection	Registry study	Use of hospital routine data collection to populate registry with validation to analyse costs for two different methods of triage of designated patient group	Noack 2018, Price 2022 (140, 207)
Automation of data processing	Registry study	Use of regional/organisational vaccine information system to rapidly assess influenza vaccine campaign safety and coverage	Alguacil-Ramos 2019, CDC 2005(208, 211)
Use of existing data sets	Registry-based studies	Use of data from (e.g.) an insurance database or hospital data registry to populate a comparison of a specified adverse event or to analyse costs.<br>Use of real time data within an organisation to create a Learning Healthcare System.<br>Using a national database for near real-time recruitment in time-sensitive treatment trial.<br>Methods of data-sharing or promotion of data-sharing, including creation of publicly accessible rapidly updated data repositories.	Gagne 2013, Noack 2018, Agaku 2022, Cake 2022, Carrillo 2022, Dambha-Miller 2021, Harrison 2021, Talwai 2021, Saunders 2022, Price 2022 (183, 207, 209, 217)<br>(140, 212-216)
Use of technology to generate data sets	Qualitative study	Use of orchestrated Twitter chat to generate data set for qualitative analysis	Shimkhada 2021(206)
Simulation of impact of vaccination using existing health care data sets	Simulation study	Use of established data sets to run simulations on how vaccine coverage and population characteristics impact ability to detect safety signals for influenza vaccinations. The authors recommended this be used only as a method to detect signals and generate hypotheses.	Dodd 2018 (210)

For peer review only

For peer review only - http://bmjopen.bmj.com/site/about/guidelines.xhtml

Studies comparing a rapid with a non-rapid method

We found only four studies which compared a rapid evaluation method with a non-rapid method, and we did not find any comparisons of different types of rapid evaluations. Three of the studies focused on comparing rapid versus traditional analysis of qualitative data.

One study compared the use of rapid evaluation with standard evaluation of an enhanced health programme in care homes. The study was described as assessing rapid evaluation but the rapid evaluation itself took 2.5 years (147). Nevertheless, this was the only explicit comparison we found. This study assessed the cost consequences of implementing the programme and compared the use of shorter and longer-term interrupted time-series (ITS) analyses, using shorter and longer time-series of aggregated data (147). The authors found that the longer-term analyses yielded a different interpretation, an improved model fit and greater precision; and they considered that the shorter-term analyses produced results likely to be misleading. The time period required for acceptable internally valid ITS based studies is contested more generally (221-224).

A second study compared rapid with usual qualitative analysis in an evaluation of a home birth service in the UK (194). The study authors reported on the total time taken in the data-management process as well as the analysis component. The agreement between the findings and recommendations of the two processes was also considered. The authors concluded that rapid analysis delivered a modest timesaving which may have been greater if there had been greater concordance between the characteristics of the two research teams. They also reported substantial overlap in findings between the two analysis approaches, although their recommendations diverged more. The two analyses were undertaken by different research teams which had differing levels of contextual experience, different stakeholder audiences and differing opportunities for analysis discussion and this was identified as a limitation in the study, together with the fact that rapid analysis was a novel approach for the researchers involved. The study authors concluded that rapid analysis may potentially deliver valid findings while taking less time but recommended further comparisons using additional data sets with more comparable research teams.

Two studies compared rapid with standard qualitative analysis within the US Veterans Health Administration (196, 197). One study looked at an evaluation of a programme which seeks to identify and diffuse evidence-based interventions (196), while the other evaluated a specific evidence-based strategy in opioid prescribing (197). In each case authors used the Consolidated Framework for Implementation Research and compared the use of a rapid deductive approach to analysis using notes and audio recordings to a traditional deductive approach using transcripts.

For the wider programme authors reported that the final summaries produced by the two approaches were “quite similar”; they judged that both approaches allowed them to identify and describe the factors influencing implementation, which was the goal of their

For peer review only - http://bmjopen.bmj.com/site/about/guidelines.xhtml

evaluation, but that the rapid approach allowed formal results to be generated and shared more quickly (196). In the evaluation of the opioid prescribing programme the authors reported that findings from the two analyses were consistent (197).

DISCUSSION

Summary

This scoping review identified a lack of clarity in approaches described as 'rapid evaluation' in primary research. The approaches to rapid evaluation which we identified could be grouped into four broad categories (1) studies using specific methodologies designed for rapid evaluation; (2) studies which carried out less evaluation or less intensive evaluation; (3) studies which used rapid methods only for a particular stage of evaluation; and (4) studies which used technology to increase rapidity. There was very little comparative research on the impact of using rapid rather than non-rapid evaluation (147, 194, 196, 197).

It is notable that we did not identify many rapid evaluation methodologies clearly designed to assess the comparative effectiveness of innovations. This was reflected in the fact that the recent systematic review which focussed on rapid evaluation methodologies more narrowly included only 12 studies despite considering studies from LMIC as well as high income countries (9). Studies using bespoke, rapid methodologies used qualitative or mixed methods and mainly assessed aspects of user experience and acceptability along with associated barriers and facilitators, and the implementation of innovations in particular contexts. This may reflect the types of questions which are considered suitable for rapid evaluation and the impetus to develop rapid evaluation methods in these areas of research, but it may also reflect conceptions of what evaluation is possible within a limited timeframe**.**

Impact of Covid-19 pandemic

Our initial search was conducted in February 2020 and our update search in September 2022, meaning that we were able to identify the impact of the Covid-19 pandemic on the published literature to date. Ninety of the 162 included studies were identified from the update search in September 2022. The fact that over half of all included studies were indexed since the onset of the pandemic indicates a substantially increased level of rapid research publication.

This increase in publication numbers has coincided with the accelerated development of some approaches to rapid evaluation previously identified as proposals or isolated examples of methods. The use of "platform" designs for flexible participant allocation within RCTs is exemplified by the RECOVERY trial (190) but we also found recent studies using this approach in non-Covid contexts such as oncology. The use of rapid analysis methods within qualitative studies also showed more examples, methodological developments and assessment of the impact of using rapid compared to conventional methods. The impact of Covid-19 is also clear in participation and recruitment phases of studies. We identified an

For peer review only - http://bmjopen.bmj.com/site/about/guidelines.xhtml

example of linking of almost real-time datasets to facilitate recruitment in a trial of a time-sensitive intervention. This study used data from the UK NHS test-and-trace programme to identify high risk people who had recently recorded a positive test for Covid-19 in the community for recruitment to a treatment trial (183). We also see the first examples of web-based methods used to rapidly generate data for evaluation (206).

We also identified papers relating to infrastructure designed to increase research rapidity and coordination. This involved the creation of data repositories which can incorporate real world data from different sources, thereby supporting further studies which use existing data sets to enable rapid evaluation, and triangulation of data from different sources. (213-215, 225) We also identified methodological work to increase the comparability and useability of data entering such repositories (212). The importance of infrastructure and of leveraging existing resources and relationships in supporting rapid evaluation more widely was the subject of a number of commentaries identified by our searches but not eligible for inclusion (226-229).

However, most of the newly identified studies represented one of two approaches to rapid evaluation. There was an increase in the use of established rapid evaluation methodologies such as rapid appraisal. Many of these recent studies also involved an adaptation of the processes used for remote working approaches with online recruitment and videoconferencing incorporated into the established methodologies. We also found multiple studies which used remote working approaches (to all or part of a study) as a less time intensive and technological approach to make a study rapid. Examples of this were use of online surveys, online recruitment, or videoconferencing software for interviews or focus groups. The adoption of these methods within and outside bespoke methodologies may have been driven by necessity but is likely to represent a permanent change in the rapid evaluation toolkit. The impact using remote methods on research has not yet been explored.

For peer review only - http://bmjopen.bmj.com/site/about/guidelines.xhtml

Limitations of the review

We registered our protocol at an early stage of the review process and specified in it that we would adopt a flexible and iterative approach to the review. We used this flexibility to narrow the scoping review we conducted in several ways in view of the very large number of identified papers. We addressed only the two questions we considered central to mapping the evidence around rapid evaluation and limited the eligible studies to those concerned with primary evaluation research in high income countries, excluding regulatory processes as well as rapid reviews. We acknowledge that there is the potential for these decisions to have been driven by the nature of the identified literature, but we prespecified this flexible approach to produce a review that would be relevant to the context of the NHS in England, while remaining comprehensive enough to capture relevant research methods. We have documented this in our methods and results to ensure transparency about the review processes.

The ongoing work by others in documenting and developing methods for rapid reviews means that we are confident that our decision to focus on primary research, where there is much less evidence synthesis or guidance is justified. Our decision to exclude reports from a regulatory context has the potential to exclude relevant studies, particularly given that the pivotal role of the UK National Institute for Health and Care Excellence (NICE) in developing approaches to such evaluation. However, after coding the identified studies it was apparent that rapid methods related primarily to evidence synthesis. Future research could usefully explore the impact of a rapid or truncated regulatory process on the types of evidence considered sufficient for approvals, and the implications of this for primary evaluation.

A related limitation concerns the decision to limit our inclusion criteria to reports from high income countries, as we may have omitted methods for rapid evaluation which might be transferable to the UK context. Firstly, we identified the use of lot-quality surveys (a method of using small sample sizes to determine if areas have achieved a specified target) as a method of doing research with fewer participants; this was only represented in studies from LMIC (166, 230). We also found an example of a method of increasing rapidity by making part of a study more rapid which was only identified in LMIC contexts but may be particularly relevant to disadvantaged or marginalised participants in the UK. Six studies used preparatory methods to ensure informed consent so that enrolment for subsequent studies could be undertaken rapidly because necessary groundwork had been undertaken (176, 231-235). We note that some of our excluded studies from LMIC described their methods as 'rapid' but with limited information on what promoted rapidity. Our initial assessment suggested that evaluations completed within a period of weeks, or a few months was what qualified them as rapid in the eyes of the authors. It is possible that a systematic review with wider inclusion criteria and the resources to contact authors may have identified specific methods which would have relevance to UK settings, but which are not represented in either our included studies or the excluded studies summarised here.

For peer review only - http://bmjopen.bmj.com/site/about/guidelines.xhtml

Due to the high volume of identified literature at the initial and second stages of the review process we did not use duplicate screening and coding by two independent researchers for all studies. We acknowledge that this may have increased the risk of error or bias in the review processes. However, we screened and coded subsets of the records at each stage in duplicate and found very high levels of agreement, so we do not believe that the impact is likely to be substantial. This is particularly the case as we consulted a second reviewer in cases of uncertainty for other records, both in screening and in coding, meaning that consensus was used where judgments were difficult.

Our review complements a recently published systematic review of rapid evaluations (9). Our review had a wider methodological scope and included a correspondingly larger number of studies (162 studies compared to 12), but identified some similar themes, such as variable labelling of rapid evaluations, and similar strategies to achieve rapidity. Importantly, the previous review highlighted the importance of the 'trustworthiness' of rapid designs, while we were able to explore the limited but critical evidence base comparing rapid and non-rapid methods. We have included both this systematic review and a subset of the primary studies which it included, however this corresponds to only eight primary studies across different sections of our results, so we believe the impact of potential double-counting to be minimal (9).

Because this is a scoping review we did not conduct quality assessment of the identified studies; we therefore document the nature of the research available rather than assessing its reliability (24, 25). We do not attempt to evaluate the success or otherwise of methods proposed as enabling rapid evaluation but have accepted study authors' own assessments of the usefulness or success of their methods. A further limitation of this review is that we were unable to identify (and therefore include) studies that may have carried out work which represents rapid evaluation but have failed to describe it as such (for example, retrospective quantitative analyses). This might bias the mapping of studies identified if particular types of studies exhibit differential propensity to report themselves as rapid.

Conversely, we identified some evaluations which the study authors described as rapid, but which might not commonly be considered as rapid, except in comparison to an even more detailed and lengthy standard process. We explicitly excluded pilot studies (often RCTs) because these were considered to be assessing feasibility rather than undertaking evaluation (236). This may have contributed to a skewing of the literature identified towards non-quantitative methods, but the pilot studies we did identify did not label themselves as being rapid studies e.g. (237-239). We are aware that we may therefore have omitted some examples of studies which would meet some definitions of rapid. However, these limitations would not explain the relative paucity of studies comparing rapid to alternative methods in the identified literature, and this appears to be a genuine evidence gap.

For peer review only - http://bmjopen.bmj.com/site/about/guidelines.xhtml

Interpretation of the studies

The drive towards rapid evaluation raises tensions for those commissioning and delivering research. Evaluation studies aim to achieve rigour (broadly, internal and external validity, or their qualitative equivalents) and scope (answering a range of questions, including those relating to access, effectiveness, cost effectiveness, acceptability, equity, and implementation). In most circumstances these must be achieved under constraints of cost and time. The greater the scope of the project, and the greater the protection against validity threats, the higher is the cost and time. These known trade-offs are being explored by organisations such as BRACE and RSET (240).

Occasionally, researchers can trade time against cost. For example, national recruitment drives (such as those under COVID in trials such as RECOVERY) have maintained high levels rigour under time pressure by deploying significant resources (190). Some of the factors delaying evaluation delivery can be managed through additional resources. However, some, such as long-term follow up, cannot be managed in that way. In regulatory contexts these trade-offs may be addressed through mechanisms of post-marketing surveillance (241), but this only applies to a subset of innovations such as pharmaceuticals or medical devices.

Where studies cannot be accelerated through additional resource, or where costs are constrained, rapid evaluation essentially involves trading off rigour or scope against time. Some of those trade- offs can be profound. For example, a study of an innovation might eschew questions of comparative effectiveness and cost-effectiveness entirely and restrict the scope of the study to issues of staff acceptability and implementation. This seemed to be the approach adopted in some of the studies identified in the review. Quantitative rapid evaluations are more likely to evaluate impacts of innovations on short-term indicators (such as processes of care) which are often proxies for outcomes where longer-term follow-up is required. Therefore, the limitations of such approaches are likely to depend on the strength and consistency of the relationship between the proxy and the ideal longer-term outcome. Some decision-making frameworks (such as the NICE digital framework) specifically identify some interventions where such trade-offs are legitimate (242). Contextual pressures (such as the pandemic) may force trade-offs to ensure timely decisions, but they have also been the impetus for improved infrastructure which may support ongoing rapid research (225). We noted concern that ethics and stakeholder engagement should not be impacted negatively by increased rapidity (243-245).

Other trade-offs will be more marginal. For example, a decision maker might accept a smaller sample (and hence a lower level of quantitative precision) or more selective recruitment (at some cost in external validity). It is also the case that reduced statistical power related to using shorter post-intervention time periods might be at least partly offset by using a comparative design (such as difference-in-differences) rather than interrupted

For peer review only - http://bmjopen.bmj.com/site/about/guidelines.xhtml

time series, since estimates from comparative designs are based on more information than non-comparative approaches. Such trade-offs impact on rigour and scope in a more limited way that might be more acceptable. Larger trade-offs will be potentially easier to assess in terms of their impact – for example, eschewing questions of comparative effectiveness will, or should, restrict what can be claimed about an innovation. Some trade-offs are more difficult to judge in terms of their impact.

The role of rapid evaluation methodology is to reduce impacts on rigour and scope as far as possible, while maintaining the benefits in terms of time. We identified several examples of these in the literature, although there was limited comparative work on their success (147, 194). Since conducting prospective comparisons of rapid and non-rapid evaluation methods is time-consuming and resource intensive, it may be possible to use retrospective analysis of existing data to explore the impact of using shorter analysis periods. For example, it would be possible to conduct a sensitivity analysis of truncated sets of timepoints in an interrupted time series, and to compare the results to the full analysis. Performing such analyses on several sets of data from different studies would allow assessment of whether there was a consistent impact on results but would be possible with only a limited set of study designs. In this the studies relating to regulatory decision-making, excluded from this review, may provide useful design features.

Future research

There is a need for clarity and consistency in terms of what constitutes rapid evaluation and, in particular, what differentiates rapid from non-rapid evaluation. Consensus on definitions and more consistent labelling will be important in allowing ongoing assessment of this developing field. We suggest that better description would involve clarity about what aspects of the research process were conducted more rapidly, when this occurred in the research process, and what the potential impacts could be.

The development of specific rapid methodologies and technologies is needed to provide a toolkit for research teams to meet the challenges of rapid evaluation.

The development of new, rapid methodologies needs to be matched by comparative work on their advantages and disadvantages, such as the impact on uncertainty for decision-makers. Such comparative work will be challenging, adding cost and complexity in a context where commissioners and researchers are seeking to reduce both and will require innovative methodologies, such as testing rapid methods nested within 'standard' projects e.g. (185, 193) to maximise efficiency.

Figure Legends

For peer review only - http://bmjopen.bmj.com/site/about/guidelines.xhtml

Figure 1: PRISMA diagram showing study flow for the review

For peer review only

For peer review only - http://bmjopen.bmj.com/site/about/guidelines.xhtml

Declarations

Ethics approval and consent to participate

This is a scoping review of previously published studies so ethical approval and consent to participate are not applicable.

Consent for publication

Not applicable

Availability of data and materials

This is a scoping review of previously published studies, so all the data used in the review is already in the public domain. Full lists of all studies considered after initial screening are available from the corresponding author on reasonable request, together with the dataset generated by the review process. Studies whose data substantially contributed to the review are cited in the bibliography.

Competing interests

The authors declare that they have no competing interests.

Funding

This research was funded by the National Institute for Health and Care Research Applied Research Collaboration Greater Manchester. The views expressed in this publication are those of the author(s) and not necessarily those of the National Institute for Health and Care Research or the Department of Health and Social Care.

Authors' contributions

GN and TM carried out the review process with input from JD, PB, PW and NC. All authors contributed to interpretation of the data. GN wrote the first draft of the manuscript text. JD, TM, PB, PW and NC reviewed and substantially edited and revised the manuscript.

Acknowledgments

The authors are grateful to Dr Su Golder for design and implementation of the search strategy and to Sophie Bishop for the search update.

For peer review only

REFERENCES

1. Johnson GA, Vindrola-Padros C. Rapid qualitative research methods during complex health emergencies: A systematic review of the literature. Soc Sci Med. 2017;189:63-75.
2. Vindrola-Padros C. Can We Re-Imagine Research So It Is Timely, Relevant and Responsive? Comment on "Experience of Health Leadership in Partnering with University-Based Researchers in Canada: A Call to 'Re-Imagine' Research". International Journal of Health Policy and Management. 2021;10(3):172-5.
3. vindrola-Padros C, Johnson GA. Rapid Techniques in Qualitative Research: A Critical Review of the Literature. . Qualitative Health Research. 2020;30(10):1596-604.
4. Tricco AC, Antony J, Zarin W, Strifler L, Ghassemi M, Ivory J, et al. A scoping review of rapid review methods. BMC Medicine. 2015;13 (1) (no pagination)(224).
5. Tricco AC, Zarin W, Antony J, Hutton B, Moher D, Sherifali D, et al. An international survey and modified Delphi approach revealed numerous rapid review methods. Journal of Clinical Epidemiology. 2016;70:61-7.
6. Cooper K, Martyn-St James M, Pandor A, Kaltenthaler E, Wong R. The impact of searching fewer databases in health technology assessment rapid reviews. International Journal of Technology Assessment in Health Care. 2017;33 (Supplement 1):49-50.
7. Cooper K, Martyn-St James M, Pandor A, Wong R, Kaltenthaler E. Selecting rapid review methods for health technology assessment. International Journal of Technology Assessment in Health Care. 2017;33 (Supplement 1):225-6.
8. Patnode CD, Eder ML, Walsh ES, Viswanathan M, Lin JS. The Use of Rapid Review Methods for the U.S. Preventive Services Task Force. American Journal of Preventive Medicine. 2018;54(1 Supplement 1):S19-S25.
9. Vindrola-Padros C, Brage E, Johnson GA. Rapid, Responsive, and Relevant?: A Systematic Review of Rapid Evaluations in Health Care. American Journal of Evaluation. 2021;42(1):13-27.
10. McNall M, Foster-Fishman PG. Methods of Rapid Evaluation, Assessment, and Appraisal. American Journal of Evaluation. 2007;28(2):151-68.
11. Beebe J. Rapid assessment and response: Sound methodology for producing timely responses. International Journal of Drug Policy. 2000;11(1-2):29-31.
12. Bonten TN, Rauwerdink A, Wyatt JC, Kasteleyn MJ, Witkamp L, Riper H, et al. Online Guide for Electronic Health Evaluation Approaches: Systematic Scoping Review and Concept Mapping Study. J Med Internet Res. 2020;22(8):e17774.
13. Fynn JF, Hardeman W, Milton K, Jones AP. A scoping review of evaluation frameworks and their applicability to real-world physical activity and dietary change programme evaluation. BMC Public Health. 2020;20(1):1000.
14. Vindrola-Padros C, Chisnall G, Cooper S, Dowrick A, Djellouli N, Symmons SM, et al. Carrying Out Rapid Qualitative Research During a Pandemic: Emerging Lessons From COVID-19. Qualitative Health Research. 2020;30(14):2192-204.
15. Greenhalgh T, Robert G, MacFarlane F, Bate P, Kyriakidou O. Diffusion of Innovations in Service Organizations: Systematic Review and Recommendations The Milbank Quarterly. 2004;82(4):581-629.
16. Norman G, Dumville JC, Wilson P, Cullum NA, Mason T, Bower P. Scoping review of rapid evaluations https://osf.io/np72f/: Open Science Framework 2020 [
17. Tricco AC, Lillie E, Zarin W, O'Brien KK, Colquhoun H, Levac D, et al. PRISMA Extension for Scoping Reviews (PRISMA-ScR): Checklist and Explanation. Ann Intern Med. 2018;169(7):467-73.
18. Garritty C, Gartlehner G, Kamel C, King VJ, Nussbaumer-Streit B, Stevens A, et al. Cochrane Rapid Reviews. Interim guidance from the Cochrane Rapid Reviews Methods Group. http://methods.cochrane.org/sites/methods.cochrane.org.rapidreviews/files/uploads/cochrane_rr_-_guidance-23mar2020-final.pdf: Cochrane Collaboration; 2020.

For peer review only - http://bmjopen.bmj.com/site/about/guidelines.xhtml

19. Garritty C, Gartlehner G, Nussbaumer-Streit B, King VJ, Hamel C, Kamel C. Cochrane Rapid Reviews Methods Group offers evidence-informed guidance to conduct rapid reviews. Journal of Clinical Epidemiology. 2021;130:13-22.
20. Reynen E, Robson R, Ivory J, Hwee J, Straus SE, Pham B. **A retrospective comparison of systematic reviews with same-topic rapid reviews**. Journal of Clinical Epidemiology. 2018;96(23-34).
21. Hamel C, Michaud A, Thuku M, Skidmore B, Stevens A, Nussbaumer-Streit B. Defining rapid reviews: a systematic scoping review and thematic analysis of definitions and defining characteristics of rapid reviews. . Journal of Clinical Epidemiology 2021;129(74-85).
22. Bank W. The world by income and region https://datatopics.worldbank.org/world-development-indicators/the-world-by-income-and-region.html: The World Bank; 2020 [
23. Team TE. EndNote. Endnote 20 ed. Philadelphia, PA: Clarivate; 2013.
24. Arksey H, O'Malley L. Scoping studies: towards a methodological framework. International Journal of Social Research Methodology. 2005;8(1):19-32.
25. Levac D, Colquhoun H, O'Brien K. Scoping studies: advancing the methodology. Implement Sci. 2010;5:69.
26. Hoffmann TC, Glasziou PP, Boutron I, Milne R, Perera R, Moher D, et al. Better reporting of interventions: template for intervention description and replication (TIDieR) checklist and guide. BMJ : British Medical Journal. 2014;348:g1687.
27. Aspray TJ. Rapid assessment methods used for health-equity audit: diabetes mellitus among frail British care-home residents. Public Health. 2006;120(11):1042-51.
28. Bjornson-Benson WM, Stibolt TB, Manske KA, Zavela KJ, Youtsey DJ, Sonia Buist A. Monitoring recruitment effectiveness and cost in a clinical trial. Controlled Clinical Trials. 1993;14(2, Supplement):52-67.
29. Keith RE, Crosson JC, O'Malley AS, Cromp D, Taylor EF. Using the Consolidated Framework for Implementation Research (CFIR) to produce actionable findings: a rapid-cycle evaluation approach to improving implementation. Implement Sci. 2017;12(1):15.
30. Gagne JJ, Bykov K, Najafzadeh M, Choudhry NK, Martin D, Kahler KH, et al. Prospective benefit-risk monitoring of new drugs for rapid assessment of net favorability in electronic healthcare data. Pharmacoepidemiology and Drug Safety. 2015;1):38-9.
31. Skillman M, Cross-Barnet C, Friedman Singer R, Rotondo C, Ruiz S, Moiduddin A. A Framework for Rigorous Qualitative Research as a Component of Mixed Method Rapid-Cycle Evaluation. Qualitative Health Research. 2018;29(2):279-89.
32. McNall MA, Welch VE, Ruh KL, Mildner CA, Soto T. The use of rapid-feedback evaluation methods to improve the retention rates of an HIV/AIDS healthcare intervention. Evaluation and Program Planning. 2004;27(3):287-94.
33. Zakocs R, Hill JA, Brown P, Wheaton J, Freire KE. The Data-to-Action Framework: A Rapid Program Improvement Process. Health Educ Behav. 2015;42(4):471-9.
34. Fergus CJ, Chinn DJ, Murray SA. Assessing and improving out-of-hours palliative care in a deprived community: a rapid appraisal study. Palliative Medicine. 2010;24(5):493-500.
35. Kieber-Emmons A, Crabtree BF, Miller W. A new mixed methods approach to uncover multi-level barriers and facilitators of cancer survivorship. Journal of Clinical Oncology Conference. 2018;36(7).
36. Wright A, Sittig DF, Ash JS, Erickson JL, Hickman TT, Paterno M, et al. Lessons learned from implementing service-oriented clinical decision support at four sites: A qualitative study. International Journal of Medical Informatics. 2015;84(11):901-11.
37. Manderson L, Aaby P. An epidemic in the field? Rapid assessment procedures and health research. Social Science and Medicine. 1992;35(7):839-50.
38. McMullen CK, Ash JS, Sittig DF, Bunce A, Guappone K, Dykstra R, et al. Rapid assessment of clinical information systems in the healthcare setting: an efficient method for time-pressed evaluation. Methods of information in medicine. 2011;50(4):299-307.

For peer review only - http://bmjopen.bmj.com/site/about/guidelines.xhtml

39. Needle RH, Trotter IRT, Goosby E, Bates C, Von Zinkernagel D. Methodologically sound rapid assessment and response: Providing timely data for policy development on drug use interventions and HIV prevention. International Journal of Drug Policy. 2000;11(1-2):19-23.
40. Needle RH, Trotter RT, 2nd, Singer M, Bates C, Page JB, Metzger D, et al. Rapid assessment of the HIV/AIDS crisis in racial and ethnic minority communities: an approach for timely community interventions. American Journal of Public Health. 2003;93(6):970-9.
41. Holdsworth LM, Safaeinili N, Winget M, Lorenz KA, Lough M, Asch S, et al. Adapting rapid assessment procedures for implementation research using a team-based approach to analysis: a case example of patient quality and safety interventions in the ICU. Implementation Science. 2020;15(1):12.
42. Peiro R. Rapid appraisal methodology for 'health for all' policy formulation analysis. 2003.
43. Peiro R, Alvarez-Dardet C, Plasencia A, Borrell C, Colomer C, Moya C, et al. Rapid appraisal methodology for 'health for all' policy formulation analysis. Health Policy. 2002;62(3):309-28.
44. Fitch C, Rhodes T, Stimson GV. Origins of an epidemic: The methodological and political emergence of rapid assessment. International Journal of Drug Policy. 2000;11(1-2):63-82.
45. Stimson GV, Fitch C, DesJarlais D, Poznyak V, Perlis T, Oppenheimer E, et al. Rapid assessment and response studies of injection drug use: knowledge gain, capacity building, and intervention development in a multisite study. American Journal of Public Health. 2006;96(2):288-95.
46. Fitch C, Stimson GV, Rhodes T, Poznyak V. Rapid assessment: an international review of diffusion, practice and outcomes in the substance use field. Soc Sci Med. 2004;59(9):1819-30.
47. Rhodes T, Stimson GV, Fitch C, Ball A, Renton A. Rapid assessment, injecting drug use, and public health. The Lancet. 1999;354(9172):65-8.
48. Campbell J. A critical appraisal of participatory methods in development research. International Journal of Social Research Methodology. 2002;5(1):19-29.
49. Dale J, Shipman C, Lacock L, Davies M. Creating a shared vision of out of hours care: using rapid appraisal methods to create an interagency, community oriented, approach to service development. Bmj. 1996;312(7040):1206-10.
50. Murray SA. Listening to local voices : adapting rapid appraisal to assess health and social needs in general practice. 1994.
51. Murray SA, Tapson J, Turnbull L, McCallum J, Little A. Listening to local voices: Adapting rapid appraisal to assess health and social needs in general practice. British Medical Journal. 1994;308(6930):698-700.
52. Harvey HD, Fleming P, Patterson M. A rapid appraisal method for reviewing the effectiveness of workplace smoking policies in large and medium sized organisations. Journal of The Royal Society for the Promotion of Health. 2001;121(1):50-5.
53. Harvey HD, Fleming P. A rapid appraisal method for the selection and pre-testing of environmental health leaflets. Journal of The Royal Society for the Promotion of Health. 2000;120(2):112-6.
54. Green J, Perkins C, Steinbach R, Edwards P. Reduced street lighting at night and health: A rapid appraisal of public views in England and Wales. Health Place. 2015;34:171-80.
55. Beebe J, Given L. Rapid assessment process. The sage encyclopedia of qualitative research methods

Thousand Oaks, California: SAGE Publications; 2012.
56. Rowa-Dewar N, Ager W, Ryan K, Hargan I, Hubbard G, Kearney N. Using a rapid appraisal approach in a nationwide, multisite public involvement study in Scotland. Qualitative Health Research. 2008;18(6):863-9.
57. Murphy A, Chikovani I, Uchaneishvili M, Makhashvili N, Roberts B. Barriers to mental health care utilization among internally displaced persons in the republic of Georgia: a rapid appraisal study. BMC health services research. 2018;18(1):306.

For peer review only - http://bmjopen.bmj.com/site/about/guidelines.xhtml

58. Foreman-Mackey A, Bayoumi AM, Miskovic M, Kolla G, Strike C. 'It's our safe sanctuary': Experiences of using an unsanctioned overdose prevention site in Toronto, Ontario. International Journal of Drug Policy. 2019;73:135-40.
59. Guise A, Witzel TC, Mandal S, Sabin C, Rhodes T, Nardone A, et al. A qualitative assessment of the acceptability of hepatitis C remote self-testing and self-sampling amongst people who use drugs in London, UK. BMC Infectious Diseases. 2018;18 (1) (no pagination)(281).
60. Phillips CB, Dwan K, Hepworth J, Pearce C, Hall S. Using qualitative mixed methods to study small health care organizations while maximising trustworthiness and authenticity. BMC Health Services Research. 2014;14:559.
61. Williams SP, Kinsey J, Carry MG, Terry L, Wells J, Kroeger K. Get In, Get Tested, Get Care: STD Services in Urban Urgent Care Centers. Sexually Transmitted Diseases. 2019;46(10):648-53.
62. Hamilton S. Felt needs assessment of mental health (rapid service appraisal): Barking and Havering. (Public health research report, ISSN 09689818 ; no 11). 1993:31.
63. Aspray TJ, Nesbit K, Cassidy TP, Hawthorne G. Rapid assessment methods used for health-equity audit: Diabetes mellitus among frail British care-home residents. Public Health. 2006;120(11):1042-51.
64. Livorsi D, Knobloch MJ, Blue LA, Swafford K, Maze L, Riggins K, et al. A rapid assessment of barriers and facilitators to safety culture in an intensive care unit. Int Nurs Rev. 2016;63(3):372-6.
65. Solomon P, Tennille J, Lipsitt D, Plumb E, Metzger D, Blank MB. Rapid assessment of existing HIV prevention programming in a Community Mental Health Center. Journal of Prevention and Intervention in the Community. 2006;33(1-2):137-51.
66. Im H, George N. Impacts of COVID-19 on Refugee Service Provision and Community Support: A Rapid Assessment during the Pandemic. Social Work in Public Health. 2022;37(1):84-103.
67. McDonald R, Eide D, Abel-Ollo K, Barnsdale L, Carter B, Clausen T, et al. A rapid assessment of take-home naloxone provision during COVID-19 in Europe. International Journal of Drug Policy. 2022;107:103787.
68. Minoyan N, Hoj SB, Zolopa C, Vlad D, Bruneau J, Larney S. Self-reported impacts of the COVID-19 pandemic among people who use drugs: a rapid assessment study in Montreal, Canada. Harm Reduction Journal. 2022;19(1):38.
69. Gomez-Ramirez O, Medeiros P, Wainer R, Iyamu I. Does the 'canada COVID-19 alert' app stand up to critical scrutiny? a rapid qualitative assessment. BMJ Open. 2021;11(SUPPL 1):A5.
70. Apter A, Carroll JK, Cardet JC, Cohen R, Colon-Moya AD, Ericson B, et al. Nebulizer Use by Black and Latinx Adults with Moderate to Severe Asthma. Journal of Allergy and Clinical Immunology: In Practice. 2022;10(2):517-24.e2.
71. Renfro CP, Rome Z, Gatwood J, Hohmeier KC. Use of Rapid Assessment Procedures when analyzing qualitative data in pharmacy research. Research In Social & Administrative Pharmacy. 2022;18(1):2249-53.
72. Mitchinson L, Dowrick A, Buck C, Hoernke K, Martin S, Vanderslott S, et al. Missing the human connection: A rapid appraisal of healthcare workers' perceptions and experiences of providing palliative care during the COVID-19 pandemic. Palliative Medicine. 2021;35(5):852-61.
73. Hoernke K, Djellouli N, Andrews L, Lewis-Jackson S, Manby L, Martin S, et al. Frontline healthcare workers' experiences with personal protective equipment during the COVID-19 pandemic in the UK: a rapid qualitative appraisal. BMJ Open. 2021;11(1):e046199.
74. Kuhlmann E, Brinzac MG, Burau V, Correia T, Ungureanu MI. Health workforce protection and preparedness during the COVID-19 pandemic: a tool for the rapid assessment of EU health systems. European Journal of Public Health. 2021;31(Supplement_4):iv14-iv20.
75. Manby L, Dowrick A, Karia A, Maio L, Buck C, Singleton G, et al. Healthcare workers' perceptions and attitudes towards the UK's COVID-19 vaccination programme: a rapid qualitative appraisal. BMJ Open. 2022;12(2):e051775.
76. Romeu-Labayen M, Tort-Nasarre G, Alvarez B, Subias-Miquel M, Vazquez-Segura E, Marre D, et al. Spanish nurses' experiences with personal protective equipment and perceptions of risk of

For peer review only - http://bmjopen.bmj.com/site/about/guidelines.xhtml

contagion from COVID-19: A qualitative rapid appraisal. Journal of Clinical Nursing. 2022;31(15-16):2154-66.
77. Toth G, Limburg H, Szabo D, Sandor GL, Nagy ZZ, Nemeth J. Rapid assessment of avoidable blindness-based healthcare costs of diabetic retinopathy in Hungary and its projection for the year 2045. British Journal of Ophthalmology. 2021;105(8):1116-20.
78. Kieber-Emmons AM, Miller WL, Rubinstein EB, Howard J, Tsui J, Rankin JL, et al. A Novel Mixed Methods Approach Combining Geospatial Mapping and Qualitative Inquiry to Identify Multilevel Policy Targets: The Focused Rapid Assessment Process (fRAP) Applied to Cancer Survivorship. Journal of Mixed Methods Research. 2022;16(2):183-206.
79. Kobau R, Carry M, Rubenstein BL, Denson D, Uribe C, Zajac J, et al. Implementing the COVID-19 Rapid Community Assessment on Vaccine Confidence: Lessons Learned From Alabama and Georgia. Public Health Reports. 2022;137(5):832-40.
80. Tort-Nasarre G, Alvarez B, Galbany-Estragues P, Subias-Miquel M, Vazquez-Segura E, Marre D, et al. Front-line nurses' responses to organisational changes during the COVID-19 in Spain: A qualitative rapid appraisal. Journal of Nursing Management. 2021;29(7):1983-91.
81. Maaskant JM, Jongerden IP, Bik J, Joosten M, Musters S, Storm-Versloot MN, et al. Strict isolation requires a different approach to the family of hospitalised patients with COVID-19: A rapid qualitative study. International journal of nursing studies. 2021;117:103858.
82. Humphries K, Maxwell S, Bartholomew S, Clutterbuck D. Rapid service evaluation of in-house blood self-sampling for interval STI testing for PrEP cohort at minimal cost. HIV Medicine. 2021;22(SUPPL 2):82-3.
83. Vindrola-Padros C, Ledger J, Hill M, Tomini S, Spencer J, Fulop NJ. The Special Measures for Quality and Challenged Provider Regimes in the English NHS: A Rapid Evaluation of a National Improvement Initiative for Failing Healthcare Organisations. International Journal of Health Policy & Management. 2022;27:27.
84. Gawaya M, Terrill D, Williams E. Using rapid evaluation methods to assess service delivery changes: Lessons learned for evaluation practice during the COVID-19 pandemic. Evaluation Journal of Australasia. 2022;22(1):30-48.
85. Sidhu M, Pollard J, Sussex J. Vertical integration of GP practices with acute hospitals in England and Wales: rapid evaluation.
86. Parkinson S, Smith J, Sidhu M. Early development of primary care networks in the NHS in England: A qualitative mixed-methods evaluation. BMJ Open. 2021;11(12) (no pagination)(e055199).
87. Watson D, Baralle NL, Alagil J, Anil K, Ciccognani S, Dewar-Haggart R, et al. How do we engage people in testing for COVID-19? A rapid qualitative evaluation of a testing programme in schools, GP surgeries and a university. BMC Public Health. 2022;22(1):305.
88. Wanat M, Borek AJ, Pilbeam C, Anthierens S, Tonkin-Crine S. Conducting rapid qualitative interview research during the COVID-19 pandemic-Reflections on methodological choices. Frontiers in Sociology. 2022;7:953872.
89. Crellin NE, Herlitz L, Sidhu MS, Ellins J, Georghiou T, Litchfield I, et al. Examining disparities relating to service reach and patient engagement with COVID-19 remote home monitoring services in England: a mixed methods rapid evaluation. medRxiv. 2022;22.
90. Dupont HB, Kaplan CD, Braam RV, Verbraeck HT, de Vries NK. The application of the rapid assessment and response methodology for cannabis prevention research among youth in the Netherlands. International Journal of Drug Policy. 2015;26(8):731-8.
91. Ong BN. Rapid appraisal and health policy. 1996.
92. Ong BN, Humphris G, Annett H, Rifkin S. Rapid appraisal in an urban setting, an example from the developed world. Soc Sci Med. 1991;32(8):909-15.
93. Organization WH, UNAIDS. SEX-RAR Guide. https://apps.who.int/iris/bitstream/handle/10665/42681/9241545585.pdf?sequence=1&isAllowed=y2002.

For peer review only - http://bmjopen.bmj.com/site/about/guidelines.xhtml

94. Trotter IRT, Needle R, Goosby E, Bates C, Singer M. A Methodological Model for Rapid Assessment, Response, and Evaluation: The RARE Program in Public Health. Field Methods. 2001;13(2):137-59.
95. Valderrama JC, Tortajada S, Alapont L, Vidal A, Perez Ma J, Castellano M, et al. Rapid assessment of drug abuse prevention needs for youth in small Spanish municipalities: Coping with resource limitations. Journal of Drug Issues. 2006;36(1):29-52.
96. Brown DR, Hernandez A, Saint-Jean G, Evans S, Tafari I, Brewster LG, et al. A participatory action research pilot study of urban health disparities using rapid assessment response and evaluation. American Journal of Public Health. 2008;98(1):28-38.
97. Burks DJ, Robbins R, Durtschi JP. American Indian gay, bisexual and two-spirit men: a rapid assessment of HIV/AIDS risk factors, barriers to prevention and culturally-sensitive intervention. Culture, health & sexuality. 2011;13(3):283-98.
98. Eaves ER, Trotter RT, 2nd, Marquez B, Negron K, Doerry E, Mensah D, et al. Possibilities and constraints of rapid online ethnography: Lessons from a rapid assessment of COVID-19 policy for people who use drugs. Frontiers in Sociology. 2022;7:959642.
99. Brown CS, Lloyd S, Murray SA. Using consecutive Rapid Participatory Appraisal studies to assess, facilitate and evaluate health and social change in community settings. BMC Public Health. 2006;6 (no pagination)(68).
100. Lazenbatt A, Lynch U, O'Neill E. Revealing the hidden 'troubles' in Northern Ireland: The role of participatory rapid appraisal. Health Education Research. 2001;16(5):567-78.
101. Annett H. Guidelines for rapid participatory appraisals to assess community health needs: a focus on health improvements for low-income urban and rural areas. Geneva: Division of Strengthening of Health Services World Health Organization available from HMSO; 1995. iv,60 p.
102. Lawlor D. Rapid participatory appraisal of young people's sexual health needs: an evaluation of meta-planning. 1999.
103. Murray SA. Experiences with "rapid appraisal" in primary care: involving the public in assessing health needs, orientating staff, and educating medical students. Bmj. 1999;318(7181):440-4.
104. Sperber NR, Bruening RA, Choate A, Mahanna E, Wang V, Powell BJ, et al. Implementing a Mandated Program Across a Regional Health Care System: A Rapid Qualitative Assessment to Evaluate Early Implementation Strategies. Quality Management in Health Care. 2019;28(3):147-54.
105. Last BS, Buttenheim AM, Futterer AC, Livesey C, Jaeger J, Stewart RE, et al. A pilot study of participatory and rapid implementation approaches to increase depression screening in primary care. BMC Family Practice. 2021;22(1):228.
106. Hanckel B, Ruta D, Scott G, Peacock JL, Green J. The Daily Mile as a public health intervention: a rapid ethnographic assessment of uptake and implementation in South London, UK. BMC Public Health. 2019;19(1):1167.
107. Boyd J, Collins AB, Mayer S, Maher L, Kerr T, McNeil R. Gendered violence and overdose prevention sites: a rapid ethnographic study during an overdose epidemic in Vancouver, Canada. Addiction. 2018;113(12):2261-70.
108. Goepp JG, Meykler S, Mooney NE, Lyon C, Raso R, Julliard K. Provider insights about palliative care barriers and facilitators: results of a rapid ethnographic assessment. Am J Hosp Palliat Care. 2008;25(4):309-14.
109. Green TC, Zaller N, Palacios WR, Bowman SE, Ray M, Heimer R, et al. Law enforcement attitudes toward overdose prevention and response. Drug Alcohol Depend. 2013;133(2):677-84.
110. Quintero Romero S, Bernal R, Barbiero C, Passamonte R, Cattaneo A. A rapid ethnographic study of breastfeeding in the North and South of Italy. Int Breastfeed J. 2006;1:14.
111. Kroeger K, Willilams S, Alexander-Pender C, Ford J, Vasani V. "Just one thing on the plate" - Views of community members and providers on sexual health services in the fort mcpherson area of Southwest Atlanta: Findings from a rapid ethnographic assessment. Sexually Transmitted Infections Conference: STI and AIDS World Congress. 2013;89(SUPPL. 1).

For peer review only

112. Palinkas LA, Mendon SJ, Hamilton AB. Innovations in Mixed Methods Evaluations. Annual Review of Public Health. 2019;40:423-42.
113. Palinkas LA, Zatzick D. Rapid Assessment Procedure Informed Clinical Ethnography (RAPICE) in Pragmatic Clinical Trials of Mental Health Services Implementation: Methods and Applied Case Study. Administration & Policy in Mental Health. 2019;46(2):255-70.
114. Desai A, Donetto S. Evolving ethnographic sensibilities: Using actor-network theory in health services research. BMJ Open. 2019;9 (Supplement 1):A5.
115. Vindrola-Padros C, Vindrola-Padros B. Quick and dirty? A systematic review of the use of rapid ethnographies in healthcare organisation and delivery. BMJ Qual Saf. 2018;27(4):321-30.
116. Ayele R, Manges KA, Leonard C, Lee M, Galenbeck E, Molla M, et al. How Context Influences Hospital Readmissions from Skilled Nursing Facilities: A Rapid Ethnographic Study. Journal of the American Medical Directors Association. 2021;22(6):1248-54.e3.
117. Betsos A, Valleriani J, Boyd J, Bardwell G, Kerr T, McNeil R. "I couldn't live with killing one of my friends or anybody": A rapid ethnographic study of drug sellers' use of drug checking. International Journal of Drug Policy. 2021;87:102845.
118. Collins AB, Boyd J, Mayer S, Fowler A, Kennedy MC, Bluthenthal RN, et al. Policing space in the overdose crisis: A rapid ethnographic study of the impact of law enforcement practices on the effectiveness of overdose prevention sites. International Journal of Drug Policy. 2019;73:199-207.
119. Collins AB, Edwards S, McNeil R, Goldman J, Hallowell BD, Scagos RP, et al. A rapid ethnographic study of risk negotiation during the COVID-19 pandemic among unstably housed people who use drugs in Rhode Island. International Journal of Drug Policy. 2022;103:103626.
120. Manges KA, Ayele R, Leonard C, Lee M, Galenbeck E, Burke RE. Differences in transitional care processes among high-performing and low-performing hospital-SNF pairs: a rapid ethnographic approach. BMJ Quality & Safety. 2021;30(8):648-57.
121. Oh H, Yamada AM. Exploring second generation Korean American alcohol use through church-based participatory research: A rapid ethnographic assessment in Los Angeles, California, United States. Health & Social Care in the Community. 2021;29(5):1359-67.
122. Palinkas LA, Whiteside L, Nehra D, Engstrom A, Taylor M, Moloney K, et al. Rapid ethnographic assessment of the COVID-19 pandemic April 2020 'surge' and its impact on service delivery in an Acute Care Medical Emergency Department and Trauma Center. BMJ Open. 2020;10(10):e041772.
123. Rosteius K, De Boer B, Staudacher S, Verbeek H. Building trusting relationships with staff members of nursing homes during rapid ethnographic research. Frontiers in Sociology. 2022;7:983728.
124. Simpson N, Angland M, Bhogal JK, Bowers RE, Cannell F, Gardner K, et al. 'Good' and 'Bad' deaths during the COVID-19 pandemic: insights from a rapid qualitative study. BMJ Global Health. 2021;6(6):06.
125. Moloney K, Scheuer H, Engstrom A, Schreiber M, Whiteside L, Nehra D, et al. Experiences and Insights from the Early US COVID-19 Epicenter: A Rapid Assessment Procedure Informed Clinical Ethnography Case Series. Psychiatry. 2020;83(2):115-27.
126. Palinkas LA, Engstrom A, Whiteside L, Moloney K, Zatzick D. A Rapid Ethnographic Assessment of the Impact of the COVID-19 Pandemic on Mental Health Services Delivery in an Acute Care Medical Emergency Department and Trauma Center. Administration & Policy in Mental Health. 2022;49(2):157-67.
127. Scott K, Guigayoma J, Palinkas LA, Beaudoin FL, Clark MA, Becker SJ. The measurement-based care to opioid treatment programs project (MBC2OTP): a study protocol using rapid assessment procedure informed clinical ethnography. Addiction Science & Clinical Practice. 2022;17(1):44.
128. Palinkas LA, De Leon J, Salinas E, Chu S, Hunter K, Marshall TM, et al. Impact of the COVID-19 pandemic on child and adolescent mental health policy and practice implementation. International Journal of Environmental Research and Public Health. 2021;18(18) (no pagination)(9622).

For peer review only - http://bmjopen.bmj.com/site/about/guidelines.xhtml

129. Palinkas LA, Springgate BF, Sugarman OK, Hancock J, Wennerstrom A, Haywood C, et al. A Rapid Assessment of Disaster Preparedness Needs and Resources during the COVID-19 Pandemic. International Journal of Environmental Research & Public Health [Electronic Resource]. 2021;18(2):07.
130. Rains E, Stuck A, Ko KJ, Crowley C, Zifferblatt J. Advancing alternative care pathways in geriatric acute unplanned care. Journal of the American Geriatrics Society. 2020;68(SUPPL 1):S130.
131. Taubenberger S, Spencer N, Chang JC, Paul N, Fabre S, Jagessar B, et al. A rapid-cycle assessment strategy for understanding the opioid overdose epidemic in local communities. Substance Abuse. 2021;42(4):888-95.
132. Shrank WH. The Center For Medicare And Medicaid Innovation's blueprint for rapid-cycle evaluation of new care and payment models. Health Affairs (Millwood). 2013;32(4):807-12.
133. Schneeweiss S, Shrank WH, Ruhl M, Maclure M. Decision-making aligned with rapid-cycle evaluation in health care. International Journal of Technology Assessment in Health Care. 2015;31(4):214-22.
134. Farrer K, Melia D, Blackett B, Lloyd H, Beadman C, Donaldson E, et al. Nutritional screening of elderly patients-a health improvement approach to practice. Proceedings of the Nutrition Society. 2011;70 (OCE5):E317.
135. Farrer K, Donaldson E, Blackett B, Lloyd H, Forde C, Melia D, et al. Nutritional screening of elderly patients: a health improvement approach to practice. J Hum Nutr Diet. 2014;27(2):184-91.
136. Dunet DO, Sparling PB, Hersey J, Williams-Piehota P, Hill MD, Hanssen C, et al. A new evaluation tool to obtain practice-based evidence of worksite health promotion programs. Preventing chronic disease. 2008;5(4):A118.
137. Gray A, Baker K. Assessment for change. Acute Medicine. 2017;16 (3):127-8.
138. Kercsmar CM, Beck AF, Sauers-Ford H, Simmons J, Wiener B, Crosby L, et al. Association of an asthma improvement collaborative with health care utilization in medicaid-insured pediatric patients in an urban community. JAMA Pediatrics. 2017;171(11):1072-80.
139. Khalid-Khan S, Braund H, Dare J, Turnridge J, Dalgarno N. Evaluating Child and Adolescent Psychiatry sub-specialty program using rapid-cycle approach. Journal of the Canadian Academy of Child and Adolescent Psychiatry. 2020;29(4):263.
140. Price G, Devaney S, French DP, Holley R, Holm S, Kontopantelis E, et al. Can Real-world Data and Rapid Learning Drive Improvements in Lung Cancer Survival? The RAPID-RT Study. Clinical Oncology (Royal College of Radiologists). 2022;34(6):407-10.
141. Cohn WF, Canan CE, Knight S, Waldman AL, Dillingham R, Ingersoll K, et al. An Implementation Strategy to Expand Mobile Health Use in HIV Care Settings: Rapid Evaluation Study Using the Consolidated Framework for Implementation Research. JMIR MHealth and UHealth. 2021;9(4):e19163.
142. Ohrling M, Ovretveit J, Lockowandt U, Brommels M, Sparring V. Management of the emergency response to the SARS-CoV-2 (COVID-19) outbreak in Stockholm, Sweden, and winter preparations. Journal of Primary Health Care. 2020;12(3):207-14.
143. Williams J, Fairbairn E, McGrath R, Clark A, Healey A, Bakolis I, et al. Development and rapid evaluation of services to support the physical health of people using psychiatric inpatient units during the COVID-19 pandemic: study protocol. Implementation Science Communications. 2021;2(1):12.
144. Joseph C, Goddard N. Influenza vaccine uptake in the elderly: Results from a rapid assessment of the effectiveness of new government policy in England for the winters 2000/2001 and 2001/2002. Vaccine. 2003;21(11-12):1137-48.
145. Demeulemeester M, Lavis N, Balthazar Y, Lechien P, Heijmans S. Rapid safety assessment of a seasonal intradermal trivalent influenza vaccine. Hum Vaccin Immunother. 2017;13(4):889-94.
146. Banga S, Chabanon AL, Eymin C, Caroe T, Butler K, Moureau A. Near real-time safety surveillance of three seasonal influenza vaccines in Europe during influenza season 2017/18. Pharmacoepidemiology and Drug Safety. 2018;27 (Supplement 2):396.

For peer review only - http://bmjopen.bmj.com/site/about/guidelines.xhtml

147. McCarthy A, McMeekin P, Haining S, Bainbridge L, Laing C, Gray J. Rapid evaluation for health and social care innovations: challenges for "quick wins" using interrupted time series. BMC Health Services Research. 2019;19(1):964.
148. Wichmann O, Stocker P, Poggensee G, Altmann D, Walter D, Hellenbrand W, et al. Pandemic influenza A(H1N1) 2009 breakthrough infections and estimates of vaccine effectiveness in Germany 2009-2010. Eurosurveillance. 2010;15(18):1-4.
149. Hamilton AB. Qualitative Methods in Rapid Turn-Around Health Services Research. Spotlight on Women's Health; https://www.hsrd.research.va.gov/for_researchers/cyber_seminars/archives/780-notes.pdf: VA HSR&D Cyberseminar; 2013.
150. Attal JH, Lurie I, Neumark Y. A rapid assessment of migrant careworkers' psychosocial status during Israel's COVID-19 lockdown. Israel Journal of Health Policy Research. 2020;9(1):61.
151. Daviskiba SE, MacKenzie MA, Dow M, Johnston P, Balon R, Javanbakht A, et al. Rapid assessment of mental health of Detroit-area health care workers during the COVID-19 pandemic. Annals of Clinical Psychiatry. 2021;33(2):101-7.
152. Elsawy W, Fouad H, Saeed K. Impact of COVID-19 on mental health and psychosocial support services in the Eastern Mediterranean Region - results of a rapid assessment. Eastern Mediterranean Health Journal. 2022;28(5):321-8.
153. Khubchandani J, Sharma S, Price JH, Wiblishauser MJ, Sharma M, Webb FJ. COVID-19 Vaccination Hesitancy in the United States: A Rapid National Assessment. Journal of Community Health. 2021;46(2):270-7.
154. Petrella AR, Hughes L, Fern LA, Monaghan L, Hannon B, Waters A, et al. Healthcare staff well-being and use of support services during COVID-19: a UK perspective. General Psychiatry. 2021;34(3):e100458.
155. Hammond N, Steels S, King G. Contraceptive and pregnancy concerns in the UK during the first COVID-19 lockdown: A rapid study. Sexual & reproductive healthcare : official journal of the Swedish Association of Midwives. 2022;33:100754.
156. Fisher H, Lambert H, Hickman M, Yardley L, Audrey S. Experiences of the coronavirus disease-19 (COVID-19) pandemic from the perspectives of young people: Rapid qualitative study. Public Health in Practice. 2021;2:100162.
157. Srinivasan M, Asch S, Vilendrer S, Thomas SC, Bajra R, Barman L, et al. Qualitative Assessment of Rapid System Transformation to Primary Care Video Visits at an Academic Medical Center. Annals of Internal Medicine. 2020;173(7):527-35.
158. Keniston A, Sakumoto M, Astik GJ, Auerbach A, Eid SM, Kangelaris KN, et al. Adaptability on Shifting Ground: a Rapid Qualitative Assessment of Multi-institutional Inpatient Surge Planning and Workforce Deployment During the COVID-19 Pandemic. Journal of General Internal Medicine. 2022;22:22.
159. Talati AN, Mallampati D, Johnson JD, West-Honart A, Vladutiu C, Menard MK. 1009 Provider satisfaction with telehealth for maternity care: a rapid assessment of a national survey. American Journal of Obstetrics and Gynecology. 2021;224(2 Supplement):S625.
160. Dunbar MS, Towe VL, Ayer L, Martineau M. Connections to Care (C2C): The Perspectives of Leaders at Community-Based Organizations That Are Integrating Mental Health Supports. Rand health q. 2018;7(4):7.
161. Brown-Johnson C, Lo N, Giannitrapani K, Lowery J, Foglia MB, Walling A, et al. A Rapid Qualitative Synthesis of Insights from the Veterans Administration Life-Sustaining Treatment Decisions Initiative (LSTDI) National Implementation (QI703). Journal of Pain and Symptom Management. 2019;57 (2):457.
162. Hamarman A, Mason P, Kroeger K, Alexander-Pender C. The impact of budget cuts among local STD programs in New Jersey: Results of a rapid ethnographic assessment. Sexually Transmitted Diseases. 2014;1):S93.

For peer review only - http://bmjopen.bmj.com/site/about/guidelines.xhtml

163. Loosier PS, Carter MW, Hsu KK, Doshi S, Peterson Maddox BL, Kroeger K, et al. Provision of STD Services in Community Settings After the Loss and Return of State Funding to Support Service Provision: Observations From Select Providers in Massachusetts, 2010 and 2013. Journal of public health management and practice : JPHMP. 2020;26(1):E18-E27.
164. Delaney AE, Dadlez NM, Marshall AC. Alternative approach to pediatric cardiac quality assessment for low-volume centers. Congenital Heart Disease. 2019;14(4):665-70.
165. Rodriguez RM, Torres JR, Chang AM, Haggins AN, Eucker SA, O'Laughlin KN, et al. The Rapid Evaluation of COVID-19 Vaccination in Emergency Departments for Underserved Patients Study. Annals of Emergency Medicine. 2021;78(4):502-10.
166. Cakir B, Uner S, Temel F, Akin L. Lot quality survey: An appealing method for rapid evaluation of vaccine coverage in developing countries - Experience in Turkey. BMC Public Health. 2008;8 (no pagination)(240).
167. Hasford J. Large Simple Double-Blind Randomized Trials for the Rapid Assessment of the Effectiveness of COVID-19 Vaccines. Journal of Infectious Diseases. 2020;222(9):1571-2.
168. Gray TA, Dumville JC, Christie J, Cullum NA. Rapid research and implementation priority setting for wound care uncertainties. PLoS ONE. 2017;12(12):e0188958.
169. Newlands RS, Power A, Young L, Watson M. Quality improvement of community pharmacy services: a prioritisation exercise. International Journal of Pharmacy Practice. 2018;26(1):39-48.
170. Cowan K, Fulop NJ, Harshfield A, Ng PL, Ntouva A, Sidhu M, et al. Rapid prioritisation of topics for rapid evaluation: the case of innovations in adult social care and social work. Health Research Policy & Systems. 2021;19(1):34.
171. Dudovitz RN, Russ S, Berghaus M, Iruka IU, DiBari J, Foney DM, et al. COVID-19 and Children's Well-Being: A Rapid Research Agenda. Maternal & Child Health Journal. 2021;25(11):1655-69.
172. Khan MA, Barratt MS, Krugman SD, Serwint JR, Dumont-Driscoll M, Investigators C. Variability of the institutional review board process within a national research network. Clin Pediatr (Phila). 2014;53(6):556-60.
173. Bower P, Howells K, McCorkindale S, Bridges L, Sidaway M. Rapid recruitment of large cohort to support trials in general practice: The role of FARSITE. Trials Conference: 3rd International Clinical Trials Methodology Conference United Kingdom. 2015;16(Supplement 2).
174. Brown JSL, Murphy C, Kelly J, Goldsmith K. How can we successfully recruit depressed people? Lessons learned in recruiting depressed participants to a multi-site trial of a brief depression intervention (the 'CLASSIC' trial). Trials. 2019;20(1):131.
175. Deane AM, Hodgson CL, Young P, Little L, Singh V, Poole A, et al. The rapid and accurate categorisation of critically ill patients (RACE) to identify outcomes of interest for longitudinal studies: a feasibility study. Anaesthesia and intensive care. 2017;45(4):476-84.
176. Whelan AM. Consultation with non-English speaking communities: rapid bilingual appraisal. 2004.
177. Alolod G, Siminoff LA, Traino HM. Usability of the rapid assessment of hospital procurement barriers in donation (RAPID) as a tool for OPO hospital development staff. Transplantation. 2015;2):S47.
178. Alolod GP, Traino HM, Siminoff LA. Utility and Usability of the Rapid Assessment of Hospital Procurement Barriers in Donation (RAPiD) as a Tool for OPO Hospital Development Staff. Prog Transplant. 2016;26(3):241-8.
179. Traino HM, Alolod GP, Shafer T, Siminoff LA. Interim results of a national test of the rapid assessment of hospital procurement barriers in donation (RAPiD). American Journal of Transplantation. 2012;12(11):3094-103.
180. Corley SS, Gillezeau C, Molina L, Alpert N, Eugene A, Lieberman-Cribbin W, et al. Using Rapid Research Implementation and Collaborations to Assess the Mental Health Impact of the COVID-19 Pandemic Among Community and Clinical Cohorts. Disaster Medicine & Public Health Preparedness. 2021:1-5.

For peer review only - http://bmjopen.bmj.com/site/about/guidelines.xhtml

181. Xiang JJ, Roy A, Summers C, Delvy M, O'Donovan JL, Christensen J, et al. Improving hematology/oncology clinical research recruitment via universal prescreening: The VA Connecticut (VACT) Cancer Center experience. Journal of Clinical Oncology Conference. 2021;39(28 SUPPL).
182. Wesolowski A, Gibson D, Agarwal S, Lambrou A, Kirk G, Labrique A, et al. Rapid assessments of non-pharmaceutical intervention uptake and population mobility patterns elucidate SARS-CoV-2 transmission dynamics. Open Forum Infectious Diseases. 2020;7(SUPPL 1):S848.
183. Cake C, Ogburn E, Pinches H, Coleman G, Seymour D, Woodard F, et al. Development and evaluation of rapid data-enabled access to routine clinical information to enhance early recruitment to the national clinical platform trial of COVID-19 community treatments. Trials [Electronic Resource]. 2022;23(1):62.
184. Moodie Z, Janes H, Huang Y. New clinical trial designs for HIV vaccine evaluation. Curr Opin HIV AIDS. 2013;8(5):437-42.
185. Hensel JM, Shaw J, Jeffs L, Ivers NM, Desveaux L, Cohen A, et al. A pragmatic randomized control trial and realist evaluation on the implementation and effectiveness of an internet application to support self-management among individuals seeking specialized mental health care: A study protocol. BMC Psychiatry. 2016;16 (1) (no pagination)(350).
186. Douglas JE, Liu S, Ma J, Wolff RA, Pant S, Maitra A, et al. PIONEER-Panc: a platform trial for phase II randomized investigations of new and emerging therapies for localized pancreatic cancer. BMC Cancer. 2022;22(1):14.
187. Griffiths GO, FitzGerald R, Jaki T, Corkhill A, Reynolds H, Ewings S, et al. AGILE: a seamless phase I/IIa platform for the rapid evaluation of candidates for COVID-19 treatment: an update to the structured summary of a study protocol for a randomised platform trial letter. Trials [Electronic Resource]. 2021;22(1):487.
188. Murray DD, Babiker AG, Baker JV, Barkauskas CE, Brown SM, Chang CC, et al. Design and implementation of an international, multi-arm, multi-stage platform master protocol for trials of novel SARS-CoV-2 antiviral agents: Therapeutics for Inpatients with COVID-19 (TICO/ACTIV-3). Clinical Trials. 2022;19(1):52-61.
189. Short NJ, Borthakur G, Pemmaraju N, Dinardo CD, Kadia TM, Jabbour E, et al. A multi-arm phase Ib/II study designed for rapid, parallel evaluation of novel immunotherapy combinations in relapsed/refractory acute myeloid leukemia. Leukemia & Lymphoma. 2022:1-10.
190. RECOVERY. Randomised evaluation of Covid-19 therapy (RECOVERY) https://www.recoverytrial.net/files/recovery-protocol-v25-0-2022-05-23.pdf2020 [
191. RECOVERY. Dexamethasone in Hospitalized Patients with Covid-19. New England Journal of Medicine. 2021;384(8):693-704.
192. Simpson CR, Lone N, McMenamin J, Gunson R, Robertson C, Ritchie LD, et al. Early estimation of pandemic influenza Antiviral and Vaccine Effectiveness (EAVE): use of a unique community and laboratory national data-linked cohort study. Health Technol Assess. 2015;19(79):1-32.
193. Glasgow RE, Kessler RS, Ory MG, Roby D, Gorin SS, Krist A. Conducting rapid, relevant research: Lessons learned from the my own health report project. American Journal of Preventive Medicine. 2014;47(2):212-9.
194. Taylor B, Henshall C, Kenyon S, Litchfield I, Greenfield S. Can rapid approaches to qualitative analysis deliver timely, valid findings to clinical leaders? A mixed methods study comparing rapid and thematic analysis. BMJ Open. 2018;8 (10) (no pagination)(e019993).
195. Burgess-Allen J, Owen-Smith V. Using mind mapping techniques for rapid qualitative data analysis in public participation processes. Health Expect. 2010;13(4):406-15.
196. Nevedal AL, Reardon CM, Opra Widerquist MA, Jackson GL, Cutrona SL, White BS, et al. Rapid versus traditional qualitative analysis using the Consolidated Framework for Implementation Research (CFIR). Implementation Science. 2021;16(1):67.

For peer review only - http://bmjopen.bmj.com/site/about/guidelines.xhtml

197. Gale RC, Wu J, Erhardt T, Bounthavong M, Reardon CM, Damschroder LJ, et al. Comparison of rapid vs in-depth qualitative analytic methods from a process evaluation of academic detailing in the Veterans Health Administration. Implementation Science. 2019;14(1):11.
198. Li Y, Li M, Rice M, Su Y, Yang C. Phased Implementation of COVID-19 Vaccination: Rapid Assessment of Policy Adoption, Reach and Effectiveness to Protect the Most Vulnerable in the US. International Journal of Environmental Research & Public Health [Electronic Resource]. 2021;18(14):19.
199. Tan RKJ, Lim JM, Lo JJ, Teo AKJ, O'Hara CA, Ching AH, et al. Conducting rapid qualitative research to support sex workers' health and social needs in the face of COVID-19: capitalising on stakeholder networks from the HIV response in Singapore to drive policymaking. Sexually Transmitted Infections. 2021;97(2):84.
200. Lewinski AA, Crowley MJ, Miller C, Bosworth HB, Jackson GL, Steinhauser K, et al. Applied Rapid Qualitative Analysis to Develop a Contextually Appropriate Intervention and Increase the Likelihood of Uptake. Medical Care. 2021;59(Suppl 3):S242-S51.
201. Rolf F, Campbell N, Thompson S, Argus G. Australians' Experience of the COVID-19 Pandemic: Advantages and Challenges of Scaling Up Qualitative Research Using Large-Scale Rapid Analysis and Building Research Capacity Across Rural Australia. International Journal of Qualitative Methods. 2021;20:16094069211051937.
202. Vazquez E, Chobdee J, Nasrollahzadeh N, Cheney A. Personal Freedom and Social Responsibility in Slowing the Spread of COVID-19: A Rapid Qualitative Study. Health Education & Behavior. 2022;49(1):26-34.
203. Elwy AR, Clayman ML, LoBrutto L, Miano D, Ann Petrakis B, Javier S, et al. Vaccine hesitancy as an opportunity for engagement: A rapid qualitative study of patients and employees in the U.S. Veterans Affairs healthcare system. Vaccine: X. 2021;9:100116.
204. Begg NT, Gill ON, White JM. COVER (Cover Of Vaccination Evaluated Rapidly): Description of the England and Wales scheme. Public Health. 1989;103(2):81-9.
205. Currow DC, Vella-Brincat J, Fazekas B, Clark K, Doogue M, Rowett D. Pharmacovigilance in hospice/palliative care: Rapid report of net clinical effect of metoclopramide. Journal of Palliative Medicine. 2012;15(10):1071-5.
206. Shimkhada R, Attai D, Scheitler AJ, Babey S, Glenn B, Ponce N. Using a Twitter Chat to Rapidly Identify Barriers and Policy Solutions for Metastatic Breast Cancer Care: Qualitative Study. JMIR Public Health and Surveillance. 2021;7(1):e23178.
207. Noack PS, Moore JA, Poon M. Advanced Imaging Reduces Cost Compared to Standard of Care in Emergency Department of Triage of Acute Chest Pain. Health Services Research. 2018;53(4):2384-405.
208. Centers for Disease C, Prevention. Rapid assessment of influenza vaccination coverage among HMO members--northern California influenza seasons, 2001-02 through 2004-05. MMWR Morb Mortal Wkly Rep. 2005;54(27):676-8.
209. Gagne JJ, Wang S, Schneeweiss S. A multivariable-adjusted rapid assessment of the association between zoledronic acid and myocardial infarction. Pharmacoepidemiology and Drug Safety. 2013;1):378.
210. Dodd CN, de Ridder M, Huang WT, Weibel D, Giner-Soriano M, Perez-Vilar S, et al. Incidence rates of narcolepsy diagnoses in Taiwan, Canada, and Europe: The use of statistical simulation to evaluate methods for the rapid assessment of potential safety issues on a population level in the SOMNIA study. PLoS ONE. 2018;13(10):e0204799.
211. Alguacil-Ramos AM, Portero-Alonso A, Pastor-Villalba E, Muelas-Tirado J, Diez-Domingo J, Sanchis-Ferrer A, et al. Rapid assessment of enhanced safety surveillance for influenza vaccine. Public Health. 2019;168:137-41.
212. Carrillo GA, Cohen-Wolkowiez M, D'Agostino EM, Marsolo K, Wruck LM, Johnson L, et al. Standardizing, harmonizing, and protecting data collection to broaden the impact of COVID-19

For peer review only - http://bmjopen.bmj.com/site/about/guidelines.xhtml

research: the rapid acceleration of diagnostics-underserved populations (RADx-UP) initiative. Journal of the American Medical Informatics Association. 2022;29(9):1480-8.
213. Dambha-Miller H, Griffin SJ, Young D, Watkinson P, Tan PS, Clift AK, et al. The Use of Primary Care Big Data in Understanding the Pharmacoepidemiology of COVID-19: A Consensus Statement From the COVID-19 Primary Care Database Consortium. Annals of Family Medicine. 2021;19(2):135-40.
214. Harrison PW, Lopez R, Rahman N, Allen SG, Aslam R, Buso N, et al. The COVID-19 Data Portal: accelerating SARS-CoV-2 and COVID-19 research through rapid open access data sharing. Nucleic Acids Research. 2021;49(W1):W619-W23.
215. Talwai A, Wing V, Itzkovich Y, Galaznik A, Chatterjee A, Jain R, et al. PIN83 The COVID-19 Research Database: Building One of the Largest PRO Bono Real-World DATA Repositories. Value in Health. 2021;24(Supplement 1):S121.
216. Saunders CL, Gkousis E. Impact of telephone triage on access to primary care for people living with multiple long-term health conditions: rapid evaluation. Health and Social Care Delivery Research. 2022;10(18).
217. Agaku IT, Nkosi L, Agaku QD, Gwar J, Tsafa T. A Rapid Evaluation of the US Federal Tobacco 21 (T21) Law and Lessons From Statewide T21 Policies: Findings From Population-Level Surveys. Preventing Chronic Disease. 2022;19:E29.
218. Lester C. Priority setting with health inequality impact assessment. 2001.
219. Walton H, Vindrola-Padros C, Crellin NE, Sidhu MS, Herlitz L, Litchfield I, et al. Patients' experiences of, and engagement with, remote home monitoring services for COVID-19 patients: A rapid mixed-methods study. Health Expectations.n/a(n/a).
220. Shaw J, Feeney H, Meunier-Sham J, Hazard K, Plante P, Petricone R. An evaluation of a rapid conversion to teleSANE in response to COVID-19. American Journal of Community Psychology. 2022;28:28.
221. Hawley S, Ali MS, Berencsi K, Judge A, Prieto-Alhambra D. Sample size and power considerations for ordinary least squares interrupted time series analysis: a simulation study. . Clinical Epidemiology. 2019;11:197-205.
222. Ramsay CR, Matowe L, Grilli R, Grimshaw JM, Thomas RE. Interrupted time series designs in health technology assessment: lessons from two systematic reviews of behavior change strategies. . International Journal of Technology Assessment in Health Care. 2003;19(4):613-23.
223. Hategeka C, Ruton H, Karamouzian M, Lynd L, Law MR. Use of interrupted time series methods in the evaluation of health system quality improvement interventions: a methodological systematic review. BMJ Global Health. 2020;5:e003567.
224. EPOC. EPOC Resources for Review Authors https://epoc.cochrane.org/resources/epoc-resources-review-authors: Cochrane Collaboration; 2017 [
225. Campbell D, Edwards B, Milat A, Thackway S, Whittaker E, Goudswaard L, et al. NSW Health COVID-19 Emergency Response Priority Research program: a case study of rapid translation of research into health decision making. Public Health Research & Practice. 2021;31(4):10.
226. Murphy GT, Sampalli T, Embrett M, Sim M, Murdoch J, McIsaac K, et al. The Network of Scholars Strategy: A Case Study of Embedded Research Activities in Nova Scotia to Advance Health System Impact and Outcomes. Healthcarepapers. 2022;20(3):33-43.
227. Hanney SR, Straus SE, Holmes BJ. Saving millions of lives but some resources squandered: emerging lessons from health research system pandemic achievements and challenges. Health Research Policy & Systems. 2022;20(1):99.
228. Henderson LR, McShane H, Kiparoglou V. Rapid research response to the COVID-19 pandemic: perspectives from a National Institute for Health Biomedical Research Centre. Health Research Policy & Systems. 2022;20(1):24.
229. Vindrola-Padros C. Can We Re-Imagine Research So It Is Timely, Relevant and Responsive? Comment on "Experience of Health Leadership in Partnering with University-Based Researchers in

For peer review only - http://bmjopen.bmj.com/site/about/guidelines.xhtml

Canada: A Call to 'Re-Imagine' Research". International Journal of Health Policy & Management. 2021;10(3):172-5.
230. Akande TM, Eshetu M, Bonsu G. Rapid assessment as an evaluation tool for Polio National Immunisation Days in Brong Ahafo Region, Ghana. Annals of African Medicine. 2005;4(4):172-6.
231. Kengne-Ouafo JA, Nji TM, Tantoh WF, Nyoh DN, Tendongfor N, Enyong PA, et al. Perceptions of consent, permission structures and approaches to the community: a rapid ethical assessment performed in North West Cameroon. BMC Public Health. 2014;14:1026.
232. Abay S, Addissie A, Davey G, Farsides B, Addissie T. Rapid Ethical Assessment on Informed Consent Content and Procedure in Hintalo-Wajirat, Northern Ethiopia: A Qualitative Study. PLoS ONE. 2016;11(6):e0157056.
233. Addissie A, Abay S, Feleke Y, Newport M, Farsides B, Davey G. Cluster randomized trial assessing the effects of rapid ethical assessment on informed consent comprehension in a low-resource setting. BMC medical ethics. 2016;17(1):40.
234. Gebresilase TT, Andargie Z, Tsegay G, Aseffa A, Davey G, Newport M, et al. Using rapid ethical appraisal (REA) to design a contextualized consent process for biomedical studies the Ethiopia. American Journal of Tropical Medicine and Hygiene. 2015;93 (4 Supplement):23.
235. Bull S, Farsides B, Ayele FT. Tailoring information provision and consent processes to research contexts: The value of rapid assessments. Journal of Empirical Research on Human Research Ethics. 2012;7(1):37-52.
236. Feeley N, Cossette S, Côté J, Héon M, Stremler R, Martorella G, et al. The importance of piloting an RCT intervention. Canadian Journal of Nursing Research. 2009;41(2):85-99.
237. Jones A, Pierce M, Sutton M, Mason T, Millar T. Does paying service providers by results improve recovery outcomes for drug misusers in treatment in England? Addiction. 2018;113(2):279-86.
238. Mason T, Sutton M, Whittaker W, McSweeney T, Millar T, Donmall M, et al. The impact of paying treatment providers for outcomes: difference-in-differences analysis of the 'payment by results for drugs recovery' pilot. Addiction. 2015;110(7):1120-8.
239. Mason T, Whittaker W, Jones A, Sutton M. Did paying drugs misuse treatment providers for outcomes lead to unintended consequences for hospital admissions? Difference-in-differences analysis of a pay-for-performance scheme in England. Addiction. 2021;116(11):3082-93.
240. BRACE. The trade off between rigour and real world evidence needs https://www.birmingham.ac.uk/research/brace/blogs/.aspx: University of Birmingham; 2021 [
241. Raj N, Fernandes S, Charyulu N, Dubey A, Ravi GS, Hebbar S. Postmarket surveillance: a review on key aspects and measures on the effective functioning in the context of the United Kingdom and Canada. Therapeutic Advances in Drug Safety. 2019;10:2042098619865413.
242. NICE, Excellence NIfHaC. Evidence standards framework for digital health technologies https://www.nice.org.uk/about/what-we-do/our-programmes/evidence-standards-framework-for-digital-health-technologies: National Institute for Health and Care Excellence; 2021 [
243. Reyes M. Research in the Time of COVID-19: Challenges of Research Ethics Committees. Journal of the Asean Federation of Endocrine Societies. 2020;35(1):29-32.
244. Tamariz L, Hendler FJ, Wells JM, Anderson A, Bartlett S. A Call for Better, Not Faster, Research Ethics Committee Reviews in the Covid-19 Era. Ethics & Human Research. 2021;43(5):42-4.
245. Murphy E, Tierney E, Ni She E, Killilea M, Donaghey C, Daly A, et al. COVID-19: Public and patient involvement, now more than ever. Hrb Open Research. 2020;3:35.

For peer review only - http://bmjopen.bmj.com/site/about/guidelines.xhtml

Figure 1: PRISMA diagram showing study flow for the review

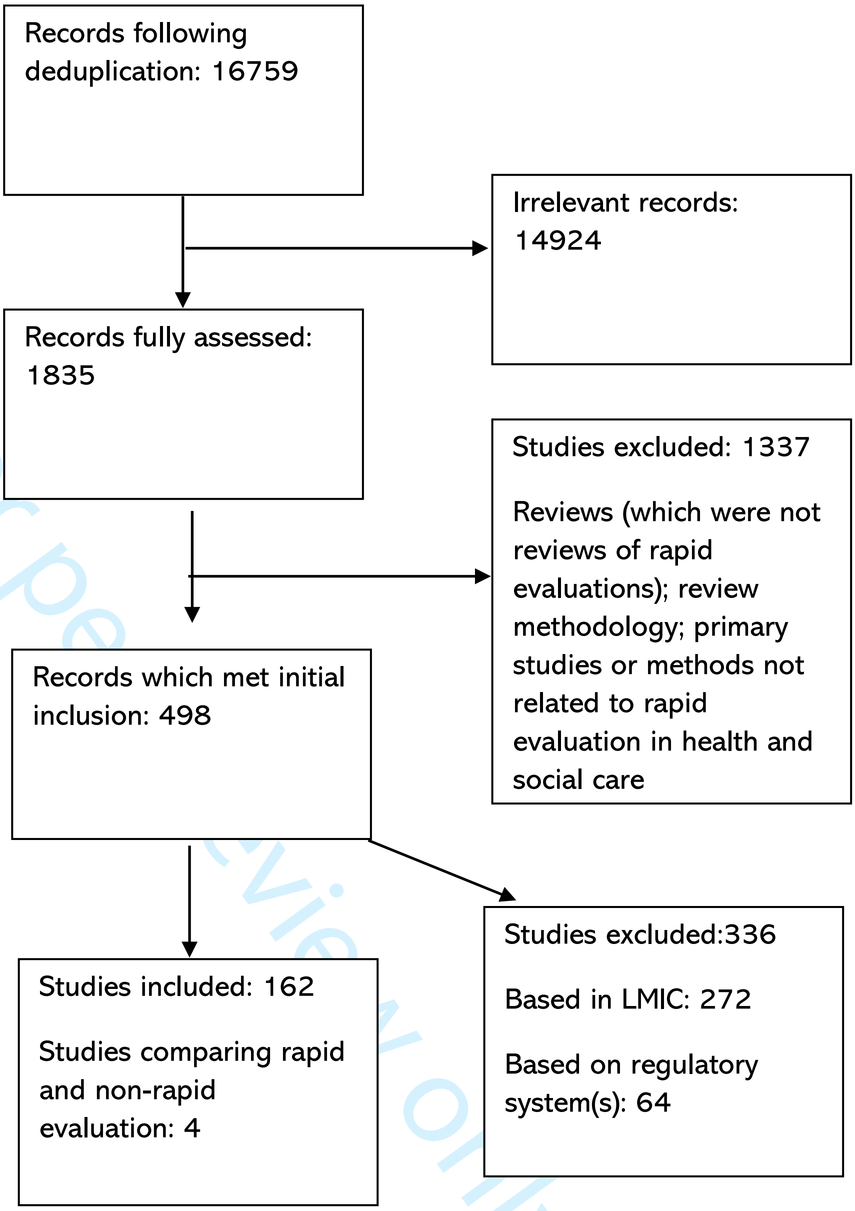

Appendix: Medline Search for rapid evaluations

Rapid evaluations

Database: Ovid MEDLINE(R) and Epub Ahead of Print, In-Process & Other Non-Indexed Citations and Daily <1946 to September 13, 2022>
Search Strategy:
----
1 rapid evaluation.tw. (1469)
2 (Rapid adj2 (assessment* or report* or qualitative research or research or studies or study or evaluation* or
appraisal* or approach* or response brief* or response program*)).ti. (2911)
3 ((Fast or faster) adj (assessments or reports or qualitative research or research or studies or evaluations or
appraisals or approaches or response briefs or response program*)).tw. (87)
4 ((limit* or short* or tight or efficient or pragmatic) adj2 (deadline* or time or timescale or timeline or time
period or time frame) adj2 (assessments or reports or qualitative research or research or studies or evaluations or
appraisals or approaches or response briefs or response program*)).tw. (275)
5 ((Speed or speedy) adj (assessments or reports or qualitative research or research or studies or evaluations or
appraisals or approaches or response briefs or response program*)).tw. (88)
6 (Abbreviated adj (assessments or reports or qualitative research or research or studies or evaluations or
appraisals or approaches or response briefs or response program*)).tw. (11)
7 (Accelerated adj (assessments or reports or qualitative research or research or studies or evaluations or
appraisals or approaches or response briefs or response program*)).tw. (152)
8 (Brief adj (assessments or reports or qualitative research or research or studies or evaluations or appraisals or
approaches or response program*)).tw. (494)
9 (constrained by time adj10 (assessments or reports or qualitative research or research or studies or evaluations
or appraisals or approaches or response briefs or response program*)).tw. (3)
10 (Expedited adj (assessments or reports or qualitative research or research or studies or evaluations or
appraisals or approaches or response briefs or response program*)).tw. (14)
11 (Express adj (assessments or reports or qualitative research or research or studies or evaluations or appraisals
or approaches or response briefs or response program*)).tw. (7)
12 (Immediate adj (qualitative research or research or studies or evaluations or appraisals or approaches or

For peer review only

response briefs or response program*)).tw. (61)
13 ((Quick adj2 dirty) and (assessments or reports or qualitative research or research or studies or evaluations or appraisals or approaches or response briefs or response program*)).tw. (16)
14 (Quick* adj (assessments or reports or qualitative research or research or studies or evaluations or appraisals or approaches or response briefs or response program*)).tw. (71)
15 (Rapid adj (assessments or reports or qualitative research or research or studies or evaluations or appraisals or approaches or response brief* or response program*)).tw. (292)
16 (Stream-line* adj (assessments or reports or qualitative research or research or studies or evaluations or appraisals or approaches or response briefs or response program*)).tw. (1)
17 (Streamline* adj (assessments or reports or qualitative research or research or studies or evaluations or appraisals or approaches or response briefs or response program*)).tw. (59)
18 (timeliness adj6 (assessments or reports or qualitative research or research or studies or evaluations or appraisals or approaches or response briefs or response program*)).tw. (211)
19 (Timely adj (assessments or reports or qualitative research or research or studies or evaluations or appraisals or approaches or response briefs or response program*)).tw. (146)
20 or/1-19 (5967)
21 ((Fast or faster) adj (appraisal or approach or study or assessment or report)).tw. (577)
22 ((limit* or short* or tight or efficient or pragmatic) adj2 (deadline* or time or timescale or timeline or time period or time frame) adj2 (appraisal or approach or study or assessment or report)).tw. (650)
23 ((Speed or speedy) adj (appraisal or approach or study or assessment or report)).tw. (115)
24 (Abbreviated adj (appraisal or approach or study or assessment or report)).tw. (45)
25 (Accelerated adj (appraisal or approach or study or assessment or report)).tw. (79)
26 (Brief adj (appraisal or approach or study or assessment or report)).tw. (6166)
27 (constrained by time adj10 (appraisal or approach or study or assessment or report)).tw. (3)
28 (Expedited adj (appraisal or approach or study or assessment or report)).tw. (21)
29 (Express adj (appraisal or approach or study or assessment or report)).tw. (56)
30 (Immediate adj (appraisal or approach or study or assessment or report)).tw. (376)
31 ((Quick adj2 dirty) and (appraisal or approach or study or assessment or report)).tw. (23)

For peer review only

32 (Quick* adj (appraisal or approach or study or assessment or report)).tw. (433)
33 (Rapid adj (appraisal or approach or study or assessment or report)).tw. (4268)
34 (Stream-line* adj (appraisal or approach or study or assessment or report)).tw. (3)
35 (Streamline* adj (appraisal or approach or study or assessment or report)).tw. (234)
36 (timeliness adj6 (appraisal or approach or study or assessment or report)).tw. (277)
37 (Timely adj (appraisal or approach or study or assessment or report)).tw. (329)
38 or/21-37 (13624)
39 evidence-based practice/ (9666)
40 evidence-based medicine/ (72011)
41 Decision Making/ (92841)
42 exp Research/mt, st, sn, td [Methods, Standards, Statistics & Numerical Data, Trends] (84281)
43 exp Animals/ not (exp Animals/ and Humans/) (4671550)
44 39 or 40 or 41 or 42 (250808)
45 38 and 44 (214)
46 20 or 45 (6162)
47 46 not 43 (5569)
48 47 and 20*.ed. (3932)

Rapid reviews (Not included in update September 2022 search)

Database: Ovid MEDLINE(R) and Epub Ahead of Print, In-Process & Other Non-Indexed Citations and Daily <1946 to February 24, 2020>
Search Strategy:
----
1 ((abbreviated adj review?) or (abbreviated adj synthes?s) or (accelerated adj2 review?) or (accelerated adj2 synthes?s) or (brief adj synthes?s) or (expedited adj2 review?) or (expedited adj2 synthes?s) or (meta adj method$) or (meta adj evaluat$) or (rapid adj2 review?) or (rapid adj2 assess$) or "rapid health technology assess$" or (rapid adj HTA?) or (rapid adj approach$) or (rapid adj search$) or (realis$ adj approach$) or (realis$ adj evaluat$) or (realis$ adj synthes?s) or (speed$ adj2 review?) or (streamline$ adj2 review?) or (streamline$ adj2 synthes?s) or (stream-line$ adj2 review?) or (stream-line$ adj2 synthes?s) or (fast$ adj2 review?) or (fast$ adj2 synthes?s)).tw. (9897)
2 (rapid adj2 synthes?s).tw. not (ch or cs).fs. (1152)
3 1 or 2 (11015)
4 exp Animals/ not (exp Animals/ and Humans/) (4671550)

For peer review only

5 3 not 4 (9598)

6 5 and ("2014$" or "2015$" or "2016$" or "2017$" or "2018$" or "2019$" or "2020$").ed. (3345)

For peer review only

Appendix 2: Further information on identified studies

Supplementary Table 1: Descriptions for methodologies described as specifically designed to enable rapid evaluation

Name of methodology	Description of methods	Example(s)
Rapid assessment/appraisal procedure/process (RAP) [multiple terms used for variations and adaptations of core methods.]	Exemplifies use of triangulation of multiple data sources (e.g. interviews and surveys with or without additional data sources). Multiple adaptations identified. Use focused on provision and delivery, barriers, facilitators and perceptions of benefit.	Fergus 2010 (1)(adaptation) Kieber-Emmons 2018 (adapted) (2); Wright 2015; (3) Manderson 1992;(4) McMullen 2011(5) (for clinical informatics); Needle 2000/3(6, 7) Adapted for use in evaluation of changes. Holdsworth(8) Used for policy formulation. Peiro(9, 10) Fitch 2000; 2004 Stimson 2006(11-13)Walton 2021(14); Humphries 2021(15); Vindrola-Padros 2022(16) Gawaya 2022(17); Sidhu 2021 (18); Parkinson 2021(19); Watson 2022(20) Limitations(21, 22)
Rapid appraisal [term used for variations and adaptations of core methods]	Interviews with range of individuals, documentary evidence, focused ethnographic visits, survey; thematic analysis. Not all methods used in all iterations. Related to methods of rapid assessment and response and rapid assessment, response and evaluation.(23-27) Use focused on views or assessments of policies or services.	Green 2015(28) (refs Beebe 2012) (29) Hamilton 1993(30) Rowa-dewar 2008 (31) Harvey 2001(26, 27) Murphy 2018(32)
Rapid evaluation approach of an emergent program	Semi-structured interviews with multiple types of stakeholders; triangulation of data from different stakeholders using same methods; thematic analysis. Focus on service user experiences.	Foreman-Mackey 2019(33, 34) (refs Beebe 2012 (29))

Rapid participatory appraisal	Adaptation of rapid appraisal to involve community in design of appraisal study; includes use of consecutive studies in same community. Use focused on access (and barriers to access) to services; perspectives, experiences and satisfaction relating to services.	Brown 2006 (35) Lazenblatt 2001 (36) Murray 1994, 1999(24, 25), Annett 1995 (guidance) (37)
Rapid participatory appraisal with meta-planning	Uses peer facilitation of data collection; triangulates two separate groups of participant responses.	Lawlor 1999 (38)
Rapid ethnographic assessment/appraisal /Rapid ethnography	Assessment of intervention in naturalistic setting. Interviews, focus groups, observations of implementation, analysis of routine data sources (not all methods in all studies). Typically used interview and survey data, triangulated. Includes but is not limited to evaluation of services; focus on views and experiences. Question mark over whether truly ethnographic. (39, 40)	Hanckel 2019(41-44) Boyd 2018 (42) Goepp 2008,(43) Green 2013(44) Ackerman 2017(45) Quintero-Romero;(46) Kroeger 2013(47) Ayele 2021; Manges 2021(48, 49) Betsos 2021(50); Collins 2019;(51) Collins 2022(52); Oh 2021(53)
Rapid assessment and response (RAR)	Interviews with representative individuals/ groups (also described as focus groups), integrates findings with data from existing records and desk research; researcher training required for effectiveness. Focus on knowledge and service capacity and development	Dupont 2015;(54) Stimson 2006(13) Ong 1996, 1991(55, 56) WHO 2002 (guidance for psychoactive drugs and sexual risk behaviours) (57)
Rapid assessment, response and evaluation (RARE)	A model for using RAR within context of public health policy development; (58) RAR in implementation evaluation. Substantive training component for proper implementation. Rapid qualitative ethnographic methods: use of existing data; direct involvement of community leaders/health providers; explicit evaluation component. Focus on care coverage, access and barriers.	Trotter 2001(58) Needle 2000,2003(6, 7) Brown 2008(59)Burks 2011(60) Valderrama 2006 (61)(combined with policy level surveying)

For peer review only

Rapid cycle evaluation; Rapid feedback evaluation	Use of consolidated framework for implementation research (CFIR) using observational field notes and semi-structured interviews across multiple sites to enable rapid cycle data analysis and generate actionable findings during the research project life cycle. Using Expert recommendations for implementing change (ERIC) (34, 62) Identifies barriers and facilitators to components of initiatives.	Zakocs 2015; Keith 2017; Skillman 2018; Shrank 2013; Cohn 2021; Ohrling 20202; Last 2021: Williams; 2021 (63-69)
Plan-do-study-act cycles	Method drawn from quality improvement.<br>Small-scale implementation and evaluation of changes, with weekly or monthly (e.g.) tracking of routinely collected data at ward/hospital/district level. Shares some features with studies using real-time feedback in other designs.(70)	Farrer 2014; Kercsmar 2017 (71, 72)
Rapid cycle evaluation building in plan do study act (RAPID)	Method to support decision making based on rapid cycle evaluation of electronic data	Schneeweiss 2015(73)
Rapid assessment procedure informed clinical ethnography (RAPICE)	Mixed methods approach using a combination of rapid appraisal and ethnography to understand implementation processes. Linked to a pragmatic trial; focused on barriers and facilitators.	Palinkas 2019a/b; Palinkas 2022; Moloney 2020 (74-77)
Focused rapid assessment process	Place-focused adaptation of rapid assessment process; combining geospatial mapping and qualitative assessment.	Kieber-Emmons 2022(78)
Qualitative rapid assessment	Focus groups and semi-structured interviews; thematic analysis; focus on acceptability and feasibility.	Guise 2018(79)
Qualitative rapid appraisal, Rigorous analysis (Q-RARA)	Qualitative mixed methods: site visit, semi-structured interviews, observations, photographs, floor plans, and social scanning data; integrated qualitative analysis approach. Focus on organisational function and processes in small primary health care organisations.	Phillips 2014(80)

For peer review only

Rapid gap assessment approach	Mixed methods involving targeted site identification; checklist-based data collection; notes from participating centres and open ended questions; use of online search methods to identify sites. Approach described as modified rapid gap assessment.	Williams 2019(81)
Swift Worksite Assessment and Translation (SWAT)	Multiple evaluation approaches – site visits, interviews etc. Includes disseminations stage; aims to assess effectiveness of workplace health policies may be suitable for innovations aimed at HCP	Dunet 2008(82)
Pilot implementation	Although we did not include studies described purely as pilots this represented a rapid assessment of implementation of change – assessment undertaken after one week. Similar to plan-do-study-act cycle approaches. Focus on measures of target meeting for triage time.	Gray 2017(83)

Supplementary table 2: Primary studies in the UK

Study ID	Type of study	Rapid descriptor	Study question	Methods used	Data collected
Aspray 2006(84)	Mixed methods	Rapid evaluation	Equity of care diabetes standards in care homes	Interviews, checklists, record review	Quality of care metrics; experience of patients and carers
Banga 2018(85)	Quantitative	Rapid safety evaluation	Coverage and AE in influenza vaccination	Enhanced passive surveillance, spontaneous reporting, usage data	Uptake and AE (especially reporting within 7 days)
Begg 1989(86)	Quantitative	Rapid evaluation	Vaccination coverage of sentinel vaccines at key timepoints	Analysis of quarterly data cohorts	Proportion of eligible children immunised

For peer review only

Brook 2011(87)	Quantitative	Rapid cycle assessment	Sexual health clinic service evaluation	Electronic patient record analysis	Proportion of people treated and partners tested
Brown 2006(36)	Qualitative	Rapid participatory appraisal	Healthy environment - perceptions of need and concern	Documentation review, observations, interviews	Perceived needs and concerns around health-related needs (environment-based)
Cake 2022(88)	Quantitative	Rapid data-enabled access	Early recruitment to national clinical platform trial of COVID-19 community treatment	Use of Summary Care Record to enable near-real time recruitment of patients testing positive	Numbers of participants recruited to national trial
Cowan 2021(89)	Qualitative	Rapid prioritisation for rapid evaluation	Rapid topic prioritisation for rapid evaluation in adult social care	Adaptation of James Lind Alliance approach to rapid use	Rapid prioritisation of innovations for rapid evaluation; implications and necessary compromises
Crellin 2022(90)	Mixed methods	Rapid evaluation	Disparities of service research and patient engagement with home monitoring for COVID-19 services	Interviews and surveys with/of multiple stakeholders	Identification of strategies for inclusivity and reported disparities in experience and engagement with service
Dale 1996(23)	Qualitative	Rapid appraisal	Out of hours primary care strengths and weaknesses	Quantitative and qualitative survey methods	Availability of out of hours care and integration with other services
Farrer 2014(72)	Mixed methods	Rapid test changes	Changes in dietician assessments	Evaluation of ward data records	Use of new tool within designated time frame and accuracy of use
Fergus 2010(1)	Mixed methods	Rapid participatory appraisal	Palliative care out of hours provision	Routine data; semi-structured interviews; direct observations	Service provision and demand for services, barriers to accessing services
Gray 2017(91)	Mixed methods	Initial review	Rapid assessment of patients in acute medical unit	Staff feedback and information distribution	Triage of patients within timeframes, changes in times to assessment etc.

For peer review only

Green 2015(28)	Qualitative	Rapid appraisal	Reduced street lighting at night	Ethnographic data, household survey, document review	Public views on intervention
Guise 2018(79)	Qualitative	Rapid assessment	Remote hepatitis C testing	Focus groups and semi-structured interviews	Acceptability of approach, ease of use, other considerations including result management
Hamilton 1993(30)	Qualitative	Rapid appraisal	Planning for mental health provision	Semi-structured interviews	Service priorities for provision at borough level
Hanckel 2019(41)	Qualitative	Rapid ethnographic assessment	Daily mile in school	Interviews, observations, routine data	Implementation methods and perceived benefits
Joseph 2003(92)	Quantitative	Rapid reporting	Influenza vaccination	Data collection from records	Immunization uptake among population aged 65+
Lazenbatt 2001 (35)	Mixed methods	Rapid participatory appraisal	Women's access to health service provision	Overview of provision	Access to health services and related barriers
Lawlor 1999(38)	Qualitative	Rapid participatory appraisal	Sexual health needs in young people	Meta-planning	Needs in sexual health services planning
Lester 2002(93)	Mixed methods	Health inequality impact rapid appraisal	Impact of health inequalities on community and services	Complex multidisciplinary approaches	Cycle of deprivation – methods to break this
Newlands 2018(94)	Mixed methods	Rapid approach to knowledge translation	Quality improvement of pharmacy services	Nominal group technique, Delphi process	Priorities for knowledge translation into practice
Rowa-Dewar 2008(31)	Qualitative	Rapid appraisal approach	Public perceptions of cancer services	Multisite rapid appraisals in communities including those hard to reach	Views of cancer care in communities considered hard to reach

Supplementary Table 3: Primary studies in non-UK high income countries

Study ID	Country	Type of study	Rapid descriptor	Study question	Methods used	Data collected
Alguacil-Ramos 2019(95)	Spain	Quantitative	Rapid detection of safety signals	Adverse events of influenza vaccination	Population based register, real-time consultation	Specific adverse events, demographics impacted
Betsos 2021(50)	Canada	Qualitative	Rapid ethnographic study	Use of drug checking technologies	Fieldwork including interviews	Perceptions of efficacy
Bjornson-Benson 1993(96)	USA	Quantitative	Rapid feedback	Most effective trial recruitment methods	Real-time monitoring and rapid feedback on efficacy of different strategies	Proportion of participants recruited by each strategy
Boyd 2018(42)	Canada	Qualitative	Rapid ethnographic study	Supervised consumption sites for drug overdose preventions	Observations and interviews	Experiences of participants
Brown 2008(59)	USA	Mixed methods	Rapid assessment response and evaluation	Guiding interventions for urban health disparities	Mapping, observations, interviews, focus groups	Care received by key groups
Brown-Johnson 2019(97)	USA	Qualitative	Lightning report rapid qualitative synthesis	Barriers and facilitators of life-sustaining treatment decisions initiative	Interviews	Provision
Burks 2011(60)	USA	Qualitative	Rapid assessment	Barriers to prevention and treatment of HIV	Multiple triangulated methods with high user involvement	Barriers to accessing care

For peer review only

Cakir 2008(98)	Turkey	Quantitative	Rapid evaluation	Routine vaccination coverage	Survey, interviews	Vaccines received, reasons for non-vaccination
Campbell 2021(99)	Australia	Mixed methods	Emergency response priority research	Multiple projects	Infrastructure and processes including embedded researchers	Rapid evidence generation and translation
Carrillo 2022(100)	USA	Quantitative	Rapid acceleration	Standardisation of data collection for rapid COVID-19 research	Methods for standardisation, harmonisation and protection of data sharing	Success in improving data accessibility
CDC 2005(101)	USA	Quantitative	Rapid assessment	Vaccination coverage	Automated rapid analysis system	Adverse events, vaccination coverage, adherence to guidelines
Collins 2019(51)	Canada	Qualitative	Rapid ethnographic study	Impact of law enforcement on overdose preventions sites	Interviews and ethnographic fieldwork	Barriers to service access created by other services
Collins 2022(52)	USA	Qualitative	Rapid ethnographic study	COVID-19 public health measures impact on unstably housed drug users	Interviews and ethnographic fieldwork	Impact of public health measures on existing vulnerabilities
Corley 2021(102)	USA	Qualitative	Rapid research implementation	Impact of COVID-19 measures on clinical/community cohorts	Surveys	Mental health impact of COVID-19 associated measures on clinical and community cohorts
Currow 2012(103)	Australia & New Zealand	Quantitative	Rapid report	Metoclopramide	Data on use and symptoms in cohort	Benefit and adverse events, treatment adherence

For peer review only

D'Amore 2003(104)	USA	Quantitative	Brief report	Automatic therapeutic interchange programme	Pharmacy records review	Impact on outpatient therapy
Daviskiba 2021(105)	USA	Quantitative	Rapid assessment	Impact of COVID-19 on healthcare workers	Survey	Psychiatric symptoms, adequacy of PPE and training
Delaney 2019(106)	USA	Mixed methods	Rapid approach assessment	Low volume paediatric cardiac care programmes	Retrospective chart review	Quality of care metrics
Demeulemeester 2017(107)	Belgium	Quantitative	Rapid safety assessment	Seasonal influenza vaccination	Vaccination surveillance	Specified and unspecified adverse events
Douglas 2022(108)	USA	Quantitative	Rapid evaluation	Emerging therapies for pancreatic cancer	Platform trial	Efficacy and safety
Dudovitz 2021(109)	USA	Mixed methods	Rapid research	Research prioritisation for children and COVID-19	Rapid methods for research prioritisation – virtual meetings	Development of a research agenda to devise and test interventions
Dunbar 2018(110)	USA	Mixed methods	Brief research study	Connections to care programme	Interviews	effect on clients, government and community organisation spending
Dupont 2015(54)	Netherlands	Qualitative	Rapid assessment and response	Evidence based interventions for young people	Stepwise fieldwork	Use and patterns of use of cannabis
Eaves 2022(111)	USA	Qualitative	Rapid online ethnography (RARE)	Impact of COVID-19 policy on people who use drugs	Interviews and focus groups, observations	Perspectives and methodological challenges

For peer review only

Elsawy 2022(112)	Eastern Mediterranean countries	Quantitative	Rapid assessment	Impact of COVID-19 on mental health services	Survey of national mental health focal points	Impact of pandemic and responses to service restrictions
Elwy 2021(113)	USA	Qualitative	Rapid qualitative study	Vaccine hesitancy in COVID-19	Interviews and focus group	Views of vaccination, incidence of hesitancy
Foreman-Mackey 2019 (33)	Canada	Quantitative	Rapid evaluation	Overdose prevention sites	Semi-structured interviews	Experiences of people accessing smoking and injection services
Gale 2019(114)	USA	Qualitative	Rapid qualitative analysis	Evidence-based strategy in opioid prescribing	Interviews	Methods paper which focuses on comparison of rapid versus conventional qualitative analyses of interviews
Gagne 2013(115)	USA	Quantitative	Rapid assessment	Zoledronic acid adverse event	Analysis of data from commercial claims database	Myocardial infarctions in people taking zoledronic acid vs other bisphosphonates
Gawaya 2022(17)	Australia	Mixed methods	Rapid evaluation	Multiple programmes/practices	Team-based approach to rapid evaluation	Effectiveness and scalability
Goepp 2008(43)	USA	Qualitative	Rapid assessment	Barriers and facilitators to inpatient palliative care	Interviews	factors influencing service uptake
Gomez-Ramirez 2021(116)	Canada	Qualitative	Rapid qualitative assessment	Canada COVID-19 alert app	Walk-through of app; analysis of government documents and media coverage	Functionality, aims and purpose
Green 2013 (44)	USA	Qualitative	Rapid assessment	law enforcement overdose prevention	Interviews	Experiences of overdose prevention and naloxone

For peer review only

Hamarman 2014 (117)	USA	Qualitative	Rapid assessment	Sexually transmitted disease programmes	Interviews and observations	Service provision; integration and collaboration
Hasford 2020(118)	Germany	Quantitative	Rapid assessment	COVID-19 vaccines	Trial design	Large simple RCT benefits
Hensel 2016(119)	Canada	Mixed methods	Pragmatic randomised trial	Online platform for mental health self-management	Data analysis of trial outcomes; participant interviews	Recovery at 3 months
Im 2022(120)	USA	Qualitative	Rapid evaluation and appraisal	Impact of COVID-19 in refugee service provision and support	Interviews and focus groups, survey	Gaps in service provision and support
Keniston 2022(121)	USA	Qualitative	Rapid qualitative assessment	Inpatient surge planning and workforce deployment in COVID-19	Focus groups	Adaptations and associated issues in workforce planning and deployment
Kercsmar 2017 (71)	USA	Quantitative	Rapid evaluation	Paediatric asthma care redesign	Control charts (annotated)	Hospitalisations and ED visits
Khalid-Khan 2020(122)	Canada	Qualitative	Rapid-cycle program evaluation	Child and adolescent psychiatry programme	Focus groups and interviews	Identified improvements or refinements
Khubchandi 2021(123)	USA	Quantitative	Rapid national assessment	COVID-19 vaccine hesitancy	Survey	Likelihood of vaccination f
Kieber-Emmons 2018(2)	USA	Mixed methods	Rapid assessment	Care for cancer patients/survivors	Geographic information systems, observations, Interviews	Mortality rates, insurance coverage, provision of care, delivery methods
Kieber-Emmons 2022(78)	USA	Mixed methods	Focused rapid assessment process	Care for cancer patients/survivors	Geographic information systems, observations, Interviews	Modifiable factors in survivorship care

For peer review only

Kobau 2022(124)	USA	Qualitative	Rapid community assessment	Vaccine confidence in COVID-19	Interviews, listening sessions, observations, street intercept surveys	Barriers and facilitators to uptake
Kroeger 2013 (47)	USA	Mixed methods	Rapid ethnographic assessment	Sexual health services	Interviews	Views on available services/potential changes to services
Kuhlman 2021(125)	European Union	Mixed methods	Rapid assessment	Health workforce protection and preparedness in COVID-19	Assessment tool, secondary data sources, case studies	Actions taken and required, differences across areas of preparedness
Livorsi 2016 (126)	USA	Mixed methods	Rapid assessment	Barriers and facilitators to safety culture in an intensive care unit	Interviews	Reporting errors, approachability of authority figures and handovers.
Loosier 2020 (127)	USA	Qualitative	Rapid ethnographic assessment	Sexual health services	Interviews	Financial and care capability parameters
Maaskant 2021(128)	Netherlands	Qualitative	Rapid qualitative study	Isolation and families of hositalised COVID-19 patients	Patient record review and focus groups	Practices and nurse experiences
MacCarthy 2019 (129)	USA	Qualitative	Rapid assessment process	HIV prevention information	Interviews	HIV prevention information on mobile technology
McDonald 2022(130)	11 European countries	Quantitative	Rapid assessment	Take-home naloxone provision during COVID-19	Programme reporting instrument, survey	Capacity and adaptations
McNall 2004(131)	USA	Mixed methods	Rapid feedback evaluation	Retention in an HIV/AIDs intervention	Data collection and analysis of approaches	Retention rates and the factors impacting them

For peer review only

Minoyan 2022(132)	Canada	Mixed methods	Rapid assessment	Impact of COVID-19 health emergency declaration	Questionnaire and interviews	Access to health services, behaviour and experience
Moloney 2020(76)	USA	Qualitative	Rapid assessment procedure informed clinical ethnography case series	Clinical trial of interventions for traumatic injury conducted during COVID-19	Observations of multiple clinical situations and team processes	Integration of COVID-19 prevention strategies into clinical interventions in research context
Murray 2022(133)	Multinational	Quantitative	Rapid evaluation	Therapeutics for COVID-19 inpatients	Platform trial master protocol	Safety and efficacy
Murphy 2018 (32)	Georgia	Mixed methods	Rapid appraisal	Barriers to mental health services	Document review; semi-structured interviews, triangulation	Barriers to access for internally displaced populations
Nevedal 2021(134)	USA	Qualitative	Rapid qualitative analysis	Veterans Health Administration programme to identify and diffuse evidence-based interventions.	Interviews	Methods paper which focuses on comparison of rapid versus conventional qualitative analyses of interviews
Oh 2021(53)	USA	Qualitative	Rapid ethnographic assessment	Professional trearment and recovery resources for alcohol use	Observations and interviews	Perceptions of service use
Palinkas 2020(135)	USA	Qualitative	Rapid ethnographic assessment	Service delivery in acute care and trauma: impact of COVID-19	Observations by clinical participants, conversation summaries	Impacts on service delivery
Palinkas 2021a(136)	USA	Qualitative	Rapid assessment procedure informed clinical ethnography	Child and adolescent mental health practice: impact of COVID-19	Interviews, stakeholder contact	Impacts on service delivery and demand

For peer review only

Palinkas 2021b(137)	USA	Qualitative	Rapid assessment procedure informed clinical ethnography case series	Disaster preparedness: impact of COVID-19	Interviews, immersion techniques	Impacts on preparedness, strengths and capabilities, integrated responses
Palinkas 2022(77)	USA	Qualitative	Rapid assessment procedure informed clinical ethnography case series	Service delivery in acute care and trauma: impact of COVID-19	Observations by clinical participants, conversation summaries	Ongoing impacts and impact of service changes
Quintero Romero 2006 (46)	Italy	Mixed methods	Rapid ethnographic study	Information and support for breastfeeding	Interviews	Access to and need for support, experiences of services
Rains 2020(138)	USA	Mixed methods	Rapid cycle testing	Geriatric acute unplanned care	Routine data analysis and feedback	Unplanned healthcare episodes, costs, satisfaction
Renfro 2022(139)	USA	Qualitative	Rapid assessment procedure	Medication therapy management in primary care	Interviews	Implications for change from programme adoption
Rodriguez 2021(140)	USA	Mixed methods	Rapid evaluation	COVID-19 vaccination in ED for underserved patients	Survey	Vaccine hesitancy, vaccination availability, reasons for hesitancy
Rolf 2021(141)	Australia	Qualitative	Large-scale rapid analysis	Australian border and biosecurity policy in COVID-19	Survey and interviews	Success in using rapid methods
Romeu-Labayen 2022(142)	Spain	Qualitative	Rapid research evaluation and appraisal	PPE provision for nurses in COVID-19	Interviews	Experiences and perceptions
Rosteius 2022(143)	Netherlands	Qualitative	Rapid ethnographic research	Nursing home staff	Using existing relationships to enable rapid ethnography	Impact of long-term relationships on rapid work

For peer review only

Scott 2022(144)	USA	Mixed methods	Rapid assessment procedure informed clinical ethnography	Opioid treatment programmes	Interviews, surveys and site visits	Contextual factors impacting implementation of trial protocol
Seay 2017(145)	USA	Quantitative	Rapid assessment	Transgender experience and use of cervical screening	Survey	Uptake, causes of low uptake
Seay 2015(146)	USA	Quantitative	Rapid assessment	cancer screening in immigrant communities	Survey	Uptake and access disparities
Shaw 2022(147)	USA	Qualitative	Rapid research and evaluation methods	Telehealth in patients following sexual assault	Interviews	Experience including successes and challenges
Shimkhada 2021(148)	USA	Qualitative	Rapid assessment	Support in metastatic breast cancer	Twitter chat	Barriers to care and support
Short 2022(149)	USA	Quantitative	Rapid parallel evaluation	Relapsed/refractory acute myeloid leukaemia	Platform trial (phase II)	Efficacy
Skillman 2018(68)	USA	Mixed methods	Rapid cycle evaluation	Payment and service models for Medicare/Medicaid	Interviews and focus groups, site visits	Cost, quality, use and experiences
Solomon 2006(150)	USA	Qualitative	Rapid assessment procedure	HIV prevention programming in Community Mental Health Centre	Interviews, focus groups, ecological assessment	Availability and perception of availability of services and materials
Sperber 2019(62)	USA	Qualitative	Rapid qualitative assessment	Walking programme for hospitalised older adults	interview, document review	Impacts of early stage implementation

For peer review only

Srinivasan 2020(151)	USA	Qualitative	Rapid qualitative analytic approach	Transition to video call primary care appointments	Interviews	Critical issues to enable sustainability of change
Talati 2021(152)	USA	Quantitative	Rapid assessment	Telehealth for maternity care	Survey	Satisfaction
Talwai 2021(153)	USA	Quantitative	Rapid research	Multiple COVID-19 projects	Data repository	Data usage in research
Tan 2021(154)	Singapore	Qualitative	Rapid qualitative research	Health and social care need in COVID-19 public health measures context	Interviews, expanded field notes	Sexual health service provision and need
Taubenberger 2021(155)	USA	Qualitative	Rapid cycle assessment	Services for opioid use and overdose prevention	Interviews and observations	Knowledge and perception of services
Tort-Nasarre 2021(156)	Spain	Qualitative	Rapid research evaluation and appraisal	Nurse response to organisational changes in COVID-19	Interviews	Responses to change
Toth 2021(157)	Hungary	Quantitative	Rapid assessment of avoidable blindness	Avoidable blindness costs in diabetic retinopathy	Derived costs of treatment and prevalent illness data	Costs
Valderrama 2006 (61)	Spain	Qualitative	Rapid assessment	Drug abuse prevention needs of young people	Interviews (from rapid assessment and response evaluation methods)	Coverage of mandated prevention needs
Vazquez 2022(158)	USA	Qualitative	Rapid qualitative study	Risk-reduction measures for COVID-19 in institutions	Focus groups	Perceptions of stakeholders
Wesolowski 2020(159)	USA	Quantitative	Rapid assessments	Adoption of non-pharmaceutical interventions for COVID-19	Online panel	Use of non-pharmaceutical interventions, positive test results

Wichmann 2010 (160)	Germany	Quantitative	Rapid assessment	Pandemic influenza vaccination effectiveness	Data records of influenza cases vaccination status	Effectiveness estimates – breakthrough infections
Williams 2019 (161)	USA	Mixed methods	Rapid gap assessment	Sexual health services coverage	Site visits, checklists, online data sources, open-ended questioning	Service provision in urgent care centres; challenges experienced
Wiss 2019 (162)	USA	Quantitative	Rapid assessment	nutritional services in drugs misuse treatment services	Survey	Service provision and characteristics
Xiang 2021(163)	USA	Quantitative	Streamlined eligibility assessment	Recruitment for hematology and oncology research studies	Implementation of standardised prescreening protocol	Enrolment to clinical trials
Zakocs 2015(69)	USA	Mixed methods	Rapid feedback cycle	Primary prevention programs for domestic violence	Use of data-to-action framework	Impact of methods; capacity building

For peer review only

Supplementary Table 4: Studies assessing implementation in high income countries

Study ID	Country	Type of study	Rapid descriptor	Study question	Results
Agaku 2022(164)	USA	Quantitative	Rapid evaluation	Federal and state tobacco law	Access and equity of impact
Ash 2008 (165)	USA	Mixed methods	Rapid assessment process	Informatics in clinical decision support	implementation for rapid assessment process and actionable feedback
Belford 2017 (166)	UK	Mixed methods	Evaluability assessment giving quick feedback	Community development programme to reduce health inequality	Stakeholder involvement to produce logic model of programme theory and evaluation opinions
Cohn 2021(65)	USA	Qualitative	Rapid evaluation using consolidated framework for implementation research	Mobile health use in HIV care settings	Identification of barriers and facilitators, both general and intervention specific.
Haigh 2013 (167)	Australia and New Zealand	Quantitative	Rapid health impact assessment	Use of health impact reports including rapid assessments	Quality assessment of reports to develop improvements in practice
Hailey 2001 (168)	Canada	Quantitative	Rapid assessment process	Rapid HTAs	Rapid HTA programme in provincial healthcare system in response to urgent requests for advice
Holdsworth 2020 (8)	USA	Mixed methods	Rapid assessment	ICU redesign for patient safety	Implementation methods and perceived benefits; barriers and facilitators
Keith 2017 (63)	USA	Qualitative	Rapid cycle evaluation	Barriers and facilitators to different comprehensive primary care initiative components	Consolidated framework for implementation research useful for rapid cycle

For peer review only

					evaluation of practice transformation initiatives
Last 2021(66)	USA	Mixed methods	Rapid participatory methods, rapid prototyping	Depression screening in primary care	Engagement of diverse stakeholders, production of acceptable and feasible implementation strategy
Lewinski 2021(169)	USA	Qualitative	Rapid qualitative analysis	Care coordination in rural areas	Informing near-real time implementation of systems is possible using these methods
Li 2021(170)	USA	Quantitative	Rapid assessment	Assessment of COVID-19 vaccination implementation	Event study of phased implementation process informs policy
Ohrling 2020(67)	Sweden	Mixed methods	Rapid implementation action research case study	Emergency response to COVID-19	Changes in health -care services and success of implementaiton
Sperber 2019 (62)	USA	Qualitative	Rapid qualitative assessment	Early strategies to implement evidence-based walking program for hospitalized older adults	Lessons from early stage implementation
Wright 2015 (3)	USA	Qualitative	Rapid assessment process	Best practice for a clinical decision support system	Challenges and lessons for implementation

For peer review only

References for supplementary material

1. Fergus CJ, Chinn DJ, Murray SA. Assessing and improving out-of-hours palliative care in a deprived community: a rapid appraisal study. Palliative Medicine. 2010;24(5):493-500.
2. Kieber-Emmons A, Crabtree BF, Miller W. A new mixed methods approach to uncover multi-level barriers and facilitators of cancer survivorship. Journal of Clinical Oncology Conference. 2018;36(7).
3. Wright A, Sittig DF, Ash JS, Erickson JL, Hickman TT, Paterno M, et al. Lessons learned from implementing service-oriented clinical decision support at four sites: A qualitative study. International Journal of Medical Informatics. 2015;84(11):901-11.
4. Manderson L, Aaby P. An epidemic in the field? Rapid assessment procedures and health research. Social Science and Medicine. 1992;35(7):839-50.
5. McMullen CK, Ash JS, Sittig DF, Bunce A, Guappone K, Dykstra R, et al. Rapid assessment of clinical information systems in the healthcare setting: an efficient method for time-pressed evaluation. Methods of information in medicine. 2011;50(4):299-307.
6. Needle RH, Trotter IRT, Goosby E, Bates C, Von Zinkernagel D. Methodologically sound rapid assessment and response: Providing timely data for policy development on drug use interventions and HIV prevention. International Journal of Drug Policy. 2000;11(1-2):19-23.
7. Needle RH, Trotter RT, 2nd, Singer M, Bates C, Page JB, Metzger D, et al. Rapid assessment of the HIV/AIDS crisis in racial and ethnic minority communities: an approach for timely community interventions. American Journal of Public Health. 2003;93(6):970-9.
8. Holdsworth LM, Safaeinili N, Winget M, Lorenz KA, Lough M, Asch S, et al. Adapting rapid assessment procedures for implementation research using a team-based approach to analysis: a case example of patient quality and safety interventions in the ICU. Implementation Science. 2020;15(1):12.
9. Peiro R. Rapid appraisal methodology for 'health for all' policy formulation analysis. 2003.
10. Peiro R, Alvarez-Dardet C, Plasencia A, Borrell C, Colomer C, Moya C, et al. Rapid appraisal methodology for 'health for all' policy formulation analysis. Health Policy. 2002;62(3):309-28.
11. Fitch C, Rhodes T, Stimson GV. Origins of an epidemic: The methodological and political emergence of rapid assessment. International Journal of Drug Policy. 2000;11(1-2):63-82.
12. Fitch C, Stimson GV, Rhodes T, Poznyak V. Rapid assessment: an international review of diffusion, practice and outcomes in the substance use field. Soc Sci Med. 2004;59(9):1819-30.
13. Stimson GV, Fitch C, DesJarlais D, Poznyak V, Perlis T, Oppenheimer E, et al. Rapid assessment and response studies of injection drug use: knowledge gain, capacity building, and intervention development in a multisite study. American Journal of Public Health. 2006;96(2):288-95.
14. Walton H, Vindrola-Padros C, Crellin NE, Sidhu MS, Herlitz L, Litchfield I, et al. Patients' experiences of, and engagement with, remote home monitoring services for COVID-19 patients: A rapid mixed-methods study. Health Expectations.n/a(n/a).
15. Humphries K, Maxwell S, Bartholomew S, Clutterbuck D. Rapid service evaluation of in-house blood self-sampling for interval STI testing for PrEP cohort at minimal cost. HIV Medicine. 2021;22(SUPPL 2):82-3.
16. Vindrola-Padros C, Ledger J, Hill M, Tomini S, Spencer J, Fulop NJ. The Special Measures for Quality and Challenged Provider Regimes in the English NHS: A Rapid Evaluation of a National Improvement Initiative for Failing Healthcare Organisations. International Journal of Health Policy & Management. 2022;27:27.
17. Gawaya M, Terrill D, Williams E. Using rapid evaluation methods to assess service delivery changes: Lessons learned for evaluation practice during the COVID-19 pandemic. Evaluation Journal of Australasia. 2022;22(1):30-48.
18. Sidhu M, Pollard J, Sussex J. Vertical integration of primary care practices with acute hospitals in England and Wales: why, how and so what? Findings from a qualitative, rapid evaluation. BMJ Open. 2022;12(1):e053222.

For peer review only

19. Parkinson S, Smith J, Sidhu M. Early development of primary care networks in the NHS in England: A qualitative mixed-methods evaluation. BMJ Open. 2021;11(12) (no pagination)(e055199).
20. Watson D, Baralle NL, Alagil J, Anil K, Ciccognani S, Dewar-Haggart R, et al. How do we engage people in testing for COVID-19? A rapid qualitative evaluation of a testing programme in schools, GP surgeries and a university. BMC Public Health. 2022;22(1):305.
21. Rhodes T, Stimson GV, Fitch C, Ball A, Renton A. Rapid assessment, injecting drug use, and public health. The Lancet. 1999;354(9172):65-8.
22. Campbell J. A critical appraisal of participatory methods in development research. International Journal of Social Research Methodology. 2002;5(1):19-29.
23. Dale J, Shipman C, Lacock L, Davies M. Creating a shared vision of out of hours care: using rapid appraisal methods to create an interagency, community oriented, approach to service development. Bmj. 1996;312(7040):1206-10.
24. Murray SA. Listening to local voices : adapting rapid appraisal to assess health and social needs in general practice. 1994.
25. Murray SA. Experiences with "rapid appraisal" in primary care: involving the public in assessing health needs, orientating staff, and educating medical students. Bmj. 1999;318(7181):440-4.
26. Harvey HD, Fleming P. A rapid appraisal method for the selection and pre-testing of environmental health leaflets. Journal of The Royal Society for the Promotion of Health. 2000;120(2):112-6.
27. Harvey HD, Fleming P, Patterson M. A rapid appraisal method for reviewing the effectiveness of workplace smoking policies in large and medium sized organisations. Journal of The Royal Society for the Promotion of Health. 2001;121(1):50-5.
28. Green J, Perkins C, Steinbach R, Edwards P. Reduced street lighting at night and health: A rapid appraisal of public views in England and Wales. Health Place. 2015;34:171-80.
29. Beebe J, Given L. Rapid assessment process. The sage encyclopedia of qualitative research methods

Thousand Oaks, California: SAGE Publications; 2012.
30. Hamilton S. Felt needs assessment of mental health (rapid service appraisal): Barking and Havering. (Public health research report, ISSN 09689818 ; no 11). 1993:31.
31. Rowa-Dewar N, Ager W, Ryan K, Hargan I, Hubbard G, Kearney N. Using a rapid appraisal approach in a nationwide, multisite public involvement study in Scotland. Qualitative Health Research. 2008;18(6):863-9.
32. Murphy A, Chikovani I, Uchaneishvili M, Makhashvili N, Roberts B. Barriers to mental health care utilization among internally displaced persons in the republic of Georgia: a rapid appraisal study. BMC health services research. 2018;18(1):306.
33. Foreman-Mackey A, Bayoumi AM, Miskovic M, Kolla G, Strike C. 'It's our safe sanctuary': Experiences of using an unsanctioned overdose prevention site in Toronto, Ontario. International Journal of Drug Policy. 2019;73:135-40.
34. Beebe J. Rapid assessment and response: Sound methodology for producing timely responses. International Journal of Drug Policy. 2000;11(1-2):29-31.
35. Lazenbatt A, Lynch U, O'Neill E. Revealing the hidden 'troubles' in Northern Ireland: The role of participatory rapid appraisal. Health Education Research. 2001;16(5):567-78.
36. Brown CS, Lloyd S, Murray SA. Using consecutive Rapid Participatory Appraisal studies to assess, facilitate and evaluate health and social change in community settings. BMC Public Health. 2006;6 (no pagination)(68).
37. Annett H. Guidelines for rapid participatory appraisals to assess community health needs: a focus on health improvements for low-income urban and rural areas. Geneva: Division of Strengthening of Health Services World Health Organization available from HMSO; 1995. iv,60 p.
38. Lawlor D. Rapid participatory appraisal of young people's sexual health needs: an evaluation of meta-planning. 1999.

For peer review only

39. Desai A, Donetto S. Evolving ethnographic sensibilities: Using actor-network theory in health services research. BMJ Open. 2019;9 (Supplement 1):A5.
40. Vindrola-Padros C, Vindrola-Padros B. Quick and dirty? A systematic review of the use of rapid ethnographies in healthcare organisation and delivery. BMJ Qual Saf. 2018;27(4):321-30.
41. Hanckel B, Ruta D, Scott G, Peacock JL, Green J. The Daily Mile as a public health intervention: a rapid ethnographic assessment of uptake and implementation in South London, UK. BMC Public Health. 2019;19(1):1167.
42. Boyd J, Collins AB, Mayer S, Maher L, Kerr T, McNeil R. Gendered violence and overdose prevention sites: a rapid ethnographic study during an overdose epidemic in Vancouver, Canada. Addiction. 2018;113(12):2261-70.
43. Goepp JG, Meykler S, Mooney NE, Lyon C, Raso R, Julliard K. Provider insights about palliative care barriers and facilitators: results of a rapid ethnographic assessment. Am J Hosp Palliat Care. 2008;25(4):309-14.
44. Green TC, Zaller N, Palacios WR, Bowman SE, Ray M, Heimer R, et al. Law enforcement attitudes toward overdose prevention and response. Drug Alcohol Depend. 2013;133(2):677-84.
45. Ackerman S, Sarkar U, Tieu L, Hamdley M, Schillinger D, Jahn K, et al. Meaningful use in the safety net: A rapid ethnography of patient portal implementation at five community health centers in California. Journal of the American Medical Informatics Association. 2017;24(5):903-12.
46. Quintero Romero S, Bernal R, Barbiero C, Passamonte R, Cattaneo A. A rapid ethnographic study of breastfeeding in the North and South of Italy. Int Breastfeed J. 2006;1:14.
47. Kroeger K, Willilams S, Alexander-Pender C, Ford J, Vasani V. "Just one thing on the plate" - Views of community members and providers on sexual health services in the fort mcpherson area of Southwest Atlanta: Findings from a rapid ethnographic assessment. Sexually Transmitted Infections Conference: STI and AIDS World Congress. 2013;89(SUPPL. 1).
48. Ayele R, Manges KA, Leonard C, Lee M, Galenbeck E, Molla M, et al. How Context Influences Hospital Readmissions from Skilled Nursing Facilities: A Rapid Ethnographic Study. Journal of the American Medical Directors Association. 2021;22(6):1248-54.e3.
49. Manges KA, Ayele R, Leonard C, Lee M, Galenbeck E, Burke RE. Differences in transitional care processes among high-performing and low-performing hospital-SNF pairs: a rapid ethnographic approach. BMJ Quality & Safety. 2021;30(8):648-57.
50. Betsos A, Valleriani J, Boyd J, Bardwell G, Kerr T, McNeil R. "I couldn't live with killing one of my friends or anybody": A rapid ethnographic study of drug sellers' use of drug checking. International Journal of Drug Policy. 2021;87:102845.
51. Collins AB, Boyd J, Mayer S, Fowler A, Kennedy MC, Bluthenthal RN, et al. Policing space in the overdose crisis: A rapid ethnographic study of the impact of law enforcement practices on the effectiveness of overdose prevention sites. International Journal of Drug Policy. 2019;73:199-207.
52. Collins AB, Edwards S, McNeil R, Goldman J, Hallowell BD, Scagos RP, et al. A rapid ethnographic study of risk negotiation during the COVID-19 pandemic among unstably housed people who use drugs in Rhode Island. International Journal of Drug Policy. 2022;103:103626.
53. Oh H, Yamada AM. Exploring second generation Korean American alcohol use through church-based participatory research: A rapid ethnographic assessment in Los Angeles, California, United States. Health & Social Care in the Community. 2021;29(5):1359-67.
54. Dupont HB, Kaplan CD, Braam RV, Verbraeck HT, de Vries NK. The application of the rapid assessment and response methodology for cannabis prevention research among youth in the Netherlands. International Journal of Drug Policy. 2015;26(8):731-8.
55. Ong BN. Rapid appraisal and health policy. 1996.
56. Ong BN, Humphris G, Annett H, Rifkin S. Rapid appraisal in an urban setting, an example from the developed world. Soc Sci Med. 1991;32(8):909-15.
57. Organization WH, UNAIDS. SEX-RAR Guide. https://apps.who.int/iris/bitstream/handle/10665/42681/9241545585.pdf?sequence=1&isAllowed=y2002.

For peer review only

58. Trotter IRT, Needle R, Goosby E, Bates C, Singer M. A Methodological Model for Rapid Assessment, Response, and Evaluation: The RARE Program in Public Health. Field Methods. 2001;13(2):137-59.
59. Brown DR, Hernandez A, Saint-Jean G, Evans S, Tafari I, Brewster LG, et al. A participatory action research pilot study of urban health disparities using rapid assessment response and evaluation. American Journal of Public Health. 2008;98(1):28-38.
60. Burks DJ, Robbins R, Durtschi JP. American Indian gay, bisexual and two-spirit men: a rapid assessment of HIV/AIDS risk factors, barriers to prevention and culturally-sensitive intervention. Culture, health & sexuality. 2011;13(3):283-98.
61. Valderrama JC, Tortajada S, Alapont L, Vidal A, Perez Ma J, Castellano M, et al. Rapid assessment of drug abuse prevention needs for youth in small Spanish municipalities: Coping with resource limitations. Journal of Drug Issues. 2006;36(1):29-52.
62. Sperber NR, Bruening RA, Choate A, Mahanna E, Wang V, Powell BJ, et al. Implementing a Mandated Program Across a Regional Health Care System: A Rapid Qualitative Assessment to Evaluate Early Implementation Strategies. Quality Management in Health Care. 2019;28(3):147-54.
63. Keith RE, Crosson JC, O'Malley AS, Cromp D, Taylor EF. Using the Consolidated Framework for Implementation Research (CFIR) to produce actionable findings: a rapid-cycle evaluation approach to improving implementation. Implement Sci. 2017;12(1):15.
64. Shrank WH. The Center For Medicare And Medicaid Innovation's blueprint for rapid-cycle evaluation of new care and payment models. Health Affairs (Millwood). 2013;32(4):807-12.
65. Cohn WF, Canan CE, Knight S, Waldman AL, Dillingham R, Ingersoll K, et al. An Implementation Strategy to Expand Mobile Health Use in HIV Care Settings: Rapid Evaluation Study Using the Consolidated Framework for Implementation Research. JMIR MHealth and UHealth. 2021;9(4):e19163.
66. Last BS, Buttenheim AM, Futterer AC, Livesey C, Jaeger J, Stewart RE, et al. A pilot study of participatory and rapid implementation approaches to increase depression screening in primary care. BMC Family Practice. 2021;22(1):228.
67. Ohrling M, Ovretveit J, Lockowandt U, Brommels M, Sparring V. Management of the emergency response to the SARS-CoV-2 (COVID-19) outbreak in Stockholm, Sweden, and winter preparations. Journal of Primary Health Care. 2020;12(3):207-14.
68. Skillman M, Cross-Barnet C, Friedman Singer R, Rotondo C, Ruiz S, Moiduddin A. A Framework for Rigorous Qualitative Research as a Component of Mixed Method Rapid-Cycle Evaluation. Qualitative Health Research. 2018;29(2):279-89.
69. Zakocs R, Hill JA, Brown P, Wheaton J, Freire KE. The Data-to-Action Framework: A Rapid Program Improvement Process. Health Educ Behav. 2015;42(4):471-9.
70. Fergus CJY. Assessing and improving out-of-hours palliative care in a deprived community: a rapid appraisal study. 2010.
71. Kercsmar CM, Beck AF, Sauers-Ford H, Simmons J, Wiener B, Crosby L, et al. Association of an asthma improvement collaborative with health care utilization in medicaid-insured pediatric patients in an urban community. JAMA Pediatrics. 2017;171(11):1072-80.
72. Farrer K, Donaldson E, Blackett B, Lloyd H, Forde C, Melia D, et al. Nutritional screening of elderly patients: a health improvement approach to practice. J Hum Nutr Diet. 2014;27(2):184-91.
73. Schneeweiss S, Shrank WH, Ruhl M, Maclure M. Decision-making aligned with rapid-cycle evaluation in health care. International Journal of Technology Assessment in Health Care. 2015;31(4):214-22.
74. Palinkas LA, Mendon SJ, Hamilton AB. Innovations in Mixed Methods Evaluations. Annual Review of Public Health. 2019;40:423-42.
75. Palinkas LA, Zatzick D. Rapid Assessment Procedure Informed Clinical Ethnography (RAPICE) in Pragmatic Clinical Trials of Mental Health Services Implementation: Methods and Applied Case Study. Administration & Policy in Mental Health. 2019;46(2):255-70.

For peer review only

76. Moloney K, Scheuer H, Engstrom A, Schreiber M, Whiteside L, Nehra D, et al. Experiences and Insights from the Early US COVID-19 Epicenter: A Rapid Assessment Procedure Informed Clinical Ethnography Case Series. Psychiatry. 2020;83(2):115-27.
77. Palinkas LA, Engstrom A, Whiteside L, Moloney K, Zatzick D. A Rapid Ethnographic Assessment of the Impact of the COVID-19 Pandemic on Mental Health Services Delivery in an Acute Care Medical Emergency Department and Trauma Center. Administration & Policy in Mental Health. 2022;49(2):157-67.
78. Kieber-Emmons AM, Miller WL, Rubinstein EB, Howard J, Tsui J, Rankin JL, et al. A Novel Mixed Methods Approach Combining Geospatial Mapping and Qualitative Inquiry to Identify Multilevel Policy Targets: The Focused Rapid Assessment Process (fRAP) Applied to Cancer Survivorship. Journal of Mixed Methods Research. 2022;16(2):183-206.
79. Guise A, Witzel TC, Mandal S, Sabin C, Rhodes T, Nardone A, et al. A qualitative assessment of the acceptability of hepatitis C remote self-testing and self-sampling amongst people who use drugs in London, UK. BMC Infectious Diseases. 2018;18 (1) (no pagination)(281).
80. Phillips CB, Dwan K, Hepworth J, Pearce C, Hall S. Using qualitative mixed methods to study small health care organizations while maximising trustworthiness and authenticity. BMC Health Services Research. 2014;14:559.
81. Williams SP, Kinsey J, Carry MG, Terry L, Wells J, Kroeger K. Get In, Get Tested, Get Care: STD Services in Urban Urgent Care Centers. Sexually Transmitted Diseases. 2019;46(10):648-53.
82. Dunet DO, Sparling PB, Hersey J, Williams-Piehota P, Hill MD, Hanssen C, et al. A new evaluation tool to obtain practice-based evidence of worksite health promotion programs. Preventing chronic disease. 2008;5(4):A118.
83. Gray TA, Dumville JC, Christie J, Cullum NA. Rapid research and implementation priority setting for wound care uncertainties. PLoS ONE. 2017;12(12):e0188958.
84. Aspray TJ, Nesbit K, Cassidy TP, Hawthorne G. Rapid assessment methods used for health-equity audit: Diabetes mellitus among frail British care-home residents. Public Health. 2006;120(11):1042-51.
85. Banga S, Chabanon AL, Eymin C, Caroe T, Butler K, Moureau A. Near real-time safety surveillance of three seasonal influenza vaccines in Europe during influenza season 2017/18. Pharmacoepidemiology and Drug Safety. 2018;27 (Supplement 2):396.
86. Begg NT, Gill ON, White JM. COVER (Cover Of Vaccination Evaluated Rapidly): Description of the England and Wales scheme. Public Health. 1989;103(2):81-9.
87. Brook MG, Rusere L, Coppin-Browne L, McDonagh S, McSorley J. A prospective study of the effectiveness of electronic patient records in rapid-cycle assessment of treatment and partner notification outcomes for patients with genital chlamydia and gonorrhoea infection. Sexually Transmitted Infections. 2011;87(2):152-5.
88. Cake C, Ogburn E, Pinches H, Coleman G, Seymour D, Woodard F, et al. Development and evaluation of rapid data-enabled access to routine clinical information to enhance early recruitment to the national clinical platform trial of COVID-19 community treatments. Trials [Electronic Resource]. 2022;23(1):62.
89. Cowan K, Fulop NJ, Harshfield A, Ng PL, Ntouva A, Sidhu M, et al. Rapid prioritisation of topics for rapid evaluation: the case of innovations in adult social care and social work. Health Research Policy & Systems. 2021;19(1):34.
90. Crellin NE, Herlitz L, Sidhu MS, Ellins J, Georghiou T, Litchfield I, et al. Examining disparities relating to service reach and patient engagement with COVID-19 remote home monitoring services in England: a mixed methods rapid evaluation. medRxiv. 2022;22.
91. Gray A, Baker K. Assessment for change. Acute Medicine. 2017;16 (3):127-8.
92. Joseph C, Goddard N. Influenza vaccine uptake in the elderly: Results from a rapid assessment of the effectiveness of new government policy in England for the winters 2000/2001 and 2001/2002. Vaccine. 2003;21(11-12):1137-48.
93. Lester C. Priority setting with Health Inequality Impact Assessment. 2002.

For peer review only

94. Newlands RS, Power A, Young L, Watson M. Quality improvement of community pharmacy services: a prioritisation exercise. International Journal of Pharmacy Practice. 2018;26(1):39-48.
95. Alguacil-Ramos AM, Portero-Alonso A, Pastor-Villalba E, Muelas-Tirado J, Diez-Domingo J, Sanchis-Ferrer A, et al. Rapid assessment of enhanced safety surveillance for influenza vaccine. Public Health. 2019;168:137-41.
96. Bjornson-Benson WM, Stibolt TB, Manske KA, Zavela KJ, Youtsey DJ, Sonia Buist A. Monitoring recruitment effectiveness and cost in a clinical trial. Controlled Clinical Trials. 1993;14(2, Supplement):52-67.
97. Brown-Johnson C, Lo N, Giannitrapani K, Lowery J, Foglia MB, Walling A, et al. A Rapid Qualitative Synthesis of Insights from the Veterans Administration Life-Sustaining Treatment Decisions Initiative (LSTDI) National Implementation (QI703). Journal of Pain and Symptom Management. 2019;57 (2):457.
98. Cakir B, Uner S, Temel F, Akin L. Lot quality survey: An appealing method for rapid evaluation of vaccine coverage in developing countries - Experience in Turkey. BMC Public Health. 2008;8 (no pagination)(240).
99. Campbell D, Edwards B, Milat A, Thackway S, Whittaker E, Goudswaard L, et al. NSW Health COVID-19 Emergency Response Priority Research program: a case study of rapid translation of research into health decision making. Public Health Research & Practice. 2021;31(4):10.
100. Carrillo GA, Cohen-Wolkowiez M, D'Agostino EM, Marsolo K, Wruck LM, Johnson L, et al. Standardizing, harmonizing, and protecting data collection to broaden the impact of COVID-19 research: the rapid acceleration of diagnostics-underserved populations (RADx-UP) initiative. Journal of the American Medical Informatics Association. 2022;29(9):1480-8.
101. Centers for Disease C, Prevention. Rapid assessment of influenza vaccination coverage among HMO members--northern California influenza seasons, 2001-02 through 2004-05. MMWR Morb Mortal Wkly Rep. 2005;54(27):676-8.
102. Corley SS, Gillezeau C, Molina L, Alpert N, Eugene A, Lieberman-Cribbin W, et al. Using Rapid Research Implementation and Collaborations to Assess the Mental Health Impact of the COVID-19 Pandemic Among Community and Clinical Cohorts. Disaster Medicine & Public Health Preparedness. 2021:1-5.
103. Currow DC, Vella-Brincat J, Fazekas B, Clark K, Doogue M, Rowett D. Pharmacovigilance in hospice/palliative care: Rapid report of net clinical effect of metoclopramide. Journal of Palliative Medicine. 2012;15(10):1071-5.
104. D'Amore M, Masters P, Maroun C. Impact of an automatic therapeutic interchange program on discharge medication selection. Hospital Pharmacy. 2003;38(10):942-6.
105. Daviskiba SE, MacKenzie MA, Dow M, Johnston P, Balon R, Javanbakht A, et al. Rapid assessment of mental health of Detroit-area health care workers during the COVID-19 pandemic. Annals of Clinical Psychiatry. 2021;33(2):101-7.
106. Delaney AE, Dadlez NM, Marshall AC. Alternative approach to pediatric cardiac quality assessment for low-volume centers. Congenital Heart Disease. 2019;14(4):665-70.
107. Demeulemeester M, Lavis N, Balthazar Y, Lechien P, Heijmans S. Rapid safety assessment of a seasonal intradermal trivalent influenza vaccine. Hum Vaccin Immunother. 2017;13(4):889-94.
108. Douglas JE, Liu S, Ma J, Wolff RA, Pant S, Maitra A, et al. PIONEER-Panc: a platform trial for phase II randomized investigations of new and emerging therapies for localized pancreatic cancer. BMC Cancer. 2022;22(1):14.
109. Dudovitz RN, Russ S, Berghaus M, Iruka IU, DiBari J, Foney DM, et al. COVID-19 and Children's Well-Being: A Rapid Research Agenda. Maternal & Child Health Journal. 2021;25(11):1655-69.
110. Dunbar MS, Towe VL, Ayer L, Martineau M. Connections to Care (C2C): The Perspectives of Leaders at Community-Based Organizations That Are Integrating Mental Health Supports. Rand health q. 2018;7(4):7.

For peer review only

111. Eaves ER, Trotter RT, 2nd, Marquez B, Negron K, Doerry E, Mensah D, et al. Possibilities and constraints of rapid online ethnography: Lessons from a rapid assessment of COVID-19 policy for people who use drugs. Frontiers in Sociology. 2022;7:959642.
112. Elsawy W, Fouad H, Saeed K. Impact of COVID-19 on mental health and psychosocial support services in the Eastern Mediterranean Region - results of a rapid assessment. Eastern Mediterranean Health Journal. 2022;28(5):321-8.
113. Elwy AR, Clayman ML, LoBrutto L, Miano D, Ann Petrakis B, Javier S, et al. Vaccine hesitancy as an opportunity for engagement: A rapid qualitative study of patients and employees in the U.S. Veterans Affairs healthcare system. Vaccine: X. 2021;9:100116.
114. Gale RC, Wu J, Erhardt T, Bounthavong M, Reardon CM, Damschroder LJ, et al. Comparison of rapid vs in-depth qualitative analytic methods from a process evaluation of academic detailing in the Veterans Health Administration. Implementation Science. 2019;14(1):11.
115. Gagne JJ, Wang S, Schneeweiss S. A multivariable-adjusted rapid assessment of the association between zoledronic acid and myocardial infarction. Pharmacoepidemiology and Drug Safety. 2013;1):378.
116. Gomez-Ramirez O, Medeiros P, Wainer R, Iyamu I. Does the 'canada COVID-19 alert' app stand up to critical scrutiny? a rapid qualitative assessment. BMJ Open. 2021;11(SUPPL 1):A5.
117. Hamarman A, Mason P, Kroeger K, Alexander-Pender C. The impact of budget cuts among local STD programs in New Jersey: Results of a rapid ethnographic assessment. Sexually Transmitted Diseases. 2014;1):S93.
118. Hasford J. Large Simple Double-Blind Randomized Trials for the Rapid Assessment of the Effectiveness of COVID-19 Vaccines. Journal of Infectious Diseases. 2020;222(9):1571-2.
119. Hensel JM, Shaw J, Jeffs L, Ivers NM, Desveaux L, Cohen A, et al. A pragmatic randomized control trial and realist evaluation on the implementation and effectiveness of an internet application to support self-management among individuals seeking specialized mental health care: A study protocol. BMC Psychiatry. 2016;16 (1) (no pagination)(350).
120. Im H, George N. Impacts of COVID-19 on Refugee Service Provision and Community Support: A Rapid Assessment during the Pandemic. Social Work in Public Health. 2022;37(1):84-103.
121. Keniston A, Sakumoto M, Astik GJ, Auerbach A, Eid SM, Kangelaris KN, et al. Adaptability on Shifting Ground: a Rapid Qualitative Assessment of Multi-institutional Inpatient Surge Planning and Workforce Deployment During the COVID-19 Pandemic. Journal of General Internal Medicine. 2022;22:22.
122. Khalid-Khan S, Braund H, Dare J, Turnridge J, Dalgarno N. Evaluating Child and Adolescent Psychiatry sub-specialty program using rapid-cycle approach. Journal of the Canadian Academy of Child and Adolescent Psychiatry. 2020;29(4):263.
123. Khubchandani J, Sharma S, Price JH, Wiblishauser MJ, Sharma M, Webb FJ. COVID-19 Vaccination Hesitancy in the United States: A Rapid National Assessment. Journal of Community Health. 2021;46(2):270-7.
124. Kobau R, Carry M, Rubenstein BL, Denson D, Uribe C, Zajac J, et al. Implementing the COVID-19 Rapid Community Assessment on Vaccine Confidence: Lessons Learned From Alabama and Georgia. Public Health Reports. 2022;137(5):832-40.
125. Kuhlmann E, Brinzac MG, Burau V, Correia T, Ungureanu MI. Health workforce protection and preparedness during the COVID-19 pandemic: a tool for the rapid assessment of EU health systems. European Journal of Public Health. 2021;31(Supplement_4):iv14-iv20.
126. Livorsi D, Knobloch MJ, Blue LA, Swafford K, Maze L, Riggins K, et al. A rapid assessment of barriers and facilitators to safety culture in an intensive care unit. Int Nurs Rev. 2016;63(3):372-6.
127. Loosier PS, Carter MW, Hsu KK, Doshi S, Peterson Maddox BL, Kroeger K, et al. Provision of STD Services in Community Settings After the Loss and Return of State Funding to Support Service Provision: Observations From Select Providers in Massachusetts, 2010 and 2013. Journal of public health management and practice : JPHMP. 2020;26(1):E18-E27.

128. Maaskant JM, Jongerden IP, Bik J, Joosten M, Musters S, Storm-Versloot MN, et al. Strict isolation requires a different approach to the family of hospitalised patients with COVID-19: A rapid qualitative study. International journal of nursing studies. 2021;117:103858.
129. MacCarthy S, Barreras JL, Mendoza-Graf A, Galvan F, Linnemayr S. Strategies for Improving Mobile Technology-Based HIV Prevention Interventions With Latino Men Who Have Sex With Men and Latina Transgender Women. AIDS Educ Prev. 2019;31(5):407-20.
130. McDonald R, Eide D, Abel-Ollo K, Barnsdale L, Carter B, Clausen T, et al. A rapid assessment of take-home naloxone provision during COVID-19 in Europe. International Journal of Drug Policy. 2022;107:103787.
131. McNall MA, Welch VE, Ruh KL, Mildner CA, Soto T. The use of rapid-feedback evaluation methods to improve the retention rates of an HIV/AIDS healthcare intervention. Evaluation and Program Planning. 2004;27(3):287-94.
132. Minoyan N, Hoj SB, Zolopa C, Vlad D, Bruneau J, Larney S. Self-reported impacts of the COVID-19 pandemic among people who use drugs: a rapid assessment study in Montreal, Canada. Harm Reduction Journal. 2022;19(1):38.
133. Murray DD, Babiker AG, Baker JV, Barkauskas CE, Brown SM, Chang CC, et al. Design and implementation of an international, multi-arm, multi-stage platform master protocol for trials of novel SARS-CoV-2 antiviral agents: Therapeutics for Inpatients with COVID-19 (TICO/ACTIV-3). Clinical Trials. 2022;19(1):52-61.
134. Nevedal AL, Reardon CM, Opra Widerquist MA, Jackson GL, Cutrona SL, White BS, et al. Rapid versus traditional qualitative analysis using the Consolidated Framework for Implementation Research (CFIR). Implementation Science. 2021;16(1):67.
135. Palinkas LA, Whiteside L, Nehra D, Engstrom A, Taylor M, Moloney K, et al. Rapid ethnographic assessment of the COVID-19 pandemic April 2020 'surge' and its impact on service delivery in an Acute Care Medical Emergency Department and Trauma Center. BMJ Open. 2020;10(10):e041772.
136. Palinkas LA, De Leon J, Salinas E, Chu S, Hunter K, Marshall TM, et al. Impact of the COVID-19 pandemic on child and adolescent mental health policy and practice implementation. International Journal of Environmental Research and Public Health. 2021;18(18) (no pagination)(9622).
137. Palinkas LA, Springgate BF, Sugarman OK, Hancock J, Wennerstrom A, Haywood C, et al. A Rapid Assessment of Disaster Preparedness Needs and Resources during the COVID-19 Pandemic. International Journal of Environmental Research & Public Health [Electronic Resource]. 2021;18(2):07.
138. Rains E, Stuck A, Ko KJ, Crowley C, Zifferblatt J. Advancing alternative care pathways in geriatric acute unplanned care. Journal of the American Geriatrics Society. 2020;68(SUPPL 1):S130.
139. Renfro CP, Rome Z, Gatwood J, Hohmeier KC. Use of Rapid Assessment Procedures when analyzing qualitative data in pharmacy research. Research In Social & Administrative Pharmacy. 2022;18(1):2249-53.
140. Rodriguez R, Torres J, Chang A, Haggins A, Caldwell M, Miller D, et al. 54 The Rapid Evaluation of COVID-19 Vaccination in Emergency Departments for Underserved Patients Study. Annals of Emergency Medicine. 2021;78(2 Supplement):S28.
141. Rolf F, Campbell N, Thompson S, Argus G. Australians' Experience of the COVID-19 Pandemic: Advantages and Challenges of Scaling Up Qualitative Research Using Large-Scale Rapid Analysis and Building Research Capacity Across Rural Australia. International Journal of Qualitative Methods. 2021;20:16094069211051937.
142. Romeu-Labayen M, Tort-Nasarre G, Alvarez B, Subias-Miquel M, Vazquez-Segura E, Marre D, et al. Spanish nurses' experiences with personal protective equipment and perceptions of risk of contagion from COVID-19: A qualitative rapid appraisal. Journal of Clinical Nursing. 2022;31(15-16):2154-66.

For peer review only

143. Rosteius K, De Boer B, Staudacher S, Verbeek H. Building trusting relationships with staff members of nursing homes during rapid ethnographic research. Frontiers in Sociology. 2022;7:983728.
144. Scott K, Guigayoma J, Palinkas LA, Beaudoin FL, Clark MA, Becker SJ. The measurement-based care to opioid treatment programs project (MBC2OTP): a study protocol using rapid assessment procedure informed clinical ethnography. Addiction Science & Clinical Practice. 2022;17(1):44.
145. Seay J, Ranck A, Weiss R, Salgado C, Fein L, Kobetz E. Understanding Transgender Men's Experiences with and Preferences for Cervical Cancer Screening: A Rapid Assessment Survey. LGBT health. 2017;4(4):304-9.
146. Seay JS, Carrasquillo O, Campos NG, McCann S, Amofah A, Pierre L, et al. Cancer Screening Utilization Among Immigrant Women in Miami, Florida. Prog. 2015;9 Suppl:11-20.
147. Shaw J, Feeney H, Meunier-Sham J, Hazard K, Plante P, Petricone R. An evaluation of a rapid conversion to teleSANE in response to COVID-19. American Journal of Community Psychology. 2022;28:28.
148. Shimkhada R, Attai D, Scheitler AJ, Babey S, Glenn B, Ponce N. Using a Twitter Chat to Rapidly Identify Barriers and Policy Solutions for Metastatic Breast Cancer Care: Qualitative Study. JMIR Public Health and Surveillance. 2021;7(1):e23178.
149. Short NJ, Borthakur G, Pemmaraju N, Dinardo CD, Kadia TM, Jabbour E, et al. A multi-arm phase Ib/II study designed for rapid, parallel evaluation of novel immunotherapy combinations in relapsed/refractory acute myeloid leukemia. Leukemia & Lymphoma. 2022:1-10.
150. Solomon P, Tennille J, Lipsitt D, Plumb E, Metzger D, Blank MB. Rapid assessment of existing HIV prevention programming in a Community Mental Health Center. Journal of Prevention and Intervention in the Community. 2006;33(1-2):137-51.
151. Srinivasan M, Asch S, Vilendrer S, Thomas SC, Bajra R, Barman L, et al. Qualitative Assessment of Rapid System Transformation to Primary Care Video Visits at an Academic Medical Center. Annals of Internal Medicine. 2020;173(7):527-35.
152. Talati AN, Mallampati D, Johnson JD, West-Honart A, Vladutiu C, Menard MK. 1009 Provider satisfaction with telehealth for maternity care: a rapid assessment of a national survey. American Journal of Obstetrics and Gynecology. 2021;224(2 Supplement):S625.
153. Talwai A, Wing V, Itzkovich Y, Galaznik A, Chatterjee A, Jain R, et al. PIN83 The COVID-19 Research Database: Building One of the Largest PRO Bono Real-World DATA Repositories. Value in Health. 2021;24(Supplement 1):S121.
154. Tan RKJ, Lim JM, Lo JJ, Teo AKJ, O'Hara CA, Ching AH, et al. Conducting rapid qualitative research to support sex workers' health and social needs in the face of COVID-19: capitalising on stakeholder networks from the HIV response in Singapore to drive policymaking. Sexually Transmitted Infections. 2021;97(2):84.
155. Taubenberger S, Spencer N, Chang JC, Paul N, Fabre S, Jagessar B, et al. A rapid-cycle assessment strategy for understanding the opioid overdose epidemic in local communities. Substance Abuse. 2021;42(4):888-95.
156. Tort-Nasarre G, Alvarez B, Galbany-Estragues P, Subias-Miquel M, Vazquez-Segura E, Marre D, et al. Front-line nurses' responses to organisational changes during the COVID-19 in Spain: A qualitative rapid appraisal. Journal of Nursing Management. 2021;29(7):1983-91.
157. Toth G, Limburg H, Szabo D, Sandor GL, Nagy ZZ, Nemeth J. Rapid assessment of avoidable blindness-based healthcare costs of diabetic retinopathy in Hungary and its projection for the year 2045. British Journal of Ophthalmology. 2021;105(8):1116-20.
158. Vazquez E, Chobdee J, Nasrollahzadeh N, Cheney A. Personal Freedom and Social Responsibility in Slowing the Spread of COVID-19: A Rapid Qualitative Study. Health Education & Behavior. 2022;49(1):26-34.

For peer review only

159. Wesolowski A, Gibson D, Agarwal S, Lambrou A, Kirk G, Labrique A, et al. Rapid assessments of non-pharmaceutical intervention uptake and population mobility patterns elucidate SARS-CoV-2 transmission dynamics. Open Forum Infectious Diseases. 2020;7(SUPPL 1):S848.
160. Wichmann O, Stocker P, Poggensee G, Altmann D, Walter D, Hellenbrand W, et al. Pandemic influenza A(H1N1) 2009 breakthrough infections and estimates of vaccine effectiveness in Germany 2009-2010. Eurosurveillance. 2010;15(18):1-4.
161. Porter A, Black S, Dale J, Harris-Mayes R, Lawrenson R, Lyons R, et al. Electronic records in ambulances-an observational study (ERA). Emergency Medicine Journal. 2019;36 (10):E14.
162. Wiss DA, Schellenberger M, Prelip ML. Rapid Assessment of Nutrition Services in Los Angeles Substance Use Disorder Treatment Centers. Journal of Community Health. 2019;44(1):88-94.
163. Xiang JJ, Roy A, Summers C, Delvy M, O'Donovan JL, Christensen J, et al. Improving hematology/oncology clinical research recruitment via universal prescreening: The VA Connecticut (VACT) Cancer Center experience. Journal of Clinical Oncology Conference. 2021;39(28 SUPPL).
164. Agaku IT, Nkosi L, Agaku QD, Gwar J, Tsafa T. A Rapid Evaluation of the US Federal Tobacco 21 (T21) Law and Lessons From Statewide T21 Policies: Findings From Population-Level Surveys. Preventing Chronic Disease. 2022;19:E29.
165. Ash JS, Sittig DF, McMullen CK, Guappone K, Dykstra R, Carpenter J. A rapid assessment process for clinical informatics interventions. Amia 2008;Annual Symposium proceedings / AMIA Symposium. AMIA Symposium.:26-30.
166. Belford M, Robertson T, Jepson R. Using evaluability assessment to assess local community development health programmes: a Scottish case-study. BMC medical research methodology. 2017;17(1):70.
167. Haigh F, Harris E, Chok HN, Baum F, Harris-Roxas B, Kemp L, et al. Characteristics of health impact assessments reported in Australia and New Zealand 2005-2009. Australian and New Zealand journal of public health. 2013;37(6):534-46.
168. Hailey D. The use and impact of rapid health technology assessments. 2001.
169. Lewinski AA, Crowley MJ, Miller C, Bosworth HB, Jackson GL, Steinhauser K, et al. Applied Rapid Qualitative Analysis to Develop a Contextually Appropriate Intervention and Increase the Likelihood of Uptake. Medical Care. 2021;59(Suppl 3):S242-S51.
170. Li Y, Li M, Rice M, Su Y, Yang C. Phased Implementation of COVID-19 Vaccination: Rapid Assessment of Policy Adoption, Reach and Effectiveness to Protect the Most Vulnerable in the US. International Journal of Environmental Research & Public Health [Electronic Resource]. 2021;18(14):19.

For peer review only

Preferred Reporting Items for Systematic reviews and Meta-Analyses extension for Scoping Reviews (PRISMA-ScR) Checklist

SECTION	ITEM	PRISMA-ScR CHECKLIST ITEM	REPORTED ON PAGE #
**TITLE**			
Title	1	Identify the report as a scoping review.	1
**ABSTRACT**			
Structured summary	2	Provide a structured summary that includes (as applicable): background, objectives, eligibility criteria, sources of evidence, charting methods, results, and conclusions that relate to the review questions and objectives.	2
**INTRODUCTION**			
Rationale	3	Describe the rationale for the review in the context of what is already known. Explain why the review questions/objectives lend themselves to a scoping review approach.	3-4
Objectives	4	Provide an explicit statement of the questions and objectives being addressed with reference to their key elements (e.g., population or participants, concepts, and context) or other relevant key elements used to conceptualize the review questions and/or objectives.	4
**METHODS**			
Protocol and registration	5	Indicate whether a review protocol exists; state if and where it can be accessed (e.g., a Web address); and if available, provide registration information, including the registration number.	2, 5
Eligibility criteria	6	Specify characteristics of the sources of evidence used as eligibility criteria (e.g., years considered, language, and publication status), and provide a rationale.	5, 6
Information sources*	7	Describe all information sources in the search (e.g., databases with dates of coverage and contact with authors to identify additional sources), as well as the date the most recent search was executed.	5,6
Search	8	Present the full electronic search strategy for at least 1 database, including any limits used, such that it could be repeated.	Supplementary material
Selection of sources of evidence†	9	State the process for selecting sources of evidence (i.e., screening and eligibility) included in the scoping review.	7
Data charting process‡	10	Describe the methods of charting data from the included sources of evidence (e.g., calibrated forms or forms that have been tested by the team before their use, and whether data charting was done independently or in duplicate) and any processes for obtaining and confirming data from investigators.	7
Data items	11	List and define all variables for which data were sought and any assumptions and simplifications made.	7

SECTION	ITEM	PRISMA-ScR CHECKLIST ITEM	REPORTED ON PAGE #
Critical appraisal of individual sources of evidence§	12	If done, provide a rationale for conducting a critical appraisal of included sources of evidence; describe the methods used and how this information was used in any data synthesis (if appropriate).	N/A
Synthesis of results	13	Describe the methods of handling and summarizing the data that were charted.	7,8
**RESULTS**			
Selection of sources of evidence	14	Give numbers of sources of evidence screened, assessed for eligibility, and included in the review, with reasons for exclusions at each stage, ideally using a flow diagram.	8 (Figure 1)
Characteristics of sources of evidence	15	For each source of evidence, present characteristics for which data were charted and provide the citations.	Multiple tables, supplementary information
Critical appraisal within sources of evidence	16	If done, present data on critical appraisal of included sources of evidence (see item 12).	N/A
Results of individual sources of evidence	17	For each included source of evidence, present the relevant data that were charted that relate to the review questions and objectives.	P10, Table 2; supplementary information
Synthesis of results	18	Summarize and/or present the charting results as they relate to the review questions and objectives.	P10-23, Tables 2-5
**DISCUSSION**			
Summary of evidence	19	Summarize the main results (including an overview of concepts, themes, and types of evidence available), link to the review questions and objectives, and consider the relevance to key groups.	23-24
Limitations	20	Discuss the limitations of the scoping review process.	25-26
Conclusions	21	Provide a general interpretation of the results with respect to the review questions and objectives, as well as potential implications and/or next steps.	27-28
**FUNDING**			
Funding	22	Describe sources of funding for the included sources of evidence, as well as sources of funding for the scoping review. Describe the role of the funders of the scoping review.	29

JBI = Joanna Briggs Institute; PRISMA-ScR = Preferred Reporting Items for Systematic reviews and Meta-Analyses extension for Scoping Reviews.
* Where *sources of evidence* (see second footnote) are compiled from, such as bibliographic databases, social media platforms, and Web sites.
† A more inclusive/heterogeneous term used to account for the different types of evidence or data sources (e.g., quantitative and/or qualitative research, expert opinion, and policy documents) that may be eligible in a scoping review as opposed to only studies. This is not to be confused with *information sources* (see first footnote).
‡ The frameworks by Arksey and O'Malley (6) and Levac and colleagues (7) and the JBI guidance (4, 5) refer to the process of data extraction in a scoping review as data charting.
§ The process of systematically examining research evidence to assess its validity, results, and relevance before using it to inform a decision. This term is used for items 12 and 19 instead of "risk of bias" (which is more applicable to systematic reviews of interventions) to include and acknowledge the various sources of evidence that may be used in a scoping review (e.g., quantitative and/or qualitative research, expert opinion, and policy document).

For peer review only

From: Tricco AC, Lillie E, Zarin W, O'Brien KK, Colquhoun H, Levac D, et al. PRISMA Extension for Scoping Reviews (PRISMAScR): Checklist and Explanation. Ann Intern Med. 2018;169:467–473. doi: 10.7326/M18-0850.

For peer review only
